# Development and analysis of a comprehensive diagnostic model for aortic valve calcification using machine learning methods and artificial neural networks

**DOI:** 10.3389/fcvm.2022.913776

**Published:** 2022-12-01

**Authors:** Tao Xiong, Yan Chen, Shen Han, Tian-Chen Zhang, Lei Pu, Yu-Xin Fan, Wei-Chen Fan, Ya-Yong Zhang, Ya-Xiong Li

**Affiliations:** ^1^Department of Cardiovascular Surgery, Yan’an Affiliated Hospital of Kunming Medical University, Kunming, Yunnan, China; ^2^Key Laboratory of Cardiovascular Disease of Yunnan Province, Yan’an Affiliated Hospital of Kunming Medical University, Kunming, Yunnan, China

**Keywords:** aortic valve calcification, diagnostic model, machine learning, immune infiltration, diagnostic marker

## Abstract

**Background:**

Although advanced surgical and interventional treatments are available for advanced aortic valve calcification (AVC) with severe clinical symptoms, early diagnosis, and intervention is critical in order to reduce calcification progression and improve patient prognosis. The aim of this study was to develop therapeutic targets for improving outcomes for patients with AVC.

**Materials and methods:**

We used the public expression profiles of individuals with AVC (GSE12644 and GSE51472) to identify potential diagnostic markers. First, the R software was used to identify differentially expressed genes (DEGs) and perform functional enrichment analysis. Next, we combined bioinformatics techniques with machine learning methodologies such as random forest algorithms and support vector machines to screen for and identify diagnostic markers of AVC. Subsequently, artificial neural networks were employed to filter and model the diagnostic characteristics for AVC incidence. The diagnostic values were determined using the receiver operating characteristic (ROC) curves. Furthermore, CIBERSORT immune infiltration analysis was used to determine the expression of different immune cells in the AVC. Finally, the CMap database was used to predict candidate small compounds as prospective AVC therapeutics.

**Results:**

A total of 78 strong DEGs were identified. The leukocyte migration and pid integrin 1 pathways were highly enriched for AVC-specific DEGs. CXCL16, GPM6A, BEX2, S100A9, and SCARA5 genes were all regarded diagnostic markers for AVC. The model was effectively constructed using a molecular diagnostic score system with significant diagnostic value (AUC = 0.987) and verified using the independent dataset GSE83453 (AUC = 0.986). Immune cell infiltration research revealed that B cell naive, B cell memory, plasma cells, NK cell activated, monocytes, and macrophage M0 may be involved in the development of AVC. Additionally, all diagnostic characteristics may have varying degrees of correlation with immune cells. The most promising small molecule medicines for reversing AVC gene expression are Doxazosin and Terfenadine.

**Conclusion:**

It was identified that CXCL16, GPM6A, BEX2, S100A9, and SCARA5 are potentially beneficial for diagnosing and treating AVC. A diagnostic model was constructed based on a molecular prognostic score system using machine learning. The aforementioned immune cell infiltration may have a significant influence on the development and incidence of AVC.

## Introduction

Aortic valve calcification (AVC) is the most prevalent type of valvular heart disease and a major cause of morbidity and mortality (1, 2). It is an aggressive disease that begins with changes in valve cell biology and proceeds to leaflet thickening, neovascularization, and calcium deposition, eventually causing calcific aortic stenosis (3–5). The most common cause of aortic stenosis is calcific aortic valve disease, which affects one in every four people over the age of 65 (6, 7). The burden of AVC is predicted to grow from 2.5 million in 2000 to 4.5 million by 2030 with increased life expectancy and aging population (8), imposing a considerable economic and health cost globally (9, 10). There is no effective treatment for symptomatic aortic valve stenosis other than surgical or interventional valve replacement because pharmaceutical therapies have not been proved to be effective (11–13). Consequently, there is a clinical need for a better understanding of the underlying processes of AVC, as well as the identification of novel treatment targets for slowing its development. Therefore, identifying critical genes, biomarkers, and pathways is critical for early detection, prevention, and precision therapy.

Aortic stenosis was once considered a degenerative disorder caused by “wear and tear” on the valve, resulting in gradual calcium buildup inside the valve. Although shear pressure and injury play a role in the development of aortic stenosis, new evidence suggests that it is caused by an extremely complicated and strictly regulated series of activities, each of which may be conducive to medical intervention if detected in the early stages (14). The progression of aortic valve stenosis can be divided into two stages: the early stage, which is similar to atherosclerosis and involves lipid deposition in the valve, as well as injury and inflammation; the late stage, which involves the appearance of pro-valvular calcification and bone-promoting factors (15). The constricted aortic orifice hinders cardiac output as valvular fibrosis and calcification progress, resulting in angina, chest discomfort, and decreased exercise tolerance. The ultimate outcome is to lead to left ventricular remodelling, which necessitates aortic valve replacement or death. Surgery and transcatheter aortic valve replacement (TAVR) are the most effective treatment when AVC reaches the terminal stage with severe clinical symptoms. However, the operation is costly, and it is accompanied by risks associated with postoperative valve and anticoagulation, such as reoperation of artificial valve insufficiency, bleeding, and embolism (6).

Machine learning approaches such as random forest (RF), support vector machine-recursive feature elimination (SVM-RFE), and artificial neural networks (ANN) are effective in identifying and researching biomarkers for a variety of diseases (16, 17). With the advancement of machine learning, it is now possible to choose and transform the most significant differentially expressed genes (DEGs) into statistical models that can assist clinicians in picking sensible and beneficial treatment strategies (18). Therefore, it is critical to gain a better understanding of the underlying processes of AVC and identify potential treatment targets. In recent years, high-throughput sequencing data generated using microarray technology have aided in the identification of DEGs and their roles, as well as the deciphering of the pathways *via* which they are linked to the advancement of many complex disorders (19). Secondary data mining is enabled by a comprehensive bioinformatics analysis of publicly available genetic data, which allows us to identify biomarkers associated with illnesses (20). Accordingly, the purpose of this work was to construct and test a machine learning-based diagnostic model for AVC patients, as well as to get thorough knowledge of the immunological processes underlying AVC development and to screen potential small molecule medicines.

## Materials and methods

### Downloading and processing data

In this study, the GSE12644 (21) and GSE51472 (22) were retrieved from GEO^1^ (23) and both were derived from the GPL570 platform of the 84 Affymetrix Human Genome U133 Plus 2.0 Aray. The GSE12644 dataset contains 10 AVC and 10 normal samples, which were collected from the aortic valve. The GSE51472 dataset contains 10 AVC and 5 normal samples, which were collected from the aortic valve. The probes in each dataset were converted to gene symbols using the probe annotation files provided by the researchers and the results were analyzed. When several probes correspond to the same gene symbol, we choose the average of the expression levels of the genes. Expression values were log2 transformed for the GSE51472 dataset. In addition, since both GSE12644 and GSE51472 share a common platform, it was advantageous for data merging. Using the “sva” package, the two datasets were combined together to form a metadata dataset and normalized for conducting integration studies (24). We used the ComBat function to eliminate batch effects between the two datasets (25). We used principal component analysis (PCA) (26) to determine if batch effects had been eliminated. The AVC dataset (GSE83453) (27) was also identified from the GEO database and served as the validation cohort, including 9 AVC samples and 8 normal samples. Table 1 contains information of the GSE12644, GSE51472, and GSE83453 datasets.

**TABLE 1:** The characteristic of three datasets.

Datasets	AVC		Normal		Platform
		
	Female	Male	Female	Male	
GSE12644	0	10	0	10	GPL570
GSE51472	5		10		GPL570
GSE83453	0	9	0	8	GPL10558

AVC, aortic valve calcification.

### Differential expression analysis

The “limma” package (28) was used to filter the DEGs for the combined dataset. The “ggplot2” package was used to visualize differential expression of the DEGs using heat map and volcano plot. The DEGs were deemed statistically significant in this investigation when their *P* < 0.05 and |log2FC| > 0.585.

### Functional and pathway enrichment analysis of differentially expressed genes

Metascape^2^ is used to analyze pathway enrichment and annotate biological processes in order to completely comprehend the information contained in each gene (29). In this study, Metascape was used to conduct gene ontology and pathway enrichment analyses on the DEGs from the merged dataset to identify the most significant functional biological terms and signaling pathways. Statistical significance was determined based on the number of enriched genes ≥ 3 and *P* < 0.01. Then, all key terms were clustered based on membership similarity and the term with the greatest degree of enrichment was selected to represent the cluster. The “clusterProfiler” package was used to assess gene ontology (GO) and Kyoto Encyclopedia of Genes and Genomes (KEGG) pathway enrichment to reveal the functions and pathways of distinctive genes. The q value < 0.05 and adjusted *P*-value < 0.05 were considered significantly enriched.

### Key gene screening and diagnostic effectiveness

RF (30) and SVM-RFE (31) were used to scan for unique and critical AVC biomarkers. The “randomForest” package was used to generate a random forest model for the DEGs. First, the appropriate amounts of variables (mtry parameter, the optimal number of variables used in the binary tree for a given node) was established. The optimal number of trees was 500 in the random forest. Next, a random forest model was built, and its dimensionality significance value was evaluated using the decreasing accuracy approach (Gini coefficient method). Disease-specific genes with a significance value > 2 were selected. Support vector machine (SVM) is a classification and regression technique that is often used in supervised machine learning, and the RFE technology is used to choose the best gene from the metadata queue in order to reduce overfitting. Therefore, SVM-RFE was used to find the set of genes with the greatest discriminative potential. Then, the intersecting genes from the two categorization models were chosen for further investigation and represented using venn diagrams and heat maps to show variations in normal and AVC samples of the merged dataset. The validity of important biomarkers was assessed using a validation dataset (GSE83453) and the difference between normal and AVC samples was shown in box plot graphs. The receiver operating characteristic (ROC) curves and the area under the curve (AUC) were used to determine the diagnostic capability of genes. Statistical significance was established by a two-sided *P* < 0.05.

### Aortic valve calcification diagnosis using machine learning

Five markers were obtained by combining two machine learning techniques. We first obtained the logFC values of the five genes in all samples of the combined dataset of GSE12644 and GSE51472 datasets (15 normal and 20 AVC samples in total) for the next analysis. Genes with logFC > 0 were upregulated genes and genes with logFC < 0 were downregulated genes. Subsequently, The expression values of the five markers were translated into a score table named “Gene Score” by using the “BiocManager” package (32). The specific conversion rules were as follows: if the expression levels of an upregulated gene in a sample were greater than the median expression levels of the gene across all samples, the gene score of the upregulated gene was transformed to 1, else it was transformed to 0. If the expression levels of a downregulated gene in a sample was greater than the median expression levels of the gene across all samples, the gene score of the upregulated gene transformed to 0, else it was transformed to 1. Generally, the gene score was composed of 35 lines of samples and five columns of DEGs. The results of gene score are described in Table 2.

**TABLE 2:** Diagnostic efficacy of five genes.

Genes	Diagnostic efficacy (Area under curve)	
	
	Merged dataset	GSE83453
CXCL16	0.953	0.861
GPM6A	0.897	0.944
BEX2	0.867	0.861
S100A9	0.897	0.875
SCARA5	0.887	0.931

Finally, we used the R-based “NeuralNetTools” and “neuralnet” packages to build the neural network diagnostic model and set the seed = 12345678. The diagnosis features of samples and the gene score for each of the five diagnostic markers were denoted by y and x, respectively. The data for building the model was gene score for all samples of the combined dataset of GSE12644 and GSE51472 datasets. The ANN consisted of three layers: an input layer, a hidden layer, and an output layer. We created five hidden nodes in the hidden layer and used the rectified linear unit as an activation function. In the output layer, we defined two nodes (normal and AVC) and the activation function of each node was a softmax function. The cross-entropy error was defined as a loss function, and the “rprop+” algorithm was used to optimize the weight values. After training, we choose the maximum weight value for a particular marker in the hidden layer named “Gene Weight” (33).

### Development and validation of diagnostic models

The model for diagnostic AVC patients was built. The diagnostic model was successful in diagnosing responsiveness of drugs and ulcerative colitis (34, 35). The GSE83453 dataset were used to validate the effectiveness of the diagnostic model based on the training dataset (the combined dataset of GSE12644 and GSE51472). According to the conversion rules, we obtained an updated “Gene Score” and calculated the summation of “Gene Score” × “Gene Weight”. To determine the diagnostic accuracy of this model, the area under the receiver operating characteristic curve (ROC) was determined using the “ROC” package. The distinction was considered good when the AUC value was between 0.8 and 9, and exceptional when the AUC value was > 0.9 (36).

### A correlation analysis of 22 immune cells associated with immune infiltration

The CIBERSORT site was used to screen immune cell matrices. To generate immunocell infiltration matrices, *P* < 0.05 was used. A PCA clustering analysis of the immune cell infiltration matrix was performed using the “ggplot2” package, whereas the “corrplot” package was used to generate a correlation heat map to visualize the association between 22 infiltrating immune cells. Furthermore, the “ggplot2” package was used to analyze and visualize the Spearman correlation between distinctive diagnostic markers and immune infiltration cells.

### Small molecule screening

The profiles of the five marker genes were analyzed using the CMap database^3^ to identify prospective medications that could reverse the consequences of AVC. First, the five marker genes were divided into two groups: upregulated and down-regulated. Next, genes from both groups were uploaded into the CMAP database to identify potential small therapeutic compounds, with a cut-off threshold of *P* < 0.05 regarded significant. After gathering all the data, the enrichment scores (–1 to 1) were computed to determine how closely genes and medications matched. An enrichment score > 0 indicated the presence of potential synergistic effects to AVC, a sign that the molecules were able to mimic the biological status of AVC. An enrichment score < 0 may be meant as a potential therapeutic drug.

## Results

**FIGURE 1:**
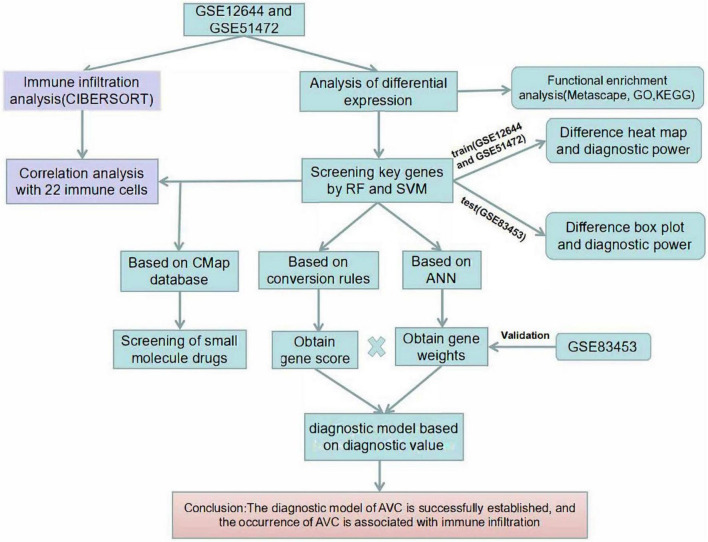
Flow chart of the research process.

The procedures of our cohort research are shown in Figure 1. The training datasets (GSE12644 and GSE51472) and validation dataset (GSE83453) for AVC are described in Table 1.

### Identification of differentially expressed genes

**FIGURE 2:**
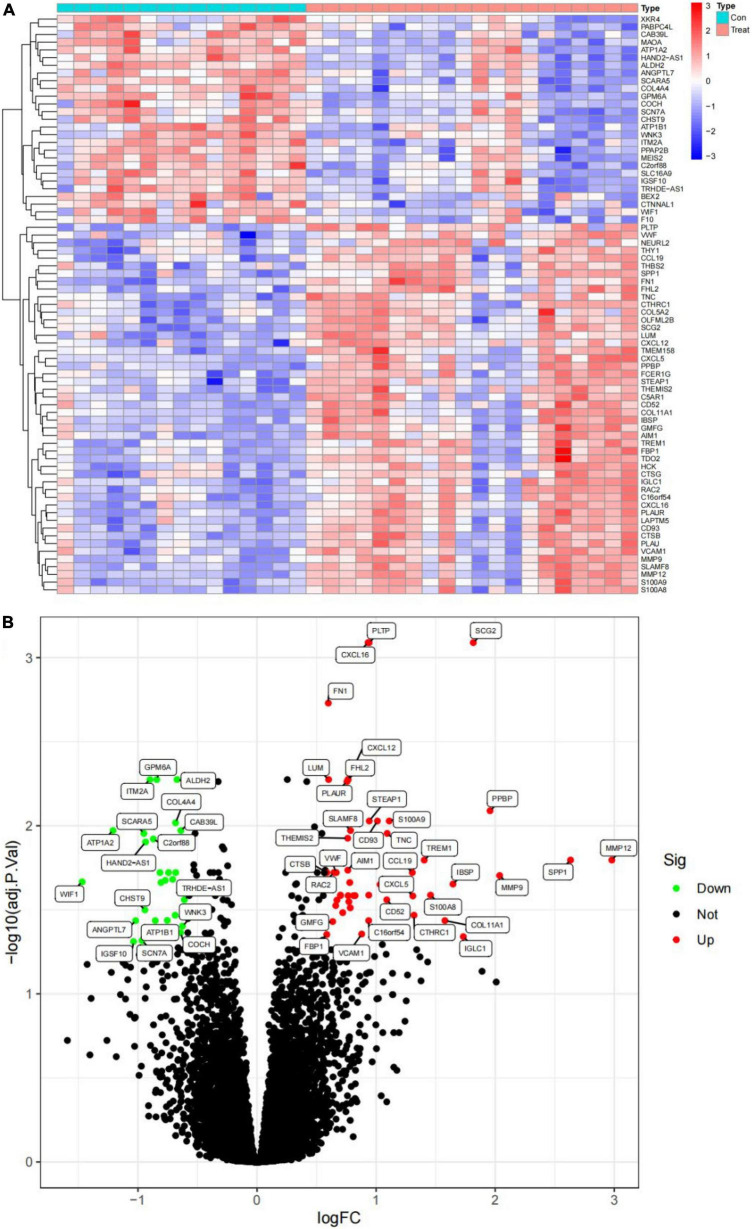
Visualization of differential analysis. **(A)** Heat maps of DEGs in AVC and normal samples. Red represents AVC samples, and blue represents normal samples. Blue indicates that the relative gene expression level is low, while red indicates that the relative gene expression level is high. **(B)** The volcano map illustrates the distribution of DEGs in the combined data set. Red indicates a high level of DEGs expression, and green indicates a low level of DEGs expression.

The gene expression levels of combined GEO series that were handled for batch effects were standardized, and the findings of pre- and post-standardized experiments are shown in Supplementary Figures 1A,B. A total of 21,654 genes were recognized in the GSE12644 and GSE51472 datasets, and differentially expressed genes (DEGs) associated with AVC were verified. A total of 75 DEGs, including 48 upregulated genes and 27 downregulated genes, were found in the AVC samples compared to the normal samples (| log2 FC| > 0.585 and *P* < 0.05) (Supplementary Table 1). Figures 2A,B depict a heat map plot and a volcano plot of 75 DEGs that were included in the subsequent studies.

### Functional enrichment analysis of the differentially expressed genes in the training dataset

**FIGURE 3:**
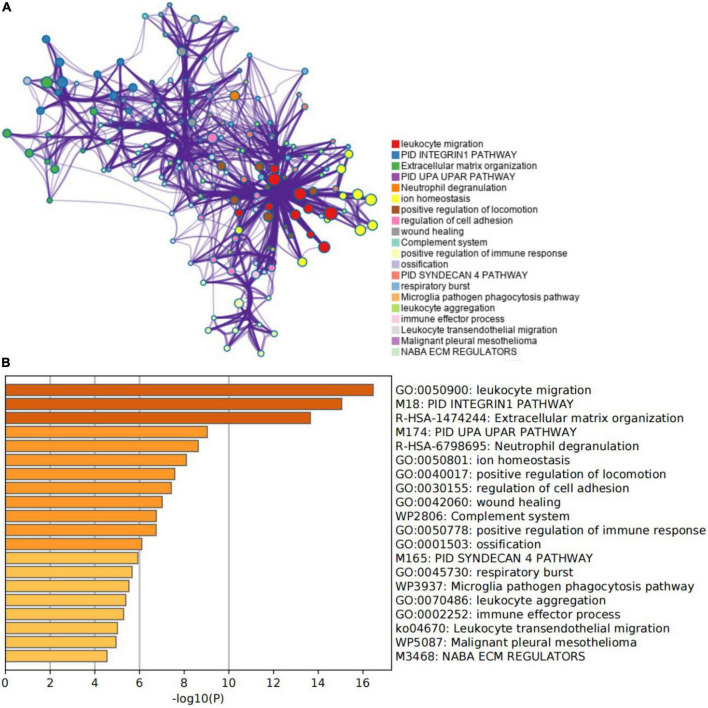
Gene function enrichment analysis results. **(A)** The network of the top 20 enriched term clusters. The color indicates cluster identification, the thickness of the edge indicates the similarity score, and terms with a similarity score > 0.3 are connected by an edge. **(B)** The top 20 clusters are shown in a heat map. Color is used to indicate cluster identification; the lower the p-value, the darker the color.

**FIGURE 4:**
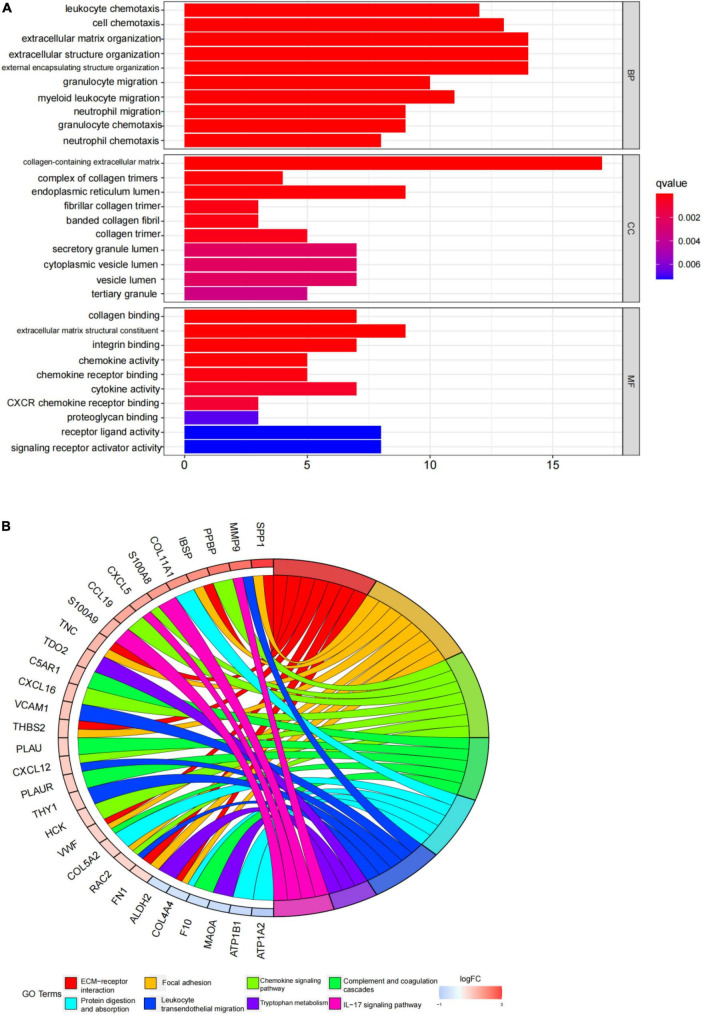
Gene ontology (GO) and kyoto encyclopedia of genes and genomes (KEGG) enrichment analysis results. **(A)** The top 10 items from the GO pathway enrichment studies are shown in the form of a bar plot. Statistical significance was defined as a *P*-value < 0.05. BP denotes biological processes; CC denotes cellular components, and MF denotes molecular function. **(B)** The top eight terms from the KEGG pathway enrichment studies are shown in the form of a circle plot. A *p*-value < 0.05 was considered statistically significant.

An enrichment analysis was conducted using Metascape to identify the top 20 clusters with substantial DEGs enrichment to gain a better understanding of the functional and metabolic pathways associated with these DEGs (Figures 3A,B and Supplementary Table 2). Leukocyte migration, the pid integrin1 pathway, and extracellular matrix organization were the most enriched gene ontology terms for biological processes. Gene Ontology (GO) projects included biological process (BP), cellular component (CC), and molecular function (MF) (Figure 4A and Supplementary Table 3). The major enrichments in BP were leukocyte chemotaxis, cell chemotaxis, and extracellular matrix organization; the major enrichments in CC were collagen-containing extracellular matrix, complex of collagen trimers, and endoplasmic reticulum lumen; and the major enrichments in MF were collagen binding and extracellular matrix structural organization. Additionally, KEGG enrichment analysis revealed considerable enrichment in the extracellular matrix-receptor interaction, focal adhesion, and chemokine signaling pathways (Figure 4B and Supplementary Table 4). The inhibitors of the enriched terms and pathways, which were more involved in AVC, may be investigated as additional therapy options for AVC patients.

### Screening and diagnostic efficacy of key genes

**FIGURE 5:**
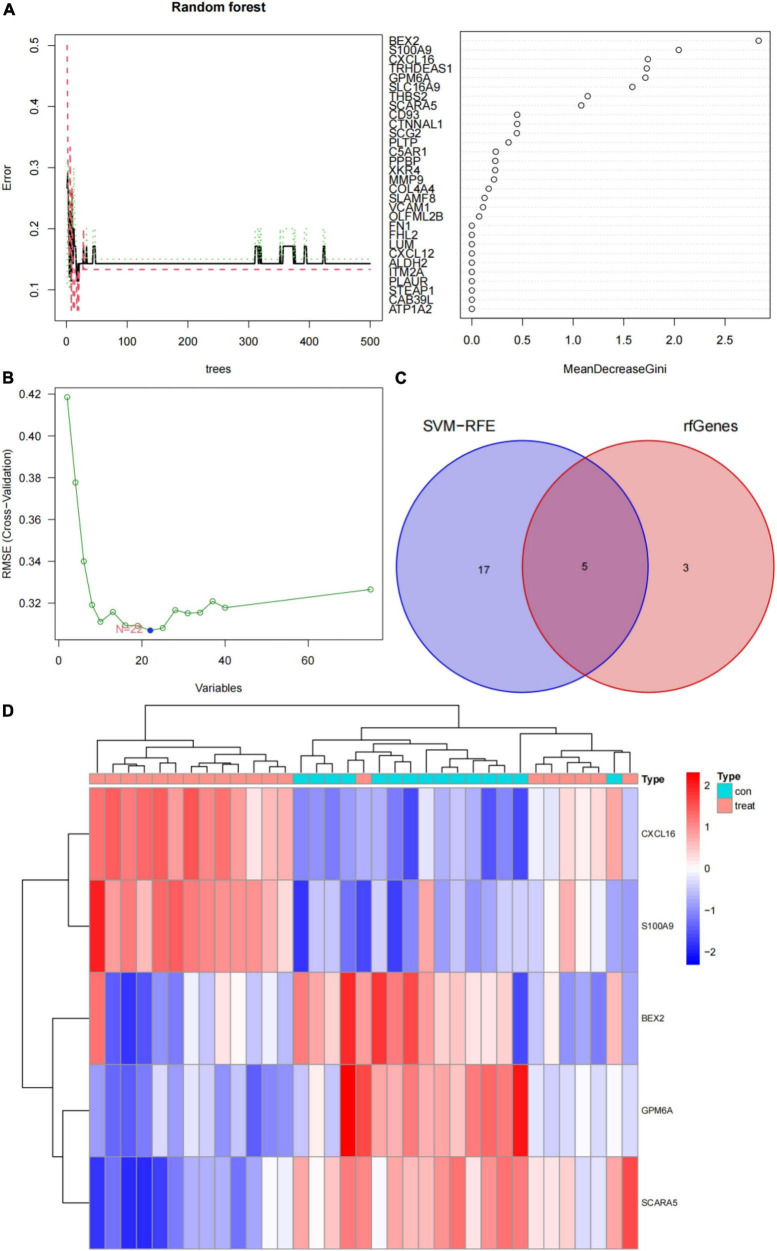
Screening for diagnostic markers using a comprehensive technique and five markers were heat mapped in AVC and normal samples. **(A)** Random forest (RF) algorithm to screen for diagnostic markers. **(B)** Support vector machine-recursive feature elimination (SVM-RFE) algorithm to screen for diagnostic markers. **(C)** Venn diagram illustrating the intersection of diagnostic markers acquired using two methods. **(D)** Each row corresponds to a single sample, whereas each column corresponds to a particular gene. The red color denotes AVC samples, while the blue hue denotes normal samples. The color scale indicates the relative degree of gene expression in a particular slide: Blue denotes low levels of relative expression, while red shows high levels of relative expression.

**FIGURE 6:**
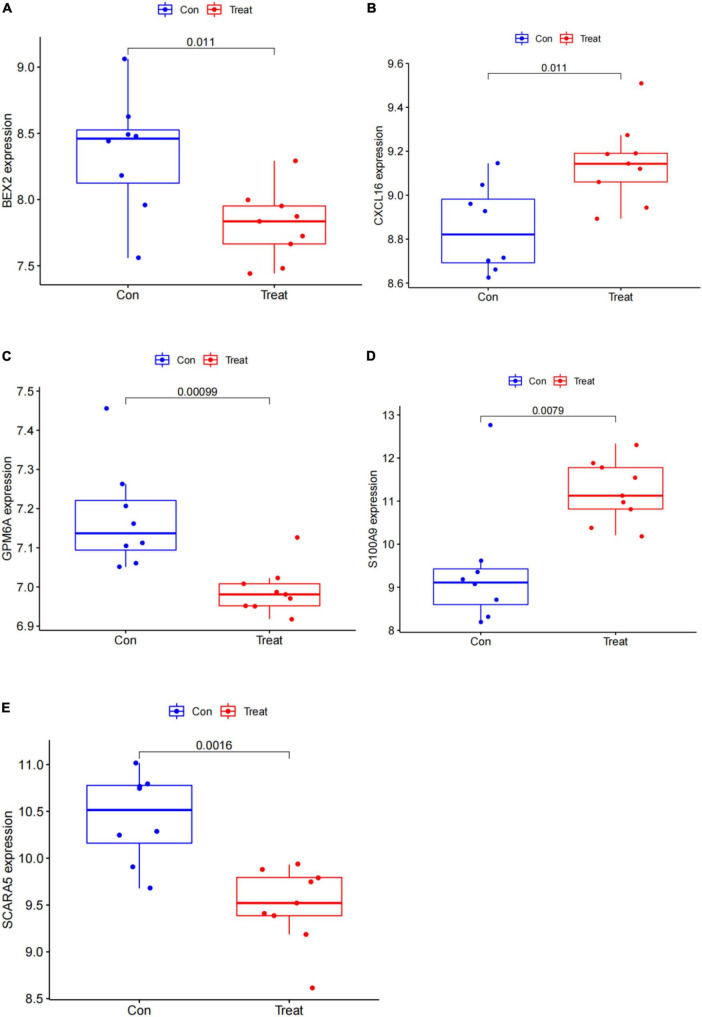
Gene expression of five markers is visualized using a box plot in the test set. **(A)** Box plot of BEX2 expression in the test set. **(B)** Box plot of CXCL16 expression in the test set. **(C)** Box plot of GPM6A expression in the test set. **(D)** Box plot of S100A9 expression in the test set. **(E)** Box plot of SCARA5 expression in the test set.

**FIGURE 7:**
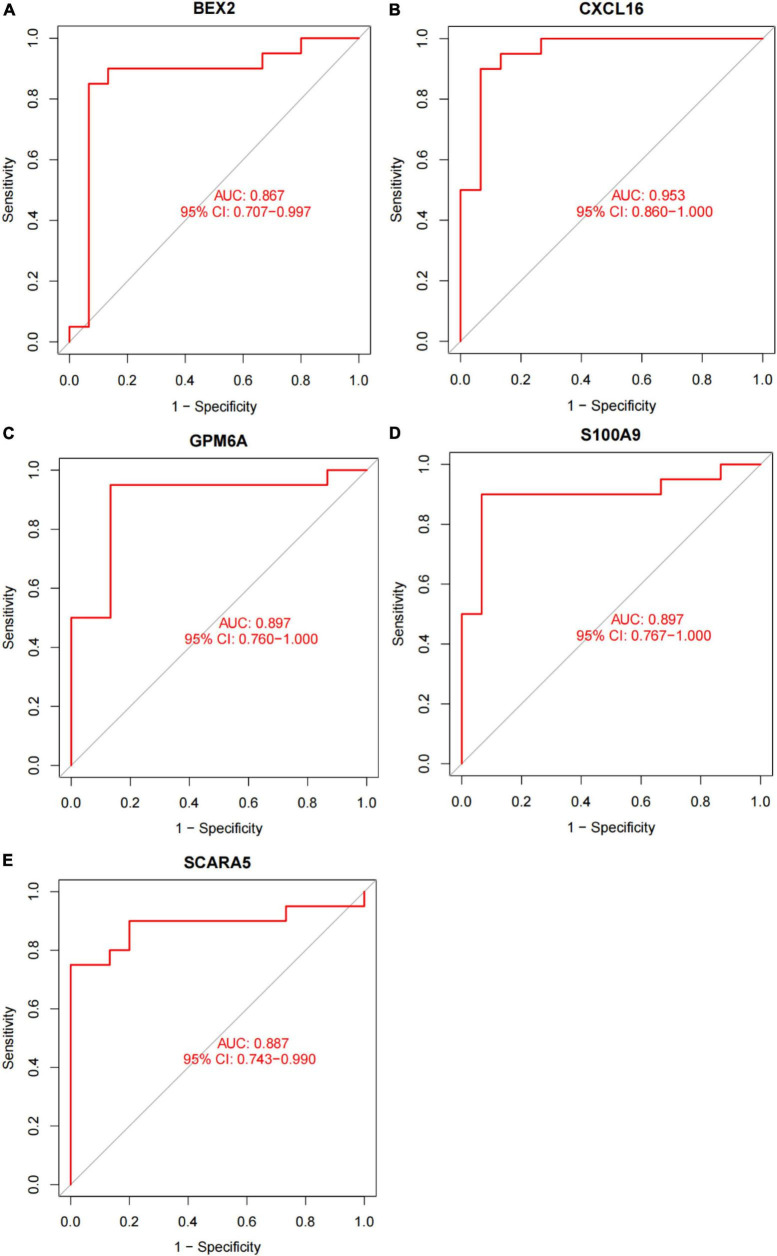
The diagnostic effect of five markers in the training set. **(A)** The diagnostic effect of BEX2 in the training set. **(B)** The diagnostic effect of CXCL16 in the training set. **(C)** The diagnostic effect of GPM6A in the training set. **(D)** The diagnostic effect of S100A9 in the training set. **(E)** The diagnostic effect of SCARA5 in the training set. The points marked on the ROC curve are the optimal threshold points, and the values in parentheses represent sensitivity and specificity. The AUC value is the area under the ROC curve.

**FIGURE 8:**
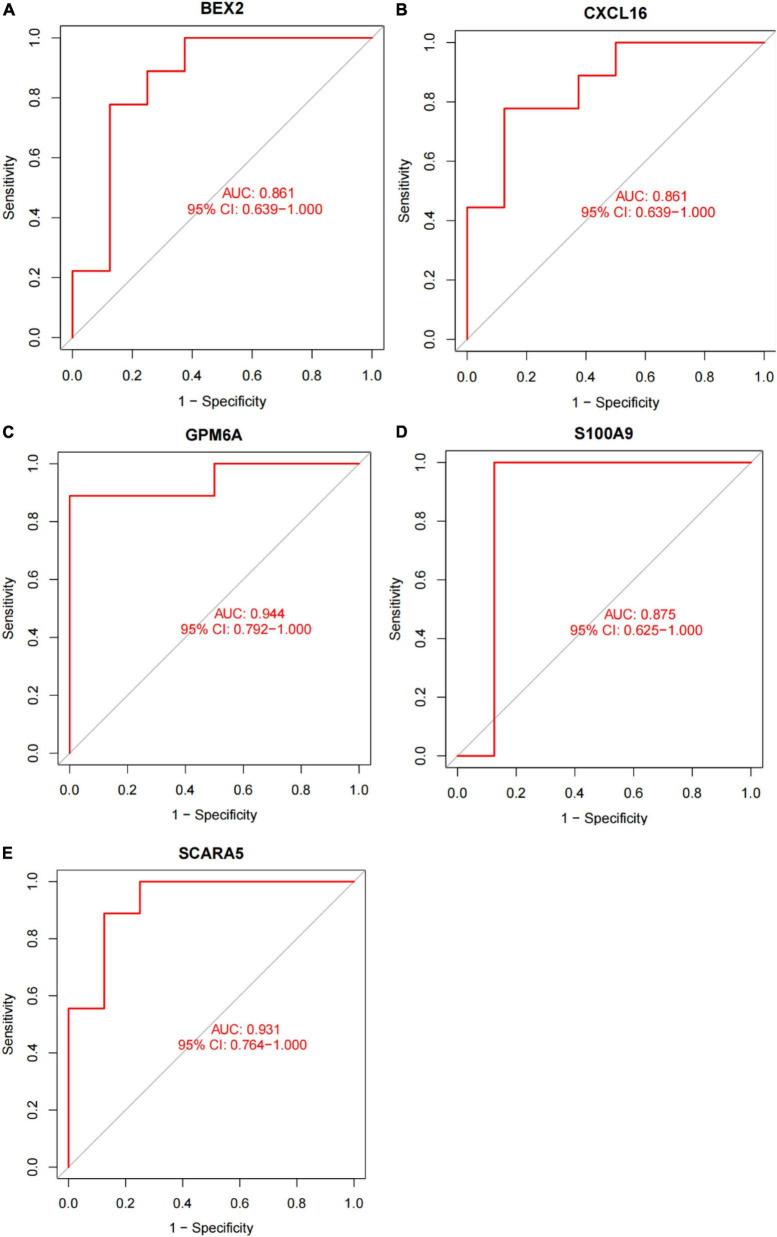
The diagnostic effect of five markers in the test set. **(A)** The diagnostic effect of BEX2 in the test set. **(B)** The diagnostic effect of CXCL16 in the test set. **(C)** The diagnostic effect of GPM6A in the test set. **(D)** The diagnostic effect of S100A9 in the test set. **(E)** The diagnostic effect of SCARA5 in the test set. The points marked on the ROC curve are the optimal threshold points, and the values in parentheses represent sensitivity and specificity. The AUC value is the area under the ROC curve.

In this study, the SVM-RFE technique was used to identify 22 genes as relevant biomarkers for DEGs (Figure 5A and Supplementary Table 5). Furthermore, the RF algorithm identified eight genes as significant biomarkers (Figure 5B and Supplementary Table 6). Five genes, including CXCL16, GPM6A, BEX2, S100A9, and SCARA5, overlapped across the two methods (Figure 5C), with two (CXCL16 and S100A9) being upregulated and three (GPM6A, BEX2, and SCARA5) being downregulated (Figure 5D). The validation set was used to determine the expression levels of the five biomarkers. Genes BEX2, GPM6A, and SCARA5 were considerably downregulated in AVC samples compared to normal samples (*P* < 0.05), whereas CXCL16 and S100A9 were significantly upregulated (Figures 6A–E), demonstrating that the findings were consistent and reliable. Additional analyses were conducted in the training and validation sets to establish the diagnostic effectiveness of the five markers. Table 2 for CXCL16, GPM6A, BEX2, S100A9, and SCARA5 in the merged dataset indicated that their probability of being useful biomarkers was 0.953, 0.897, 0.867, 0.897, and 0.887, respectively (Figures 7A–E), showing each biomarker had a high diagnostic value accuracy. In the GSE83453 dataset, Table 2 for CXCL16, GPM6A, BEX2, S100A9, and SCARA5 had probability of 0.861, 0.944, 0.861, 0.875, and 0.931, respectively, indicating that the five biomarkers had a high diagnostic accuracy (Figures 8A–E).

### Building an artificial neural network

**FIGURE 9:**
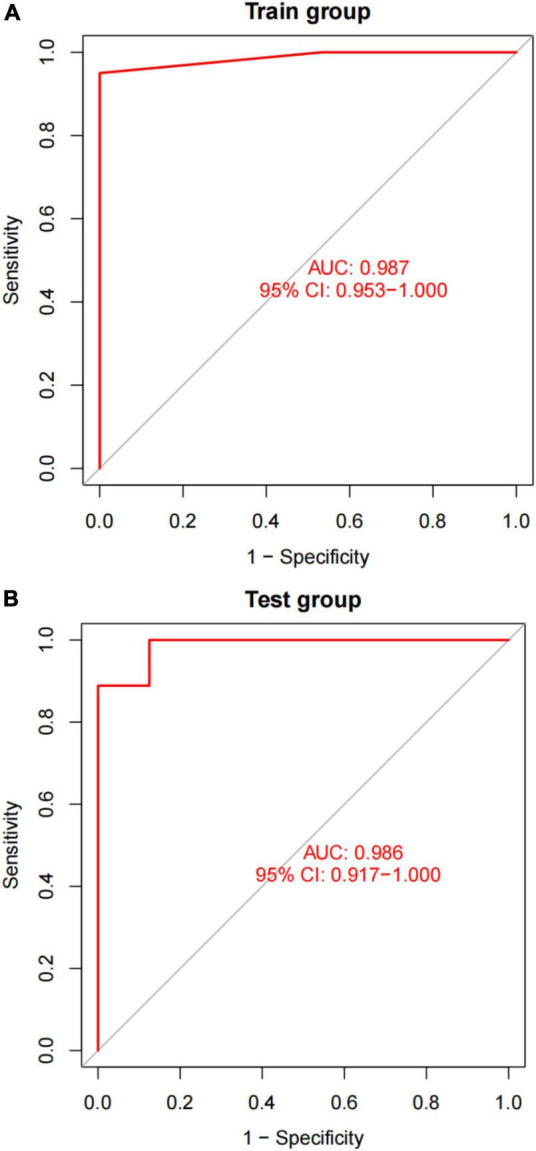
A plot of the results of AUC verification. **(A)** AUC verification in the training dataset. **(B)** AUC verification in the test dataset. The ROC curve illustrates the suitable threshold points, while the values in parenthesis represent the sensitivity and specificity. The AUC value represents the area under the ROC curve.

After converting the expression data of the five markers to Gene score (Supplementary Figures 2A,B and Table 3). The gene weights given to each gene are listed in Table 4. The gene score of all markers was multiplied by the gene weight, and the results were used to calculate diagnostic value (Table 5). Then, we set the diagnostic value of 35 samples as predicted values and set the diagnosis of AVC as true values. Diagnostic results are depicted in Table 6. Using the ROC package, the AUC of our model was evaluated to be 0.987 (95% confidence interval [CI]: 0.953–1.000), suggesting that the model has a strong diagnostic capability (Figure 9A).

**TABLE 3:** Results of gene scores for all samples of the combined dataset of GSE12644 and GSE51472.

Samples	CXCL16	S100A9	GPM6A	BEX2	SCARA5
GSM317342_Normal	0	1	0	0	0
GSM317343_Normal	0	0	0	0	0
GSM317344_Normal	0	0	0	0	0
GSM317345_Normal	0	0	0	0	0
GSM317346_Normal	0	0	0	0	0
GSM377368_Normal	0	0	0	0	0
GSM377369_Normal	1	0	0	0	0
GSM377370_Normal	0	0	0	0	0
GSM377371_Normal	0	0	0	0	1
GSM377372_Normal	0	0	0	0	1
GSM1246204_Normal	0	0	1	0	0
GSM1246205_Normal	0	0	0	0	0
GSM1246206_Normal	0	0	0	1	0
GSM1246207_Normal	0	0	0	0	0
GSM1246208_Normal	0	0	1	0	0
GSM317347_AVC	1	1	1	1	0
GSM317348_AVC	1	1	1	1	1
GSM317349_AVC	1	1	1	1	1
GSM317350_AVC	1	1	1	1	1
GSM317351_AVC	1	1	1	1	1
GSM377373_AVC	1	1	0	1	1
GSM377374_AVC	1	1	1	1	1
GSM377375_AVC	1	1	0	1	1
GSM377376_AVC	1	1	1	1	1
GSM377377_AVC	1	0	1	1	1
GSM1246209_AVC	0	0	1	1	0
GSM1246210_AVC	0	0	0	1	1
GSM1246211_AVC	0	0	0	0	0
GSM1246212_AVC	0	1	1	1	0
GSM1246213_AVC	1	1	1	1	1
GSM1246214_AVC	1	1	1	0	1
GSM1246215_AVC	1	1	1	0	1
GSM1246216_AVC	1	1	1	1	1
GSM1246217_AVC	1	1	1	1	1
GSM1246218_AVC	1	1	1	1	1

AVC, aortic valve calcification.

**TABLE 4:** Gene weights of five markers for all samples of the combined dataset of GSE12644 and GSE51472.

	The weight of genes in the hidden layer					The weight of genes in the output layer	
		
	Hidden	Hidden	Hidden	Hidden	Hidden	Output layer 1	Output layer 2
	layer 1	layer 2	layer 3	layer 4	layer 5		
CXCL16	1.4422024	0.8067851	–1.2517474	0.81237475	–1.06361	0.1194611	–0.82155472
S100A9	–0.1110034	–0.4236217	0.4201573	–0.02445162	1.550808	0.2818231	0.29068938
GPM6A	–0.3588348	2.3986261	0.848421	–1.39610883	10.063427	1.0300879	–0.63177691
BEX2	1.0788502	0.6817263	–0.4659392	0.52948785	9.040834	0.5961824	0.66712552
SCARA5	–1.0053437	–0.3655945	1.1181273	–0.65628306	9.616735	–1.1183066	1.10673166

**TABLE 5:** Diagnostic value for all samples of the combined dataset of GSE12644 and GSE51472.

Samples	Diagnostic value of	Diagnostic value
	normal samples	of AVC samples
GSM317342_Normal	0.94618326	0.056753254
GSM317343_Normal	0.904296846	0.097606317
GSM317344_Normal	0.904296846	0.097606317
GSM317345_Normal	0.904296846	0.097606317
GSM317346_Normal	0.904296846	0.097606317
GSM377368_Normal	0.904296846	0.097606317
GSM377369_Normal	0.973335408	0.029453828
GSM377370_Normal	0.904296846	0.097606317
GSM377371_Normal	0.982576352	0.001961258
GSM377372_Normal	0.982576352	0.001961258
GSM1246204_Normal	0.993381143	0.002593156
GSM1246205_Normal	0.904296846	0.097606317
GSM1246206_Normal	0.98247582	0.024367009
GSM1246207_Normal	0.904296846	0.097606317
GSM1246208_Normal	0.993381143	0.002593156
GSM317347_AVC	0.019810466	1.001116247
GSM317348_AVC	0.004836848	0.995356568
GSM317349_AVC	0.004836848	0.995356568
GSM317350_AVC	0.004836848	0.995356568
GSM317351_AVC	0.004836848	0.995356568
GSM377373_AVC	–0.007721178	0.994428405
GSM377374_AVC	0.004836848	0.995356568
GSM377375_AVC	–0.007721178	0.994428405
GSM377376_AVC	0.004836848	0.995356568
GSM377377_AVC	–0.050916168	1.037641252
GSM1246209_AVC	–0.001847649	0.995498191
GSM1246210_AVC	0.010441263	1.007113243
GSM1246211_AVC	0.904296846	0.097606317
GSM1246212_AVC	–0.004724825	1.005070695
GSM1246213_AVC	0.004836848	0.995356568
GSM1246214_AVC	–0.003228033	1.003823674
GSM1246215_AVC	–0.003228033	1.003823674
GSM1246216_AVC	0.004836848	0.995356568
GSM1246217_AVC	0.004836848	0.995356568
GSM1246218_AVC	0.004836848	0.995356568

AVC, aortic valve calcification.

**TABLE 6:** Diagnostic accuracy for all samples of the combined dataset of GSE12644 and GSE51472.

Group	Normal	AVC	Diagnostic accuracy
Normal	15	0	1
AVC	1	19	0.95

AVC, aortic valve calcification.

### Validation of diagnostic models

A different dataset (GSE83453) was used to test the capacity of the model to detect AVC. Furthermore, we used five markers from the screened validation set that were identical to those in the training set, proving the scalability and resilience of the scientific algorithm. GSE83453 dataset was then used in the same method as the diagnostic value with serving as the test set. Gene score was described in Table 3. The gene weights were described in Table 4. Diagnostic value was described in Table 5; Diagnostic results are depicted in Table 6. The AUC for the validation model was 0.986 (95% CI: 0.917–1.000), demonstrating the accuracy and durability of the model (Figure 9B).

### Immune cell infiltration results

**FIGURE 10:**
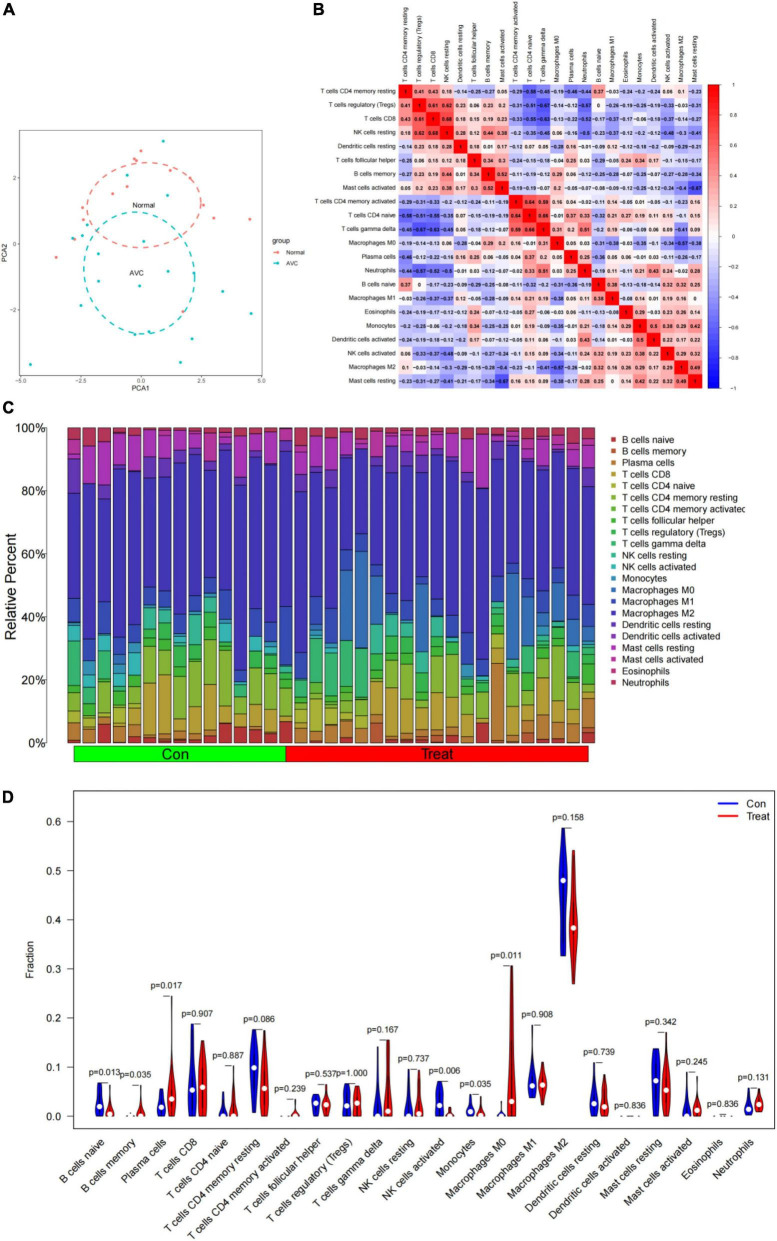
Immune cell infiltrates are evaluated and illustrated. **(A)** Cluster plot of immune cell infiltration between AVC and normal samples using PCA. **(B)** Heatmap demonstrating correlations between 22 distinct types of immune cells. The size of the colored squares indicates the connection’s strength; red indicates a positive correlation, while blue indicates a negative correlation. A darker hue indicates a stronger connection. **(C)** Relative percentages of 22 immune cell subpopulations in 35 samples from the GSE12644 and GSE51472 datasets. **(D)** A violin diagram illustrating the relative proportions of 22 different kinds of immune cells. The red marks depict the infiltration difference between the two sets of samples.

CIBERSORT revealed distinctions in 22 subpopulations of infiltrating immune cells between 15 normal and 20 AVC samples (Supplementary Table 7). The PCA revealed differential group-bias clustering and individual differences in proportions of immune cells in AVC and normal samples (Figure 10A and Supplementary Table 8). The proportion of 22 immune cells in normal and AVC samples was readily apparent (Figure 10C), and we discovered that macrophages constituted the majority of the immune cells. The outcomes of the immune cell infiltration are illustrated in Supplementary Table 5. The correlation results among immune cells are shown in Figure 10B. The patterns of changes in the various immune cell showed that AVC patients had higher infiltration of macrophages M0 (*P* = 0.011), memory B cells (*P* = 0.035), and plasma cells (*P* = 0.017) than normal samples, but B cells naive (*P* = 0.013), NK cells activated (*P* = 0.006), and monocytes (*P* = 0.035) were lower (Figure 10D).

### Correlation analysis of five markers with infiltration immune cells

**FIGURE 11:**
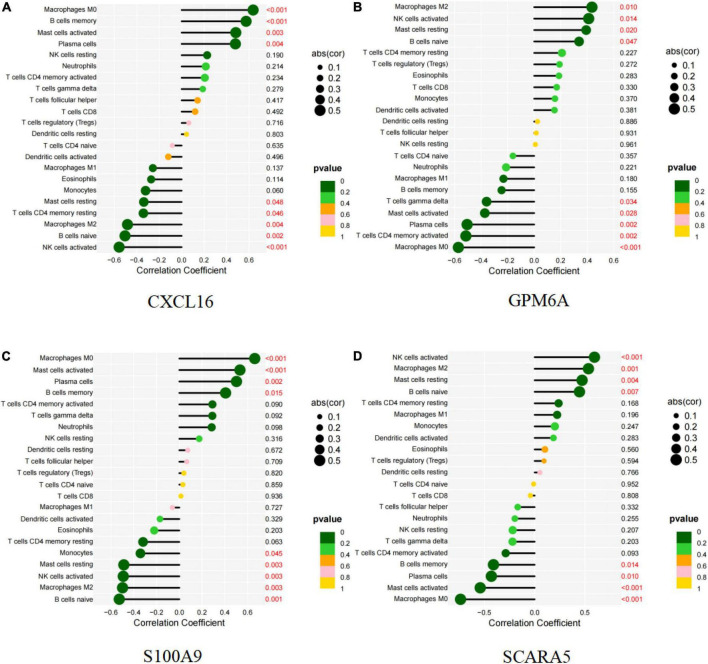
Correlation between diagnostic markers and infiltrating immune cells. **(A)** Correlation between CXCL16 and infiltrating immune cells. **(B)** Correlation between GPM6A and infiltrating immune cells. **(C)** Correlation between S100A9 and infiltrating immune cells. **(D)** Correlation between SCARA5 and infiltrating immune cells. The size of the dots indicates the degree to which genes and immune cells are correlated. Correlation strength is proportional to the size of the dots. The color of the dots indicates the *p*-value; a yellower hue indicates a lower *p*-value, while a greener color indicates a higher *p*-value. *p*-value < 0.05 was considered statistically significant.

CXCL16 and S100A9 were positively correlated with macrophages M0, mast cells activated, plasma cells, B cells memory, but negatively correlated with mast cells resting (*P* < 0.05; Figures 11A,C and Supplementary Figures 3A–I, 5A–I). GPM6A and SCARA5 were positively correlated with macrophages M2, NK activated cells, mast resting cells, and B naive cells, but negatively correlated with mast activated cells, plasma cells and macrophages M0 (*P* < 0.05; Figures 11B,D and Supplementary Figures 4A–I, 6A–H). Interestingly, BEX2 had no significant correlation with immune cells. The above results are shown in Supplementary Table 9.

### Small molecule drug screening

**FIGURE 12:**
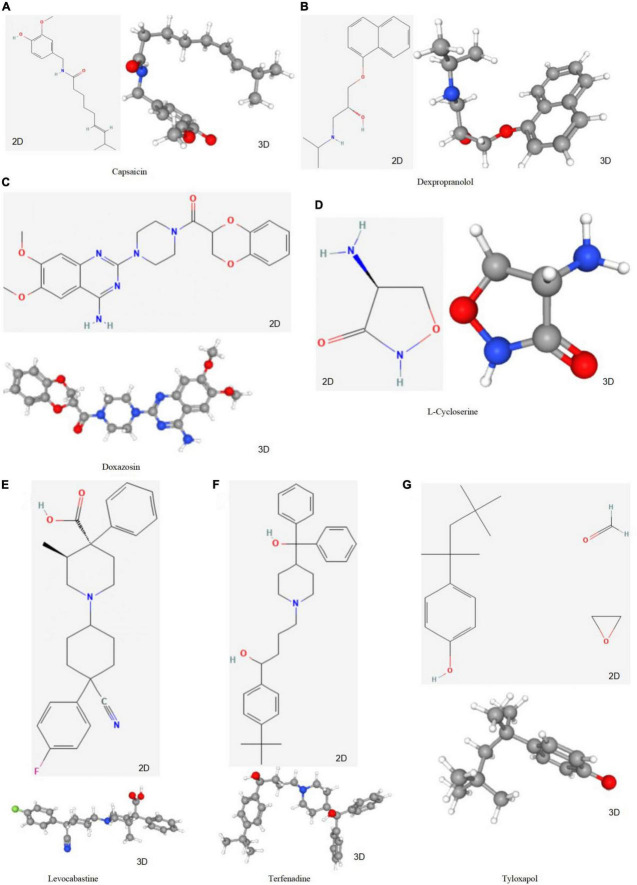
The structure of small molecule drugs screened based on CMap. **(A)** Capsaicin’s 2D and 3D structure. **(B)** Dexpropranolol’s 2D and 3D structure. **(C)** Doxazosin’s 2D and 3D structure. **(D)** L-Cycloserine’s 2D and 3D structure. **(E)** Levocabastine’s 2D and 3D structure. **(F)** Terfenadine’s 2D and 3D structure. **(G)** Tyloxapol’s 2D and 3D structure.

To screen for prospective small molecule medicines that target AVC gene expression, all diagnostic markers were classified as upregulated or downregulated and submitted to the CMap database. The top ten most significant small molecule medications were identified (Table 7). Doxazosin (enrichment score = –0.873) and Terfenadine (enrichment score = –0.809) had a strong negative connection and may reverse the AVC condition. The newly identified small molecule medicines with an enrichment score of 0 were capable of reversing abnormal gene expression and may be used to treat AVC. Doxazosin, Terfenadine, Levcycloserine, Tyloxapol, Dexpropranolol, and Levocabastine all had matching molecular structures in Figures 12A–G, whereas, Prestwick-1084, STOCK1N-28457, and SC-560 did not.

**TABLE 7:** The top 10 small molecule drug screening based on CMap (the smallest enrichment score).

Drugs	Mean	Enrichment	*P*
Doxazosin	–0.811	–0.873	0.00056
Terfenadine	–0.809	–0.858	0.00563
Prestwick-1084	–0.821	–0.843	0.00107
STOCK1N-28457	–0.785	–0.812	0.01326
Levcycloserine	–0.792	–0.799	0.0032
Tyloxapol	–0.845	–0.791	0.00392
SC-560	–0.718	–0.754	0.03031
Dexpropranolol	–0.779	–0.751	0.03165
Levocabastine	–0.741	–0.742	0.00875
Capsaicin	–0.767	–0.741	0.00891

## Discussion

Calcific aortic valve disease is the most prevalent valve disease in developed countries, and its incidence is likely to increase further in the future decades as life expectancy continues to rise (8). Due to this common and insidious condition, the progressive increase in the degree of AVC leads to a large proportion of patients being unsuitable for surgical treatment (37). Consequently, there has been a surge in interest in the development of novel pharmacological targets or treatment techniques that may be used early in the early stages of the disease. Therefore, identifying specific therapeutic targets and investigating immune cell infiltration patterns associated with AVC are critical for improving the prognosis of AVC patients.

Earlier studies on AVC patients reported that five genes (*CXCL16, GPM6A, BEX2, S100A9*, and *SCARA5*) are potential biomarkers of AVC, similar to the present findings (38). To date, however, comprehensive, strong, and reliable clinical decision-making approaches are lacking. Machine learning has emerged as a useful tool in bioinformatics for sifting through mountains of data to find valuable information. Machine learning algorithms analyze training data in order to discover hidden patterns, create models, and then make predictions based on the most accurate of these. In reality, popular techniques (such SVM and RF) have been applied to problems in genomics, proteomics, systems biology, and other areas (39). Conventional methods of machine learning rely heavily on data representations known as features for maximum performance. However, it remains challenging to determine which features are better suitable for a particular project throughout the application process. Deep learning is a relatively new subfield of machine learning based on enormous amounts of data, the capacity of parallel and distributed computers, and complex algorithms. In addition, deep learning is responsible for substantial breakthroughs in other fields where artificial intelligence has struggled for decades (40). In bioinformatics research, splice junctions may be identified from DNA sequences, finger joints can be identified from X-ray images, and lapses can be identified from electroencephalography data, among other notable advancements. In their respective reviews, Yu et al. (41) and Mamoshina et al. (42) emphasize the successful application of deep learning in bioinformatics research. In bioinformatics, where traditional machine learning has made significant strides, it is therefore anticipated that deep learning would produce positive outcomes. In this study, we take advantage of machine learning and artificial neural networks to not only improve the statistical strength of our diagnostic model, but also transfer the theoretical diagnostic genome for application in common clinical practice. The random forest model (RF), a non-parametric technique used for classification under supervision (43), covers respective decision trees originating from subdivided data sets. In the current study, we trained and analyzed a single RF classification model in order to discover descriptors capable of discriminating AVC from a general sample. SVM-RFE is a machine learning technique that is widely applied to not only rank features but also select the most important ones for classification (44). On the other hand, ANN has several advantages, including significant defect and failure tolerance, scalability, and the capacity for consistent generalization, all which contribute to the model’s stability and reliability (45).

Here, we adopted two separate algorithms, each with its own set of intrinsic properties. Finally, we identified important genes and subsequently constructed diagnostic models with precise outcomes. Overall, our diagnostics revealed that the integration method is feasible. Moreover, previous studies have shown that the scoring method is simple, cost-efficient, and very successful in identifying heterogeneity across disease subtypes (33). Particularly, it converts complicated gene expression data to simple clinical scores, thereby enabling physicians to further screen for aortic valve calcification during physical exams of patients. This consequently allows for both early detection and treatment, thereby effectively delaying disease progression. A previous study showed that CXCL16 binds to oxidized low-density lipoprotein, suggesting it’s linked to atherosclerosis (46). Additionally, CXCL16 may influence AVC development by generating a strong chemotactic response and calcium mobilization (47). A recent study showed that catechin intake not only mediated a significant reduction in the extent of atherosclerotic lesions but also downregulated levels of SCARA5 expression (48). Zhao et al. (49) showed that platelet-derived growth factor increased SCARA5 in human aortic smooth muscle cells, suggesting it may be crucial during atherosclerosis progression. Additionally, downregulating SCARA5 reduced aortic lipid buildup, consistent with a previous study (50). In the present study, SCARA5 was significantly down-regulated in normal tissues, indicating that this gene plays a similar function in the AVC process. S100A9, a candidate for human cardiovascular risk indicators (51), promotes atherosclerosis in a mouse model (52). This suggests that S100A9 may be both a biomarker for and regulator of atherosclerosis. S100A9 levels are raised in autoimmune and pro-inflammatory diseases including rheumatoid arthritis (53) and obesity (54), which increases the risk of cardiovascular disease. S100A9-deficient animals display pro-inflammatory characteristics in sepsis (55) and pancreatitis (56). Since S100A9 is involved in regulation of vascular inflammation, early targeting of this factor may be a potential therapy for treatment of different types of vascular damage. S100A9 levels in plasma are also used to predict cardiovascular disease risk and to detect acute cardiovascular events, such as atherosclerotic plaque rupture and thrombosis (51, 57, 58). In summary, CXCL16, S100A9 and SCARA5 may influence AVC progression and serve as diagnostic markers. However, the mechanisms of GPM6A and BEX2 in the cardiovascular domain remain unclear. Therefore, the diagnostic significance of GPM6A and BEX2 needs to be further validated by numerous studies.

Previous research evidences have suggested that the pathological processes of AVC and atherosclerosis are similar (7). Particularly, numerous studies have shown that AVC is characterized by an inflammatory response (59). This is consistent with our pathway analysis results from GO, KEGG, and metascape. Several studies have reported an amount of B cells inside severe aortic valve stenosis patient’s valves, implying that increasing B cell counts may exacerbate aortic valve failure (60–62). Interestingly, this may occur even in cases without clinically severe atherosclerosis and B cell buildup inside the aortic valve and their interaction with macrophages may contribute to the aortic valve’s gradual thickening and calcification. Therefore, depletion of mature B cells has shown promise as a potential therapeutic modality. Furthermore, Kaden et al. (63) demonstrated that very few macrophages accumulated in normal human aorta, but excised calcified human aortic valves contained a dense infiltration of both leukocytes and macrophages. Results of the present study revealed significant elevation of macrophage M0 in the calcified valves, which was consistent with the findings of a previous study (64). Additional research evidences have shown that the quantity of fibrin and the degree of valve calcification are correlated with the number of alternatively activated macrophages recruited to the valve leaflets (65). In fact, macrophages are not only responsible for directing pro-inflammatory immune response, but their numbers are also elevated in AVC. Functionally, macrophages release interleukin 6 (IL-6) and tumor necrosis factor alpha (TNF-α) as part of this pro-inflammatory response, both which induce calcification of aortic valve interstitial cells (66). Additionally, previous studies have shown that the mechanical strain encountered by the valve during circulation increases both macrophage activation and inflammation, implying that mechanical tension may promote the two processes in the valve (67). Further studies show evidence of lymphocyte, phagocytic, histiocyte, and mast cell infiltration in calcific aortic stenosis (61). On the other hand, NK cell aggregation in the valve and circulation have been detected in AVC patients, with this phenomenon closely associated with increased pressure gradient in the valve (68). Results from our correlation analysis demonstrated the relationship between immune cells and diagnostic markers. Particularly, CXCL16 and S100A9 expression had significant positive correlation with Macrophages M0, B memory cells, Mast activated cells and Plasma cells. Collectively, multiple studies have reported the ability of CXCL16 (69–72) and S100A9 (73, 74) stimulate the infiltration of macrophages in atherosclerosis, promoting disease progression, suggesting that they also may be playing a synergistic role in promoting AVC progression.

We identified a series of small-molecule drugs that may prevent AVC progression. Doxazosin and Terfenadine demonstrated a negative connection and may treat AVC. Doxazosin, an ana1-adrenergic antihypertensive medication, successfully lowers blood pressure, plasma cholesterol, triglyceride levels and density lipoprotein, while raising high–density lipoprotein (75–78). Decreased oxidized low-density lipoprotein cholesterol may not fully explain Doxazosin’s anti-atherogenic effect, but it may be one of its supplementary weapons for avoiding atherosclerosis in addition to blood pressure and lipid reduction. Additionally, terfenadine is an anti-allergic H1 receptor antagonist that is highly selective and specific. The medicine prevents allergic conjunctivitis, cutaneous, nasal, and bronchial reactions (79). Additionally, Terfenadine decreases histamine’s H1 activity, inflammatory infiltration (eosinophils, neutrophils), and mast cell mediator release (80). Therefore, it also contributes to the delay in the progression of AVC. With the availability of these small molecule drugs, additional research into their possible effects on AVC is critical and will aid in the development of novel AVC treatment therapies.

The present research has several limitations. To begin, despite the effectiveness of our diagnostic model on both the training and validation datasets, the sample size used to construct and test the diagnostic model was modest. Second, a GEO dataset was used to validate the model. Additional experimental validation should be performed to verify the biomarkers’ expression. Due to the difficulty of obtaining normal aortic valve samples, this study cannot be effectively verified by experiments. We will continue to collect samples for further research. Thirdly, the approach we developed is limited to the detection of AVC. Additional validation is required to see whether the model can be used to diagnose AVC patients. Finally, the lack of clinical information on gender in this analysis may lead to biased results, and subsequent clinical validation should be performed using gender-based grouping.

## Conclusion

We developed a diagnostic model for the detection of aortic valve calcification in patients using machine learning and ANN model. The diagnostic model was validated using an independent cohort drawn from the GEO database. Our findings present doctors with a novel treatment strategy that may help them in making more informed therapeutic decisions and potentially delay the progression of AVC. The predicted genes and pathways identified in this model should be further investigated to gain a better understanding of the biological processes influencing patient response to AVC. Finally, small molecule drugs capable of reversing the AVC state such as Doxazosin and Terfenadine were identified.

## Data availability statement

The datasets presented in this study can be found in online repositories. The names of the repository/repositories and accession number(s) can be found in the article/Supplementary material.

## Ethics statement

Ethical review and approval was not required for the study on human participants in accordance with the local legislation and institutional requirements. Written informed consent for participation was not required for this study in accordance with the national legislation and the institutional requirements. Written informed consent was not obtained from the individual(s) for the publication of any potentially identifiable images or data included in this article.

## Author contributions

TX was responsible for study conception. YC and SH were responsible for methodology. TX was responsible for checking code. Y-XF, LP, and W-CF were responsible for software. Y-YZ and YC were responsible for writing the original draft. Y-XL was responsible for review and editing. All authors reviewed and approved the final version of the work.

## Footnotes

1
www.nci.nih.gov/geo

2
http://metascape.org

3
https://clue.io/

## Supplementary material

Supplementary Figure 1 Principal component analysis (PCA) analysis of gene expression matrix. **(A)** Before removing the batch effects. **(B)** After removing the batch effect. The scatter plots depict samples using the top two principal components (PC1 and PC2) of gene expression profiles. The colors correspond to samples from two distinct datasets.

Supplementary Figure 2 Results of neural network visualization. **(A)** Visualization of the neural network process. **(B)** Visualization of gene weight in a neural network.

Supplementary Figure 3 Visualization of correlation between markers and immune cells. **(A–I)** CXCL16 is positively correlated with Macrophages M0 (*P* < 0.001), B memory cells (*P* < 0.001), Mast activated cells (*P* = 0.003) and Plasma cells (*P* = 0.004), as well as negatively correlated with Mast cells resting (*P* = 0.048), T cells CD4 memory resting (*P* = 0.046), Macrophages M2 (*P* = 0.004), B cells naive (*P* = 0.002), and NK cells activated (*P* < 0.001).

Supplementary Figure 4 **(A–I)** GPM6A is positively correlated with Macrophages M2 (*P* = 0.010), NK activated cells (*P* = 0.014), Mast resting cells (*P* = 0.020), and B naive cells (*P* = 0.047), as well as negatively correlated with T cells gamma delta (*P* = 0.034), Mast activated cells (*P* = 0.028), Plasma cells (*P* = 0.002), CD4 memory activated T Cells (*P* = 0.002), and Macrophages M0 (*P* < 0.001).

Supplementary Figure 5 **(A–I)** S100A9 is positively correlated with Macrophages M0 (*P* < 0.001), Mast cells activated (*P* < 0.001), Plasma cells (*P* = 0.002), B cells memory (*P* = 0.015), as well as is negatively correlated with Monocytes (*P* = 0.045), Mast cells resting (*P* = 0.003), NK cells activated (*P* = 0.003), Macrophages M2 (*P* = 0.003), and B cells naive (P = 0.001).

Supplementary Figure 6 **(A–H)** SCARA5 is positively correlated with NK cells activated (*P* < 0.001), Macrophages M2 (*P* = 0.001), Mast cells resting (*P* = 0.004), and B cells naive (*P* = 0.007), as well as negatively correlated with B cells memory (*P* = 0.014), Plasma cells (*P* = 0.010), Mast cells activated (*P* < 0.001), and Macrophages M0 (*P* < 0.001).

## Abbreviations

AVC: aortic valve calcification
DEGs: differentially expressed genes
ROC: receiver operating characteristic
CXCL16: C-X-C chemokine ligand 16
GPM6A: glycoprotein M6A
BEX2: brain expressed X-linked 2
S100A9: S100 calcium-binding protein A9
SCARA5: scavenger receptor class A member 5
GO: gene ontology
KEGG: kyoto encyclopedia of genes and genomes
PCA: principal component analysis
ANN: artificial neural networks
RF: random forest
SVM-RFE: support vector machine recursive feature elimination
AUC: area under the curve
BP: biological process
CC: cellular component
MF: molecular function
IL: interleukin
TNF: tumor necrosis factor.
