# Supplementary material for: Development and analysis of a comprehensive diagnostic model for aortic valve calcification using machine learning methods and artificial neural networks
Source: Front Cardiovasc Med. 2022 Dec 1;9:913776. doi: 10.3389/fcvm.2022.913776 (PMC9751025; doi:10.3389/fcvm.2022.913776)
Supplement: Supplementary Figure 1 — Principal component analysis (PCA) analysis of gene expression matrix. (A) Before removing the batch effects. (B) After removing the batch effect. The scatter plots depict samples using the top two principal components (PC1 and PC2) of gene expression profiles. The colors correspond to samples from two distinct datasets. [file Data_Sheet_1.docx]

**Supplementary Figure 1**


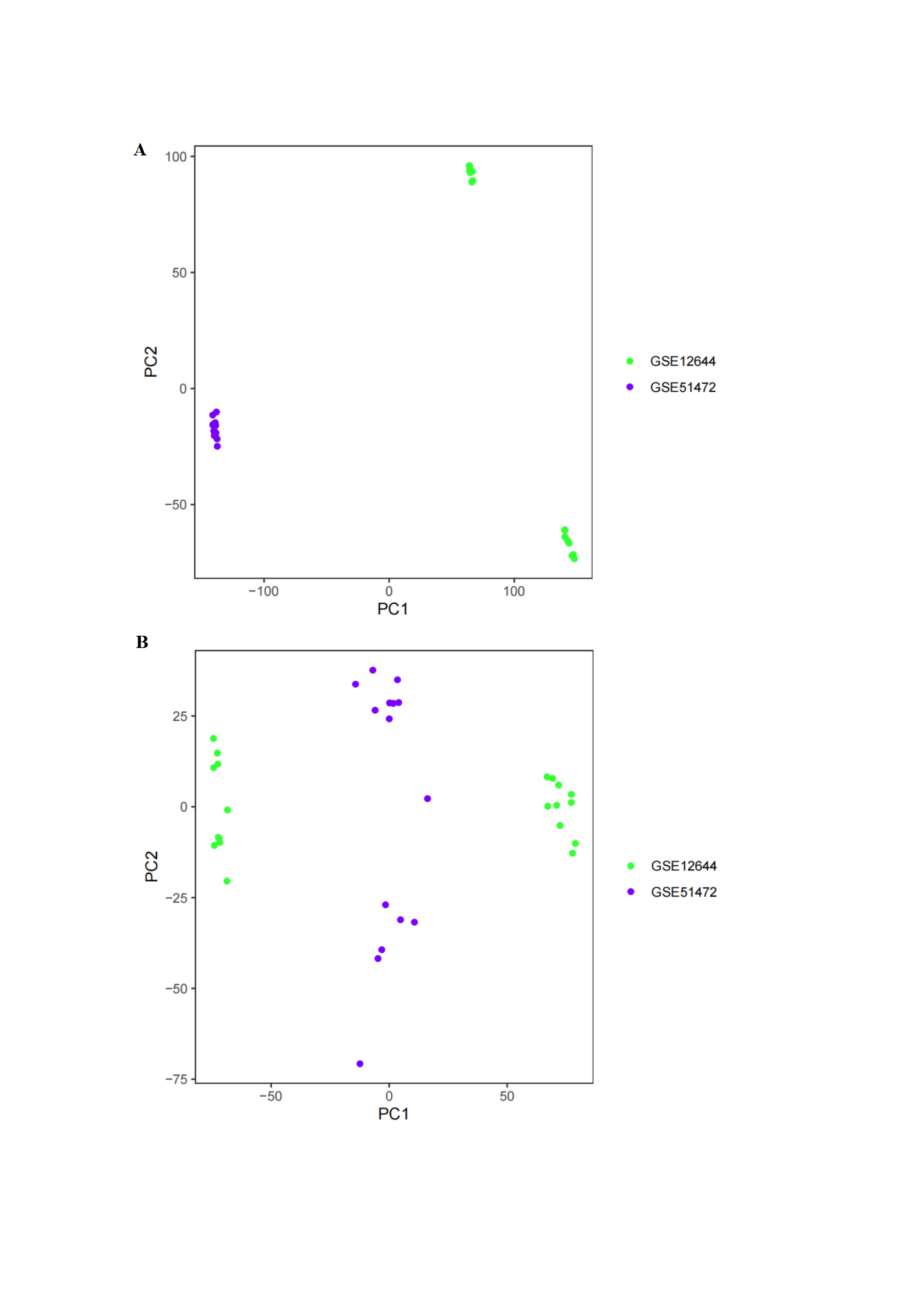


**Supplementary Figure 2**

**
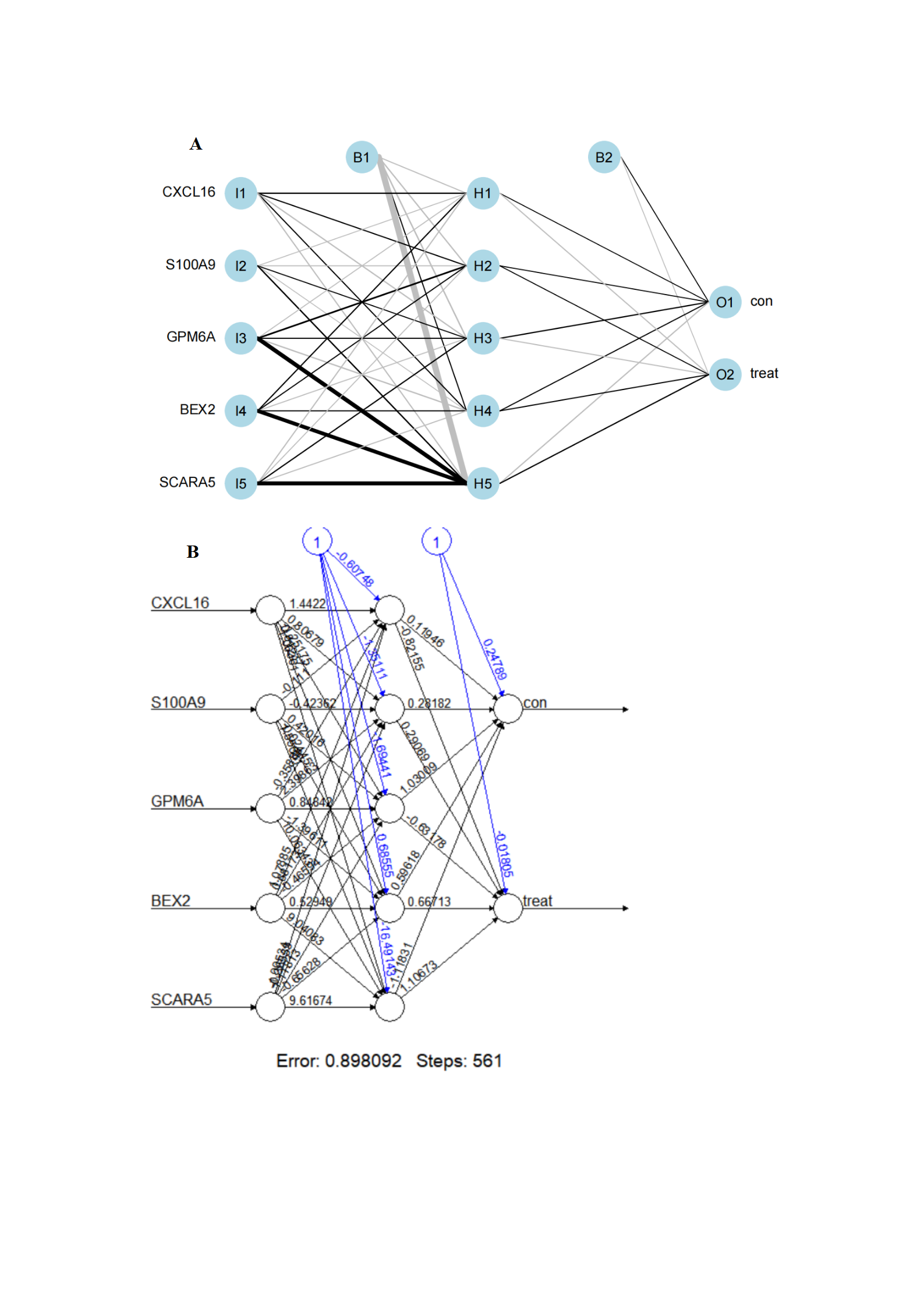
**

**Supplementary Figure 3**

**
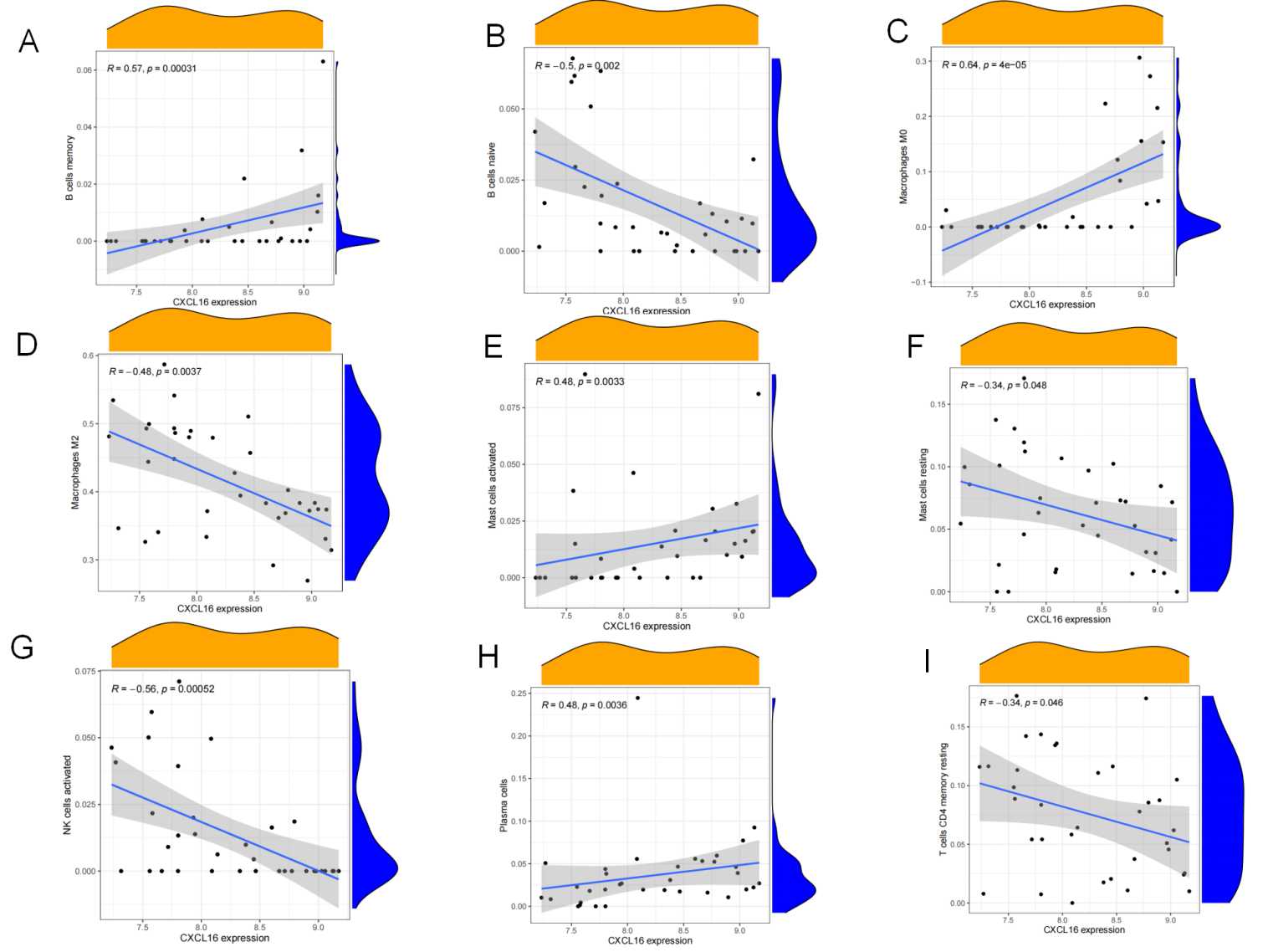
**

**Supplementary Figure 4**

**
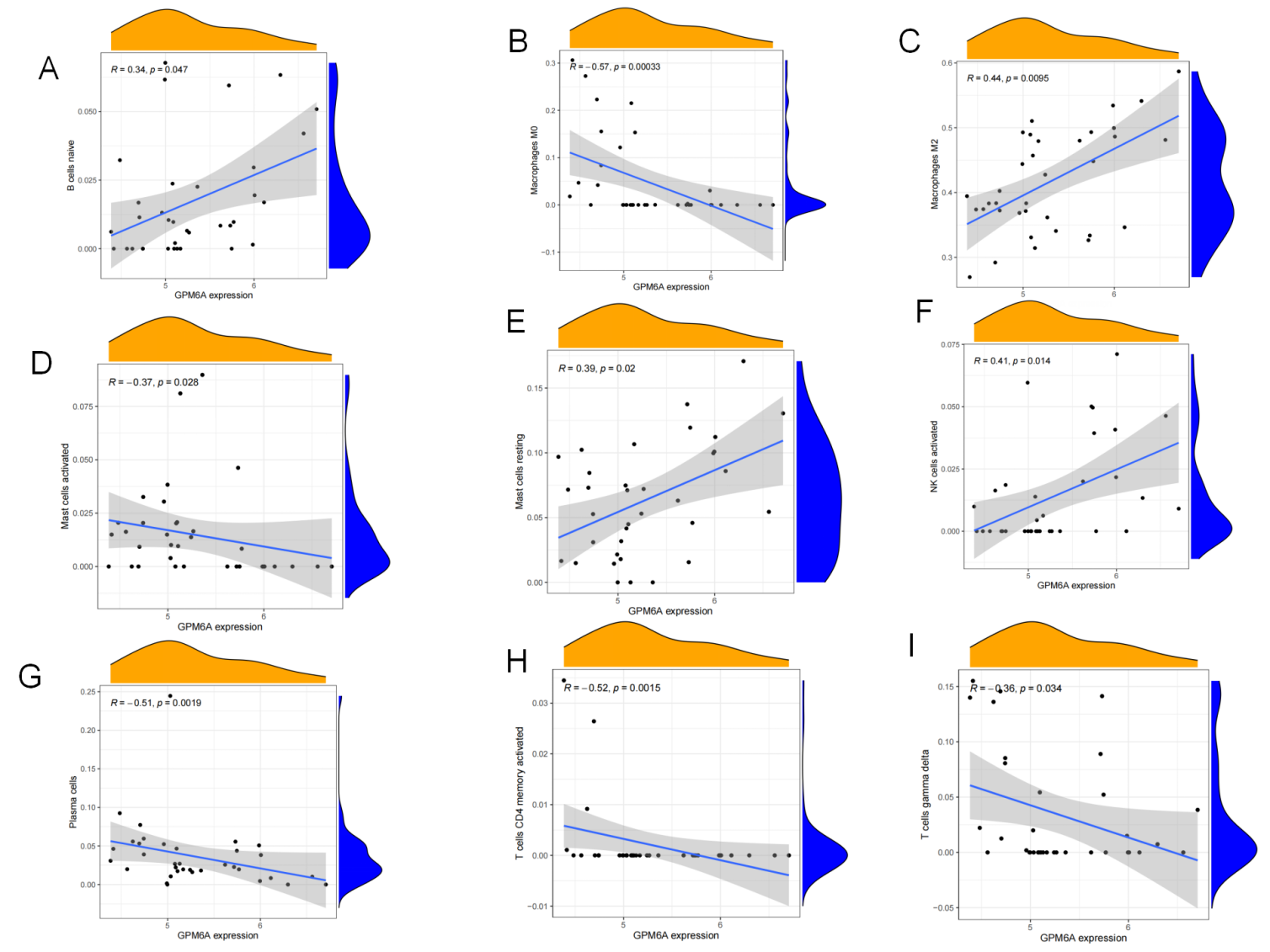
**

**Supplementary Figure 5**

**
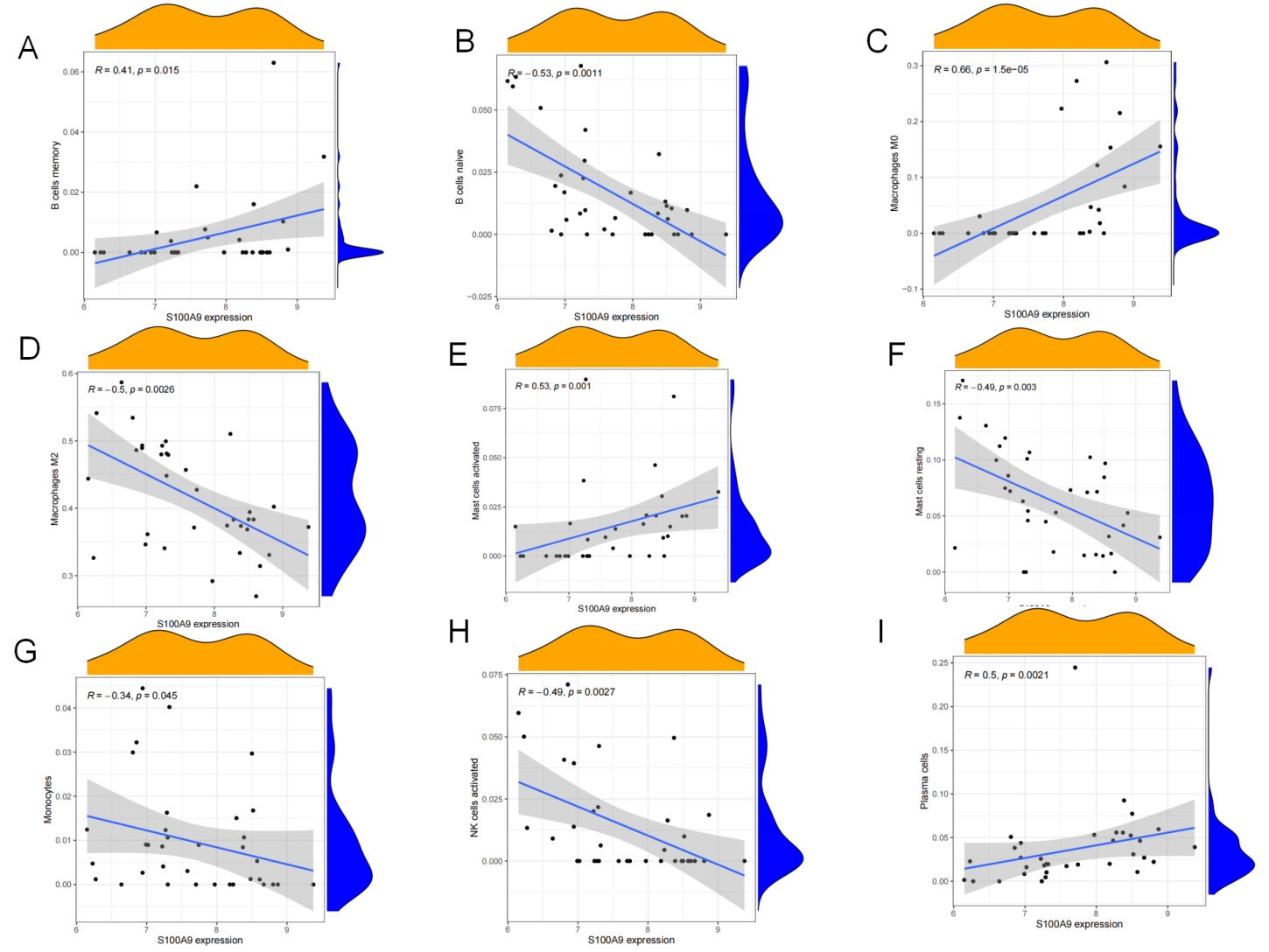
**

**Supplementary Figure 6**

**
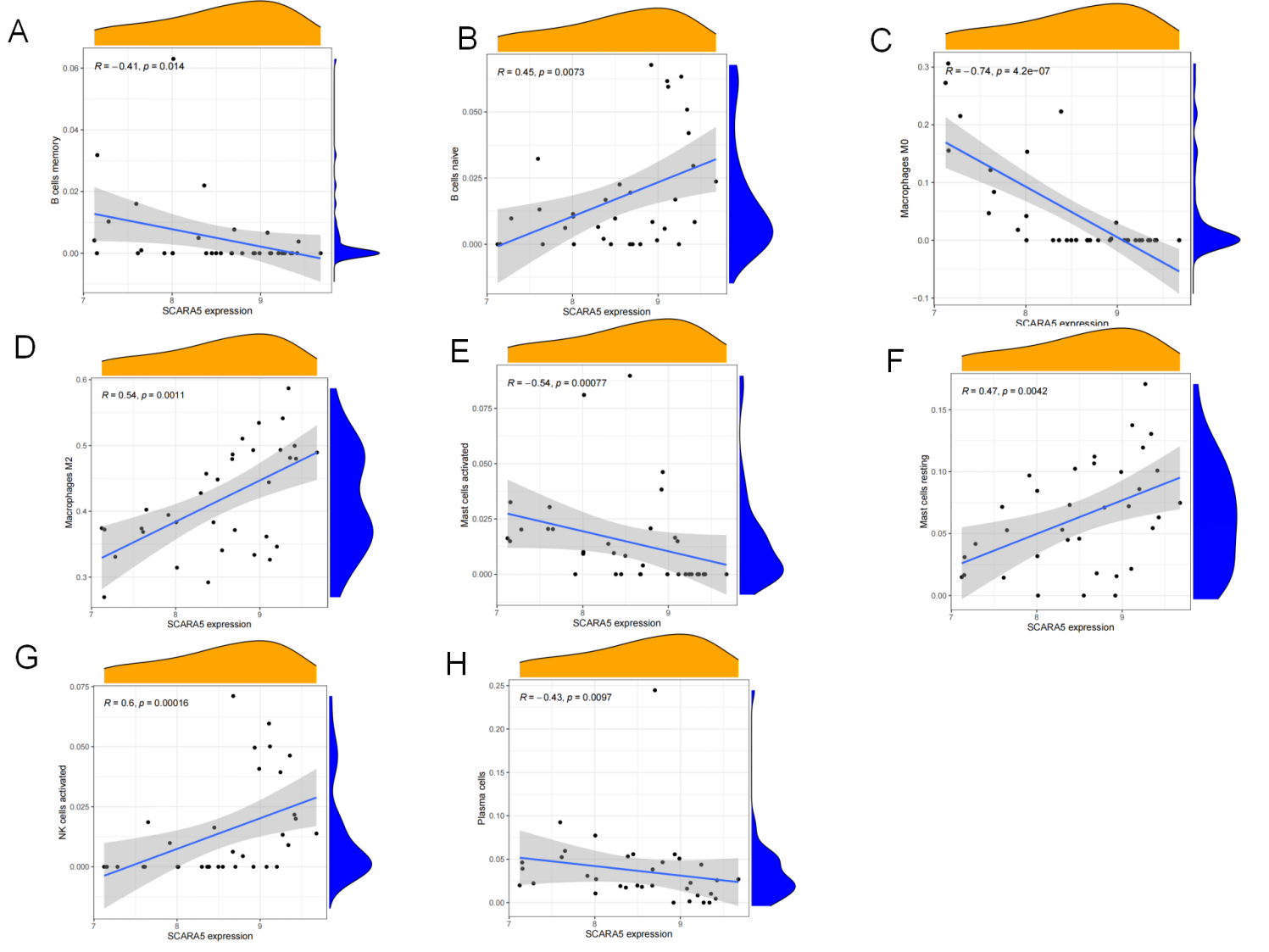
**
