# Supplementary material for: Development and analysis of a comprehensive diagnostic model for aortic valve calcification using machine learning methods and artificial neural networks
Source: Front Cardiovasc Med. 2022 Dec 1;9:913776. doi: 10.3389/fcvm.2022.913776 (PMC9751025; doi:10.3389/fcvm.2022.913776)
Supplement: Supplementary file 2 [file Table_1.docx]

SUPPLEMENTARY TABLE 1: Differentially expressed genes (DEGs) of merged data sets of GSE12644 and GSE51472.

| Genes | logFC | AveExpr | t | P.Value | adj.P.Val | B |
| --- | --- | --- | --- | --- | --- | --- |
| CXCL16 | 0.932489 | 8.240013 | 6.95665 | 4.16E-08 | 0.000814 | 8.254117 |
| SCG2 | 1.81596 | 8.675712 | 6.7338 | 8.12E-08 | 0.000814 | 7.665266 |
| PLTP | 0.941455 | 9.946273 | 6.624586 | 1.13E-07 | 0.000814 | 7.374756 |
| FN1 | 0.59873 | 11.2464 | 6.255526 | 3.44E-07 | 0.001864 | 6.384861 |
| FHL2 | 0.764978 | 9.754029 | 5.814374 | 1.32E-06 | 0.005311 | 5.188648 |
| LUM | 0.601065 | 10.95055 | 5.751063 | 1.60E-06 | 0.005311 | 5.016175 |
| CXCL12 | 0.764226 | 10.71891 | 5.671231 | 2.04E-06 | 0.005311 | 4.798505 |
| ALDH2 | -0.671725 | 10.3654 | -5.591058 | 2.60E-06 | 0.005311 | 4.579737 |
| ITM2A | -0.89929 | 9.198635 | -5.558191 | 2.88E-06 | 0.005311 | 4.490015 |
| GPM6A | -0.841819 | 5.289848 | -5.55098 | 2.94E-06 | 0.005311 | 4.470328 |
| PLAUR | 0.754865 | 7.308186 | 5.479034 | 3.67E-06 | 0.00546 | 4.273869 |
| PPBP | 1.956678 | 5.484217 | 5.277624 | 6.77E-06 | 0.008145 | 3.723826 |
| STEAP1 | 0.942237 | 8.539571 | 5.180794 | 9.09E-06 | 0.009371 | 3.459553 |
| S100A9 | 1.110267 | 7.675472 | 5.173801 | 9.29E-06 | 0.009371 | 3.440476 |
| CD93 | 1.010911 | 8.845816 | 5.167636 | 9.46E-06 | 0.009371 | 3.423657 |
| COL4A4 | -0.68407 | 6.003596 | -5.127775 | 1.07E-05 | 0.009597 | 3.314948 |
| SLAMF8 | 0.785216 | 7.322414 | 5.044989 | 1.37E-05 | 0.010674 | 3.089355 |
| CAB39L | -0.639477 | 7.596108 | -5.041342 | 1.39E-05 | 0.010674 | 3.079423 |
| ATP1A2 | -1.209701 | 6.722795 | -5.031713 | 1.43E-05 | 0.010674 | 3.053204 |
| SCARA5 | -0.950419 | 8.513504 | -5.000556 | 1.57E-05 | 0.011107 | 2.968394 |
| TNC | 1.0927 | 8.059771 | 4.985954 | 1.64E-05 | 0.011107 | 2.92866 |
| THEMIS2 | 0.76144 | 6.765431 | 4.94427 | 1.86E-05 | 0.011866 | 2.815304 |
| C2orf88 | -0.870934 | 6.580504 | -4.931334 | 1.94E-05 | 0.011988 | 2.780146 |
| HAND2-AS1 | -0.936044 | 7.578937 | -4.908441 | 2.08E-05 | 0.01249 | 2.717949 |
| MMP12 | 2.97915 | 6.850073 | 4.795409 | 2.92E-05 | 0.016004 | 2.411364 |
| TREM1 | 1.404204 | 7.675971 | 4.791315 | 2.96E-05 | 0.016004 | 2.400278 |
| SPP1 | 2.632831 | 8.538984 | 4.783263 | 3.03E-05 | 0.016004 | 2.378475 |
| AIM1 | 0.762174 | 7.244096 | 4.699077 | 3.90E-05 | 0.018379 | 2.150852 |
| CTSB | 0.586911 | 10.83197 | 4.663956 | 4.34E-05 | 0.01899 | 2.05608 |
| CCL19 | 1.304178 | 9.134268 | 4.658036 | 4.42E-05 | 0.01899 | 2.040117 |
| PABPC4L | -0.682924 | 7.223369 | -4.636772 | 4.71E-05 | 0.01899 | 1.982806 |
| PPAP2B | -0.815005 | 9.587494 | -4.624411 | 4.89E-05 | 0.01899 | 1.949512 |
| VWF | 0.665369 | 7.849003 | 4.617682 | 4.98E-05 | 0.01899 | 1.931393 |
| RAC2 | 0.645967 | 7.391221 | 4.600143 | 5.25E-05 | 0.01899 | 1.884194 |
| MEIS2 | -0.743096 | 10.06144 | -4.59963 | 5.26E-05 | 0.01899 | 1.882811 |
| MMP9 | 2.03746 | 8.16294 | 4.575084 | 5.66E-05 | 0.019779 | 1.816809 |
| F10 | -0.708562 | 7.012426 | -4.547085 | 6.16E-05 | 0.020834 | 1.741605 |
| BEX2 | -0.770558 | 7.141892 | -4.537364 | 6.34E-05 | 0.021118 | 1.715514 |
| WIF1 | -1.468509 | 6.649234 | -4.52541 | 6.57E-05 | 0.021554 | 1.683447 |
| MAOA | -0.806588 | 7.99146 | -4.511293 | 6.85E-05 | 0.021764 | 1.6456 |
| LAPTM5 | 0.77882 | 10.05197 | 4.502373 | 7.04E-05 | 0.021769 | 1.621696 |
| IBSP | 1.645398 | 6.522526 | 4.481152 | 7.50E-05 | 0.022239 | 1.564871 |
| TDO2 | 1.033241 | 4.986149 | 4.463268 | 7.91E-05 | 0.022361 | 1.517025 |
| C5AR1 | 0.938036 | 7.238581 | 4.408016 | 9.32E-05 | 0.025864 | 1.36947 |
| HCK | 0.700353 | 8.049686 | 4.401592 | 9.50E-05 | 0.025864 | 1.35234 |
| S100A8 | 1.456949 | 6.047133 | 4.399616 | 9.56E-05 | 0.025864 | 1.347075 |
| THBS2 | 0.823612 | 11.16435 | 4.388282 | 9.88E-05 | 0.026041 | 1.316869 |
| NEURL2 | 0.700867 | 6.69299 | 4.378379 | 0.000102 | 0.026041 | 1.290494 |
| FCER1G | 0.810623 | 8.350588 | 4.369872 | 0.000104 | 0.026041 | 1.267848 |
| PLAU | 0.764743 | 7.37616 | 4.368298 | 0.000105 | 0.026041 | 1.263659 |
| CXCL5 | 1.308276 | 4.387477 | 4.359912 | 0.000107 | 0.026155 | 1.241347 |
| CD52 | 1.089234 | 6.779554 | 4.325952 | 0.000119 | 0.027584 | 1.151096 |
| TRHDE-AS1 | -0.610053 | 5.085248 | -4.324261 | 0.000119 | 0.027584 | 1.146608 |
| TMEM158 | 0.671023 | 6.683821 | 4.314935 | 0.000123 | 0.027701 | 1.121857 |
| OLFML2B | 0.771347 | 8.52033 | 4.300836 | 0.000128 | 0.028291 | 1.084462 |
| COL5A2 | 0.660826 | 10.03275 | 4.27594 | 0.000138 | 0.02984 | 1.018507 |
| CTSG | 0.782981 | 6.589616 | 4.258741 | 0.000145 | 0.030777 | 0.973003 |
| CHST9 | -0.941573 | 5.118799 | -4.242272 | 0.000152 | 0.031686 | 0.929474 |
| THY1 | 0.719601 | 8.202377 | 4.226524 | 0.000159 | 0.032874 | 0.887892 |
| CTHRC1 | 1.318213 | 10.11842 | 4.208753 | 0.000168 | 0.034037 | 0.84102 |
| CTNNAL1 | -0.687597 | 8.841176 | -4.20198 | 0.000171 | 0.034037 | 0.82317 |
| SLC16A9 | -0.753513 | 7.222148 | -4.168223 | 0.000189 | 0.036577 | 0.734326 |
| C16orf54 | 0.937573 | 6.024464 | 4.155349 | 0.000196 | 0.036665 | 0.700495 |
| ANGPTL7 | -1.019373 | 7.219842 | -4.149 | 0.0002 | 0.036665 | 0.683822 |
| XKR4 | -0.854521 | 4.39603 | -4.148017 | 0.000201 | 0.036665 | 0.681241 |
| COL11A1 | 1.577732 | 5.471074 | 4.146709 | 0.000201 | 0.036665 | 0.677807 |
| GMFG | 0.635496 | 7.888883 | 4.1394 | 0.000206 | 0.037146 | 0.658626 |
| WNK3 | -0.625762 | 5.345027 | -4.103982 | 0.000228 | 0.039552 | 0.565819 |
| COCH | -0.642886 | 7.221148 | -4.060369 | 0.000259 | 0.043276 | 0.451865 |
| ATP1B1 | -0.828161 | 9.553849 | -4.056312 | 0.000262 | 0.04337 | 0.441285 |
| VCAM1 | 0.879202 | 9.58193 | 4.045526 | 0.000271 | 0.04408 | 0.41317 |
| FBP1 | 0.586019 | 6.737817 | 4.040631 | 0.000275 | 0.044379 | 0.400417 |
| IGLC1 | 1.732298 | 7.72444 | 4.028156 | 0.000285 | 0.045677 | 0.367943 |
| SCN7A | -0.96562 | 5.429839 | -3.994035 | 0.000314 | 0.048287 | 0.279279 |
| IGSF10 | -1.037132 | 7.768533 | -3.98745 | 0.00032 | 0.048871 | 0.262196 |
