# Supplementary material for: Development and analysis of a comprehensive diagnostic model for aortic valve calcification using machine learning methods and artificial neural networks
Source: Front Cardiovasc Med. 2022 Dec 1;9:913776. doi: 10.3389/fcvm.2022.913776 (PMC9751025; doi:10.3389/fcvm.2022.913776)
Supplement: Supplementary file 3 [file Table_2.docx]

SUPPLEMENTARY TABLE 2: Metascape analysis results of DEGs of merged data sets of GSE12644 and GSE51472.

| Top 20 clusters with their representative enriched terms (one per cluster). "Count" is the number of genes in the user-provided lists with membership in the given ontology term. "%" is the percentage of all of the user-provided genes that are found in the given ontology term (only input genes with at least one ontology term annotation are included in the calculation). "Log10(P)" is the p-value in log base 10. "Log10(q)" is the multi-test adjusted p-value in log base 10. | | | | | | |
| --- | --- | --- | --- | --- | --- | --- |
| GO | Category | Description | Count | % | Log10(P) | Log10(q) |
| GO:0050900 | GO Biological Processes | leukocyte migration | 15 | 20 | -16.74 | -12.39 |
| M18 | Canonical Pathways | PID INTEGRIN1 PATHWAY | 10 | 13.33 | -15.05 | -11.18 |
| R-HSA-1474244 | Reactome Gene Sets | Extracellular matrix organization | 14 | 18.67 | -13.64 | -10.14 |
| M174 | Canonical Pathways | PID UPA UPAR PATHWAY | 6 | 8 | -9.03 | -5.98 |
| GO:0050801 | GO Biological Processes | ion homeostasis | 14 | 18.67 | -8.35 | -5.4 |
| GO:0030155 | GO Biological Processes | regulation of cell adhesion | 14 | 18.67 | -8.31 | -5.39 |
| M169 | Canonical Pathways | PID INTEGRIN2 PATHWAY | 5 | 6.67 | -8.03 | -5.13 |
| GO:0040017 | GO Biological Processes | positive regulation of locomotion | 12 | 16 | -7.56 | -4.78 |
| GO:0032103 | GO Biological Processes | positive regulation of response to external stimulus | 10 | 13.33 | -6.84 | -4.15 |
| WP2806 | WikiPathways | Complement system | 6 | 8 | -6.75 | -4.07 |
| GO:0002544 | GO Biological Processes | chronic inflammatory response | 3 | 4 | -6.09 | -3.51 |
| M165 | Canonical Pathways | PID SYNDECAN 4 PATHWAY | 4 | 5.33 | -5.92 | -3.37 |
| GO:0050778 | GO Biological Processes | positive regulation of immune response | 10 | 13.33 | -5.91 | -3.37 |
| WP3937 | WikiPathways | Microglia pathogen phagocytosis pathway | 4 | 5.33 | -5.52 | -3.05 |
| R-HSA-109582 | Reactome Gene Sets | Hemostasis | 10 | 13.33 | -5.5 | -3.04 |
| GO:0001775 | GO Biological Processes | cell activation | 10 | 13.33 | -5.41 | -2.96 |
| GO:0001558 | GO Biological Processes | regulation of cell growth | 8 | 10.67 | -5.05 | -2.65 |
| hsa04670 | KEGG Pathway | Leukocyte transendothelial migration | 5 | 6.67 | -5.01 | -2.62 |
| GO:0002526 | GO Biological Processes | acute inflammatory response | 4 | 5.33 | -4.43 | -2.14 |
| GO:2001242 | GO Biological Processes | regulation of intrinsic apoptotic signaling pathway | 5 | 6.67 | -4.16 | -1.92 |
