# Supplementary material for: Development and analysis of a comprehensive diagnostic model for aortic valve calcification using machine learning methods and artificial neural networks
Source: Front Cardiovasc Med. 2022 Dec 1;9:913776. doi: 10.3389/fcvm.2022.913776 (PMC9751025; doi:10.3389/fcvm.2022.913776)
Supplement: Supplementary file 4 [file Table_3.docx]

SUPPLEMENTARY TABLE 3: Gene ontology (GO) analysis results of differentially expressed genes (DEGs) of merged data sets of GSE12644 and GSE51472.

| ONTOLOGY | ID | Description | GeneRatio | BgRatio | pvalue | p.adjust | qvalue | geneID | Count | |
| --- | --- | --- | --- | --- | --- | --- | --- | --- | --- | --- |
| BP | GO:0030595 | leukocyte chemotaxis | 12/66 | 226/18862 | 1.82E-11 | 2.37E-08 | 1.74E-08 | CXCL16/SCG2/CXCL12/PPBP/S100A9/SLAMF8/CCL19/RAC2/C5AR1/S100A8/FCER1G/CXCL5 | | 12 |
| BP | GO:0060326 | cell chemotaxis | 13/66 | 306/18862 | 3.96E-11 | 2.37E-08 | 1.74E-08 | CXCL16/SCG2/CXCL12/PPBP/S100A9/SLAMF8/CCL19/RAC2/C5AR1/S100A8/FCER1G/CXCL5/VCAM1 | | 13 |
| BP | GO:0030198 | extracellular matrix organization | 14/66 | 393/18862 | 6.68E-11 | 2.37E-08 | 1.74E-08 | FN1/LUM/COL4A4/TNC/MMP12/SPP1/VWF/MMP9/IBSP/COL5A2/CTSG/ANGPTL7/COL11A1/VCAM1 | | 14 |
| BP | GO:0043062 | extracellular structure organization | 14/66 | 394/18862 | 6.91E-11 | 2.37E-08 | 1.74E-08 | FN1/LUM/COL4A4/TNC/MMP12/SPP1/VWF/MMP9/IBSP/COL5A2/CTSG/ANGPTL7/COL11A1/VCAM1 | | 14 |
| BP | GO:0045229 | external encapsulating structure organization | 14/66 | 396/18862 | 7.39E-11 | 2.37E-08 | 1.74E-08 | FN1/LUM/COL4A4/TNC/MMP12/SPP1/VWF/MMP9/IBSP/COL5A2/CTSG/ANGPTL7/COL11A1/VCAM1 | | 14 |
| BP | GO:0097530 | granulocyte migration | 10/66 | 148/18862 | 9.43E-11 | 2.52E-08 | 1.85E-08 | SCG2/PPBP/S100A9/SLAMF8/CCL19/RAC2/C5AR1/S100A8/FCER1G/CXCL5 | | 10 |
| BP | GO:0097529 | myeloid leukocyte migration | 11/66 | 218/18862 | 2.35E-10 | 5.37E-08 | 3.95E-08 | SCG2/CXCL12/PPBP/S100A9/SLAMF8/CCL19/RAC2/C5AR1/S100A8/FCER1G/CXCL5 | | 11 |
| BP | GO:1990266 | neutrophil migration | 9/66 | 123/18862 | 4.29E-10 | 8.21E-08 | 6.03E-08 | PPBP/S100A9/SLAMF8/CCL19/RAC2/C5AR1/S100A8/FCER1G/CXCL5 | | 9 |
| BP | GO:0071621 | granulocyte chemotaxis | 9/66 | 124/18862 | 4.62E-10 | 8.21E-08 | 6.03E-08 | SCG2/PPBP/S100A9/CCL19/RAC2/C5AR1/S100A8/FCER1G/CXCL5 | | 9 |
| BP | GO:0030593 | neutrophil chemotaxis | 8/66 | 103/18862 | 2.65E-09 | 4.25E-07 | 3.12E-07 | PPBP/S100A9/CCL19/RAC2/C5AR1/S100A8/FCER1G/CXCL5 | | 8 |
| BP | GO:0043312 | neutrophil degranulation | 12/66 | 485/18862 | 1.02E-07 | 1.45E-05 | 1.07E-05 | PLAUR/PPBP/S100A9/CD93/CTSB/MMP9/C5AR1/S100A8/FCER1G/PLAU/CTSG/GMFG | | 12 |
| BP | GO:0002283 | neutrophil activation involved in immune response | 12/66 | 488/18862 | 1.09E-07 | 1.45E-05 | 1.07E-05 | PLAUR/PPBP/S100A9/CD93/CTSB/MMP9/C5AR1/S100A8/FCER1G/PLAU/CTSG/GMFG | | 12 |
| BP | GO:0002446 | neutrophil mediated immunity | 12/66 | 499/18862 | 1.39E-07 | 1.62E-05 | 1.19E-05 | PLAUR/PPBP/S100A9/CD93/CTSB/MMP9/C5AR1/S100A8/FCER1G/PLAU/CTSG/GMFG | | 12 |
| BP | GO:0042119 | neutrophil activation | 12/66 | 500/18862 | 1.42E-07 | 1.62E-05 | 1.19E-05 | PLAUR/PPBP/S100A9/CD93/CTSB/MMP9/C5AR1/S100A8/FCER1G/PLAU/CTSG/GMFG | | 12 |
| BP | GO:0042742 | defense response to bacterium | 9/66 | 344/18862 | 2.98E-06 | 0.000318 | 0.000234 | PPBP/S100A9/SLAMF8/C5AR1/S100A8/FCER1G/CTSG/COCH/IGLC1 | | 9 |
| BP | GO:0045730 | respiratory burst | 4/66 | 36/18862 | 7.40E-06 | 0.000741 | 0.000544 | SLAMF8/RAC2/HCK/CD52 | | 4 |
| BP | GO:0001503 | ossification | 9/66 | 401/18862 | 1.03E-05 | 0.000969 | 0.000712 | FHL2/TNC/SPP1/MMP9/IBSP/COL5A2/CTHRC1/COL11A1/IGSF10 | | 9 |
| BP | GO:0070486 | leukocyte aggregation | 3/66 | 13/18862 | 1.14E-05 | 0.001015 | 0.000746 | S100A9/RAC2/S100A8 | | 3 |
| BP | GO:1902622 | regulation of neutrophil migration | 4/66 | 42/18862 | 1.38E-05 | 0.001167 | 0.000857 | SLAMF8/CCL19/RAC2/C5AR1 | | 4 |
| BP | GO:0035987 | endodermal cell differentiation | 4/66 | 44/18862 | 1.67E-05 | 0.001337 | 0.000982 | FN1/MMP9/COL5A2/COL11A1 | | 4 |
| BP | GO:0032496 | response to lipopolysaccharide | 8/66 | 326/18862 | 1.76E-05 | 0.001338 | 0.000983 | PPBP/S100A9/C5AR1/HCK/S100A8/CXCL5/CTSG/VCAM1 | | 8 |
| BP | GO:0002237 | response to molecule of bacterial origin | 8/66 | 346/18862 | 2.69E-05 | 0.001957 | 0.001437 | PPBP/S100A9/C5AR1/HCK/S100A8/CXCL5/CTSG/VCAM1 | | 8 |
| BP | GO:0002523 | leukocyte migration involved in inflammatory response | 3/66 | 18/18862 | 3.22E-05 | 0.002239 | 0.001644 | S100A9/SLAMF8/S100A8 | | 3 |
| BP | GO:0001706 | endoderm formation | 4/66 | 53/18862 | 3.52E-05 | 0.002347 | 0.001724 | FN1/MMP9/COL5A2/COL11A1 | | 4 |
| BP | GO:0002544 | chronic inflammatory response | 3/66 | 19/18862 | 3.81E-05 | 0.00244 | 0.001792 | S100A9/S100A8/VCAM1 | | 3 |
| BP | GO:0007159 | leukocyte cell-cell adhesion | 8/66 | 366/18862 | 4.01E-05 | 0.002468 | 0.001813 | CXCL12/S100A9/CCL19/RAC2/LAPTM5/S100A8/THY1/VCAM1 | | 8 |
| BP | GO:0072503 | cellular divalent inorganic cation homeostasis | 9/66 | 480/18862 | 4.24E-05 | 0.002515 | 0.001847 | CXCL12/S100A9/ATP1A2/CCL19/C5AR1/S100A8/CD52/THY1/ATP1B1 | | 9 |
| BP | GO:0006959 | humoral immune response | 8/66 | 380/18862 | 5.22E-05 | 0.002986 | 0.002193 | PPBP/S100A9/TREM1/C5AR1/S100A8/CXCL5/CTSG/IGLC1 | | 8 |
| BP | GO:0072507 | divalent inorganic cation homeostasis | 9/66 | 499/18862 | 5.73E-05 | 0.003161 | 0.002322 | CXCL12/S100A9/ATP1A2/CCL19/C5AR1/S100A8/CD52/THY1/ATP1B1 | | 9 |
| BP | GO:0002688 | regulation of leukocyte chemotaxis | 5/66 | 119/18862 | 6.04E-05 | 0.003222 | 0.002366 | CXCL12/SLAMF8/CCL19/RAC2/C5AR1 | | 5 |
| BP | GO:0090023 | positive regulation of neutrophil chemotaxis | 3/66 | 24/18862 | 7.86E-05 | 0.004047 | 0.002972 | CCL19/RAC2/C5AR1 | | 3 |
| BP | GO:0002685 | regulation of leukocyte migration | 6/66 | 205/18862 | 8.09E-05 | 0.004047 | 0.002972 | CXCL12/SLAMF8/CCL19/RAC2/C5AR1/THY1 | | 6 |
| BP | GO:0002687 | positive regulation of leukocyte migration | 5/66 | 133/18862 | 0.000102 | 0.004964 | 0.003646 | CXCL12/CCL19/RAC2/C5AR1/THY1 | | 5 |
| BP | GO:0071624 | positive regulation of granulocyte chemotaxis | 3/66 | 27/18862 | 0.000113 | 0.005307 | 0.003898 | CCL19/RAC2/C5AR1 | | 3 |
| BP | GO:0072376 | protein activation cascade | 3/66 | 28/18862 | 0.000126 | 0.00545 | 0.004003 | FN1/VWF/F10 | | 3 |
| BP | GO:0072378 | blood coagulation, fibrin clot formation | 3/66 | 28/18862 | 0.000126 | 0.00545 | 0.004003 | FN1/VWF/F10 | | 3 |
| BP | GO:0050921 | positive regulation of chemotaxis | 5/66 | 139/18862 | 0.000126 | 0.00545 | 0.004003 | SCG2/CXCL12/CCL19/RAC2/C5AR1 | | 5 |
| BP | GO:0050920 | regulation of chemotaxis | 6/66 | 224/18862 | 0.000132 | 0.005543 | 0.004071 | SCG2/CXCL12/SLAMF8/CCL19/RAC2/C5AR1 | | 6 |
| BP | GO:0019730 | antimicrobial humoral response | 5/66 | 142/18862 | 0.000139 | 0.005607 | 0.004118 | PPBP/S100A9/S100A8/CXCL5/CTSG | | 5 |
| BP | GO:1902624 | positive regulation of neutrophil migration | 3/66 | 29/18862 | 0.00014 | 0.005607 | 0.004118 | CCL19/RAC2/C5AR1 | | 3 |
| BP | GO:0007492 | endoderm development | 4/66 | 76/18862 | 0.000145 | 0.005668 | 0.004163 | FN1/MMP9/COL5A2/COL11A1 | | 4 |
| BP | GO:0090022 | regulation of neutrophil chemotaxis | 3/66 | 32/18862 | 0.000189 | 0.007195 | 0.005284 | CCL19/RAC2/C5AR1 | | 3 |
| BP | GO:2001242 | regulation of intrinsic apoptotic signaling pathway | 5/66 | 160/18862 | 0.000243 | 0.008898 | 0.006535 | CXCL12/PLAUR/S100A9/MMP9/S100A8 | | 5 |
| BP | GO:0030307 | positive regulation of cell growth | 5/66 | 161/18862 | 0.00025 | 0.008898 | 0.006535 | CXCL16/FN1/CXCL12/S100A9/S100A8 | | 5 |
| BP | GO:0031589 | cell-substrate adhesion | 7/66 | 359/18862 | 0.000253 | 0.008898 | 0.006535 | FN1/MMP12/VWF/RAC2/PLAU/THY1/VCAM1 | | 7 |
| BP | GO:0070098 | chemokine-mediated signaling pathway | 4/66 | 88/18862 | 0.000256 | 0.008898 | 0.006535 | CXCL12/PPBP/CCL19/CXCL5 | | 4 |
| BP | GO:0002690 | positive regulation of leukocyte chemotaxis | 4/66 | 91/18862 | 0.000291 | 0.009903 | 0.007273 | CXCL12/CCL19/RAC2/C5AR1 | | 4 |
| BP | GO:0050832 | defense response to fungus | 3/66 | 39/18862 | 0.000342 | 0.011399 | 0.008371 | S100A9/S100A8/CTSG | | 3 |
| BP | GO:1990868 | response to chemokine | 4/66 | 97/18862 | 0.000371 | 0.01188 | 0.008725 | CXCL12/PPBP/CCL19/CXCL5 | | 4 |
| BP | GO:1990869 | cellular response to chemokine | 4/66 | 97/18862 | 0.000371 | 0.01188 | 0.008725 | CXCL12/PPBP/CCL19/CXCL5 | | 4 |
| BP | GO:0014002 | astrocyte development | 3/66 | 42/18862 | 0.000426 | 0.013376 | 0.009823 | S100A9/C5AR1/S100A8 | | 3 |
| BP | GO:0001558 | regulation of cell growth | 7/66 | 406/18862 | 0.000531 | 0.015961 | 0.011722 | CXCL16/FN1/CXCL12/S100A9/SPP1/S100A8/FBP1 | | 7 |
| BP | GO:0071674 | mononuclear cell migration | 5/66 | 190/18862 | 0.000535 | 0.015961 | 0.011722 | CXCL16/CXCL12/SLAMF8/CCL19/C5AR1 | | 5 |
| BP | GO:0002526 | acute inflammatory response | 4/66 | 107/18862 | 0.000538 | 0.015961 | 0.011722 | FN1/TREM1/S100A8/VCAM1 | | 4 |
| BP | GO:0006816 | calcium ion transport | 7/66 | 409/18862 | 0.000554 | 0.016028 | 0.011771 | CXCL12/GPM6A/ATP1A2/CCL19/THY1/WNK3/ATP1B1 | | 7 |
| BP | GO:0031099 | regeneration | 5/66 | 192/18862 | 0.000561 | 0.016028 | 0.011771 | CXCL12/TNC/SPP1/C5AR1/THY1 | | 5 |
| BP | GO:0030574 | collagen catabolic process | 3/66 | 47/18862 | 0.000594 | 0.016407 | 0.012049 | MMP12/CTSB/MMP9 | | 3 |
| BP | GO:1990573 | potassium ion import across plasma membrane | 3/66 | 47/18862 | 0.000594 | 0.016407 | 0.012049 | ATP1A2/WNK3/ATP1B1 | | 3 |
| BP | GO:0097048 | dendritic cell apoptotic process | 2/66 | 11/18862 | 0.00065 | 0.017341 | 0.012735 | CXCL12/CCL19 | | 2 |
| BP | GO:2000668 | regulation of dendritic cell apoptotic process | 2/66 | 11/18862 | 0.00065 | 0.017341 | 0.012735 | CXCL12/CCL19 | | 2 |
| BP | GO:0071622 | regulation of granulocyte chemotaxis | 3/66 | 49/18862 | 0.000672 | 0.017637 | 0.012953 | CCL19/RAC2/C5AR1 | | 3 |
| BP | GO:0045785 | positive regulation of cell adhesion | 7/66 | 425/18862 | 0.000695 | 0.017668 | 0.012975 | FN1/CXCL12/PLAUR/CCL19/IBSP/THY1/VCAM1 | | 7 |
| BP | GO:0009612 | response to mechanical stimulus | 5/66 | 202/18862 | 0.000705 | 0.017668 | 0.012975 | CXCL12/ATP1A2/TNC/MEIS2/COL11A1 | | 5 |
| BP | GO:0001704 | formation of primary germ layer | 4/66 | 115/18862 | 0.000706 | 0.017668 | 0.012975 | FN1/MMP9/COL5A2/COL11A1 | | 4 |
| BP | GO:0043281 | regulation of cysteine-type endopeptidase activity involved in apoptotic process | 5/66 | 205/18862 | 0.000754 | 0.018333 | 0.013464 | PLAUR/S100A9/MMP9/LAPTM5/S100A8 | | 5 |
| BP | GO:1904645 | response to amyloid-beta | 3/66 | 51/18862 | 0.000756 | 0.018333 | 0.013464 | MMP12/MMP9/VCAM1 | | 3 |
| BP | GO:0051928 | positive regulation of calcium ion transport | 4/66 | 118/18862 | 0.000778 | 0.018587 | 0.013651 | CXCL12/THY1/WNK3/ATP1B1 | | 4 |
| BP | GO:0051651 | maintenance of location in cell | 5/66 | 209/18862 | 0.000822 | 0.019327 | 0.014194 | S100A9/ATP1A2/CCL19/S100A8/THY1 | | 5 |
| BP | GO:0009620 | response to fungus | 3/66 | 53/18862 | 0.000846 | 0.019327 | 0.014194 | S100A9/S100A8/CTSG | | 3 |
| BP | GO:0006874 | cellular calcium ion homeostasis | 7/66 | 442/18862 | 0.000875 | 0.019327 | 0.014194 | CXCL12/ATP1A2/CCL19/C5AR1/CD52/THY1/ATP1B1 | | 7 |
| BP | GO:0030199 | collagen fibril organization | 3/66 | 54/18862 | 0.000893 | 0.019327 | 0.014194 | LUM/COL5A2/COL11A1 | | 3 |
| BP | GO:0055078 | sodium ion homeostasis | 3/66 | 54/18862 | 0.000893 | 0.019327 | 0.014194 | ATP1A2/ATP1B1/SCN7A | | 3 |
| BP | GO:2000649 | regulation of sodium ion transmembrane transporter activity | 3/66 | 54/18862 | 0.000893 | 0.019327 | 0.014194 | ATP1A2/WNK3/ATP1B1 | | 3 |
| BP | GO:0002679 | respiratory burst involved in defense response | 2/66 | 13/18862 | 0.000917 | 0.019327 | 0.014194 | SLAMF8/HCK | | 2 |
| BP | GO:0010248 | establishment or maintenance of transmembrane electrochemical gradient | 2/66 | 13/18862 | 0.000917 | 0.019327 | 0.014194 | ATP1A2/ATP1B1 | | 2 |
| BP | GO:0030007 | cellular potassium ion homeostasis | 2/66 | 13/18862 | 0.000917 | 0.019327 | 0.014194 | ATP1A2/ATP1B1 | | 2 |
| BP | GO:0010810 | regulation of cell-substrate adhesion | 5/66 | 218/18862 | 0.000993 | 0.020647 | 0.015163 | FN1/MMP12/RAC2/PLAU/THY1 | | 5 |
| BP | GO:0055074 | calcium ion homeostasis | 7/66 | 454/18862 | 0.001023 | 0.020996 | 0.01542 | CXCL12/ATP1A2/CCL19/C5AR1/CD52/THY1/ATP1B1 | | 7 |
| BP | GO:0050930 | induction of positive chemotaxis | 2/66 | 14/18862 | 0.001068 | 0.021643 | 0.015895 | SCG2/CXCL12 | | 2 |
| BP | GO:0001649 | osteoblast differentiation | 5/66 | 223/18862 | 0.001099 | 0.021751 | 0.015974 | FHL2/TNC/SPP1/IBSP/CTHRC1 | | 5 |
| BP | GO:0031102 | neuron projection regeneration | 3/66 | 58/18862 | 0.0011 | 0.021751 | 0.015974 | TNC/SPP1/THY1 | | 3 |
| BP | GO:0002697 | regulation of immune effector process | 7/66 | 465/18862 | 0.001175 | 0.022877 | 0.016801 | SLAMF8/MMP12/CCL19/RAC2/LAPTM5/C5AR1/IGLC1 | | 7 |
| BP | GO:0036376 | sodium ion export across plasma membrane | 2/66 | 15/18862 | 0.001229 | 0.022877 | 0.016801 | ATP1A2/ATP1B1 | | 2 |
| BP | GO:0070571 | negative regulation of neuron projection regeneration | 2/66 | 15/18862 | 0.001229 | 0.022877 | 0.016801 | SPP1/THY1 | | 2 |
| BP | GO:0007596 | blood coagulation | 6/66 | 342/18862 | 0.001238 | 0.022877 | 0.016801 | FN1/PLAUR/VWF/F10/FCER1G/PLAU | | 6 |
| BP | GO:0016049 | cell growth | 7/66 | 470/18862 | 0.00125 | 0.022877 | 0.016801 | CXCL16/FN1/CXCL12/S100A9/SPP1/S100A8/FBP1 | | 7 |
| BP | GO:0007160 | cell-matrix adhesion | 5/66 | 230/18862 | 0.00126 | 0.022877 | 0.016801 | FN1/MMP12/PLAU/THY1/VCAM1 | | 5 |
| BP | GO:2000116 | regulation of cysteine-type endopeptidase activity | 5/66 | 230/18862 | 0.00126 | 0.022877 | 0.016801 | PLAUR/S100A9/MMP9/LAPTM5/S100A8 | | 5 |
| BP | GO:0031346 | positive regulation of cell projection organization | 6/66 | 344/18862 | 0.001275 | 0.022877 | 0.016801 | FN1/CXCL12/GPM6A/S100A9/CCL19/RAC2 | | 6 |
| BP | GO:0007599 | hemostasis | 6/66 | 346/18862 | 0.001314 | 0.022877 | 0.016801 | FN1/PLAUR/VWF/F10/FCER1G/PLAU | | 6 |
| BP | GO:0050817 | coagulation | 6/66 | 347/18862 | 0.001333 | 0.022877 | 0.016801 | FN1/PLAUR/VWF/F10/FCER1G/PLAU | | 6 |
| BP | GO:2001233 | regulation of apoptotic signaling pathway | 6/66 | 348/18862 | 0.001353 | 0.022877 | 0.016801 | SCG2/CXCL12/PLAUR/S100A9/MMP9/S100A8 | | 6 |
| BP | GO:0017014 | protein nitrosylation | 2/66 | 16/18862 | 0.001402 | 0.022877 | 0.016801 | S100A9/S100A8 | | 2 |
| BP | GO:0018119 | peptidyl-cysteine S-nitrosylation | 2/66 | 16/18862 | 0.001402 | 0.022877 | 0.016801 | S100A9/S100A8 | | 2 |
| BP | GO:0051238 | sequestering of metal ion | 2/66 | 16/18862 | 0.001402 | 0.022877 | 0.016801 | S100A9/S100A8 | | 2 |
| BP | GO:0060263 | regulation of respiratory burst | 2/66 | 16/18862 | 0.001402 | 0.022877 | 0.016801 | SLAMF8/RAC2 | | 2 |
| BP | GO:2000651 | positive regulation of sodium ion transmembrane transporter activity | 2/66 | 16/18862 | 0.001402 | 0.022877 | 0.016801 | WNK3/ATP1B1 | | 2 |
| BP | GO:0002429 | immune response-activating cell surface receptor signaling pathway | 7/66 | 481/18862 | 0.001427 | 0.022877 | 0.016801 | THEMIS2/LAPTM5/C5AR1/HCK/FCER1G/THY1/IGLC1 | | 7 |
| BP | GO:0002757 | immune response-activating signal transduction | 7/66 | 481/18862 | 0.001427 | 0.022877 | 0.016801 | THEMIS2/LAPTM5/C5AR1/HCK/FCER1G/THY1/IGLC1 | | 7 |
| BP | GO:0055076 | transition metal ion homeostasis | 4/66 | 139/18862 | 0.001429 | 0.022877 | 0.016801 | STEAP1/S100A9/SCARA5/S100A8 | | 4 |
| BP | GO:1902305 | regulation of sodium ion transmembrane transport | 3/66 | 64/18862 | 0.001464 | 0.023003 | 0.016894 | ATP1A2/WNK3/ATP1B1 | | 3 |
| BP | GO:0051924 | regulation of calcium ion transport | 5/66 | 238/18862 | 0.001466 | 0.023003 | 0.016894 | CXCL12/ATP1A2/THY1/WNK3/ATP1B1 | | 5 |
| BP | GO:0030001 | metal ion transport | 7/66 | 486/18862 | 0.001514 | 0.023537 | 0.017286 | CXCL12/GPM6A/ATP1A2/CCL19/THY1/WNK3/ATP1B1 | | 7 |
| BP | GO:0055119 | relaxation of cardiac muscle | 2/66 | 17/18862 | 0.001585 | 0.024172 | 0.017752 | ATP1A2/ATP1B1 | | 2 |
| BP | GO:2001267 | regulation of cysteine-type endopeptidase activity involved in apoptotic signaling pathway | 2/66 | 17/18862 | 0.001585 | 0.024172 | 0.017752 | PLAUR/MMP9 | | 2 |
| BP | GO:0031349 | positive regulation of defense response | 6/66 | 361/18862 | 0.00163 | 0.024583 | 0.018054 | S100A9/MMP12/HCK/S100A8/FCER1G/COCH | | 6 |
| BP | GO:0090066 | regulation of anatomical structure size | 7/66 | 493/18862 | 0.001643 | 0.024583 | 0.018054 | FN1/CXCL12/ATP1A2/SPP1/HCK/GMFG/WNK3 | | 7 |
| BP | GO:0010959 | regulation of metal ion transport | 5/66 | 247/18862 | 0.001725 | 0.025573 | 0.018781 | CXCL12/ATP1A2/THY1/WNK3/ATP1B1 | | 5 |
| BP | GO:0002548 | monocyte chemotaxis | 3/66 | 68/18862 | 0.001743 | 0.025607 | 0.018806 | CXCL12/SLAMF8/CCL19 | | 3 |
| BP | GO:0010766 | negative regulation of sodium ion transport | 2/66 | 18/18862 | 0.001779 | 0.025899 | 0.019021 | ATP1A2/WNK3 | | 2 |
| BP | GO:0032535 | regulation of cellular component size | 6/66 | 370/18862 | 0.001846 | 0.026622 | 0.019552 | FN1/CXCL12/SPP1/HCK/GMFG/WNK3 | | 6 |
| BP | GO:0045123 | cellular extravasation | 3/66 | 70/18862 | 0.001895 | 0.026945 | 0.019789 | CXCL12/THY1/VCAM1 | | 3 |
| BP | GO:0045927 | positive regulation of growth | 5/66 | 253/18862 | 0.001916 | 0.026945 | 0.019789 | CXCL16/FN1/CXCL12/S100A9/S100A8 | | 5 |
| BP | GO:0008360 | regulation of cell shape | 4/66 | 151/18862 | 0.001935 | 0.026945 | 0.019789 | FN1/RAC2/HCK/COCH | | 4 |
| BP | GO:0050770 | regulation of axonogenesis | 4/66 | 151/18862 | 0.001935 | 0.026945 | 0.019789 | FN1/CXCL12/SPP1/THY1 | | 4 |
| BP | GO:0006883 | cellular sodium ion homeostasis | 2/66 | 19/18862 | 0.001984 | 0.027153 | 0.019941 | ATP1A2/ATP1B1 | | 2 |
| BP | GO:0043652 | engulfment of apoptotic cell | 2/66 | 19/18862 | 0.001984 | 0.027153 | 0.019941 | RAC2/XKR4 | | 2 |
| BP | GO:0048708 | astrocyte differentiation | 3/66 | 72/18862 | 0.002054 | 0.02787 | 0.020468 | S100A9/C5AR1/S100A8 | | 3 |
| BP | GO:0006909 | phagocytosis | 6/66 | 381/18862 | 0.002138 | 0.028768 | 0.021128 | CD93/RAC2/HCK/FCER1G/XKR4/IGLC1 | | 6 |
| BP | GO:1902307 | positive regulation of sodium ion transmembrane transport | 2/66 | 21/18862 | 0.002426 | 0.032327 | 0.023741 | WNK3/ATP1B1 | | 2 |
| BP | GO:0055067 | monovalent inorganic cation homeostasis | 4/66 | 161/18862 | 0.002443 | 0.032327 | 0.023741 | SLAMF8/ATP1A2/ATP1B1/SCN7A | | 4 |
| BP | GO:0061844 | antimicrobial humoral immune response mediated by antimicrobial peptide | 3/66 | 78/18862 | 0.002581 | 0.033877 | 0.024879 | PPBP/S100A9/CXCL5 | | 3 |
| BP | GO:0050860 | negative regulation of T cell receptor signaling pathway | 2/66 | 22/18862 | 0.002663 | 0.034656 | 0.025452 | LAPTM5/THY1 | | 2 |
| BP | GO:0022617 | extracellular matrix disassembly | 3/66 | 81/18862 | 0.002874 | 0.037103 | 0.027249 | MMP12/MMP9/CTSG | | 3 |
| BP | GO:0035725 | sodium ion transmembrane transport | 4/66 | 171/18862 | 0.003036 | 0.038885 | 0.028558 | ATP1A2/WNK3/ATP1B1/SCN7A | | 4 |
| BP | GO:0097193 | intrinsic apoptotic signaling pathway | 5/66 | 283/18862 | 0.00311 | 0.039304 | 0.028865 | CXCL12/PLAUR/S100A9/MMP9/S100A8 | | 5 |
| BP | GO:0008361 | regulation of cell size | 4/66 | 173/18862 | 0.003165 | 0.039304 | 0.028865 | FN1/CXCL12/SPP1/WNK3 | | 4 |
| BP | GO:0002407 | dendritic cell chemotaxis | 2/66 | 24/18862 | 0.003167 | 0.039304 | 0.028865 | SLAMF8/CCL19 | | 2 |
| BP | GO:0086064 | cell communication by electrical coupling involved in cardiac conduction | 2/66 | 24/18862 | 0.003167 | 0.039304 | 0.028865 | ATP1A2/ATP1B1 | | 2 |
| BP | GO:0033622 | integrin activation | 2/66 | 25/18862 | 0.003435 | 0.041656 | 0.030593 | FN1/CXCL12 | | 2 |
| BP | GO:0042730 | fibrinolysis | 2/66 | 25/18862 | 0.003435 | 0.041656 | 0.030593 | PLAUR/PLAU | | 2 |
| BP | GO:0050927 | positive regulation of positive chemotaxis | 2/66 | 25/18862 | 0.003435 | 0.041656 | 0.030593 | SCG2/CXCL12 | | 2 |
| BP | GO:0002028 | regulation of sodium ion transport | 3/66 | 87/18862 | 0.003517 | 0.042023 | 0.030862 | ATP1A2/WNK3/ATP1B1 | | 3 |
| BP | GO:0006919 | activation of cysteine-type endopeptidase activity involved in apoptotic process | 3/66 | 87/18862 | 0.003517 | 0.042023 | 0.030862 | S100A9/LAPTM5/S100A8 | | 3 |
| BP | GO:0007369 | gastrulation | 4/66 | 179/18862 | 0.003576 | 0.042407 | 0.031144 | FN1/MMP9/COL5A2/COL11A1 | | 4 |
| BP | GO:0050926 | regulation of positive chemotaxis | 2/66 | 26/18862 | 0.003712 | 0.043702 | 0.032095 | SCG2/CXCL12 | | 2 |
| BP | GO:0046849 | bone remodeling | 3/66 | 89/18862 | 0.00375 | 0.04382 | 0.032182 | SPP1/RAC2/CTHRC1 | | 3 |
| BP | GO:1903035 | negative regulation of response to wounding | 3/66 | 90/18862 | 0.003869 | 0.044891 | 0.032969 | PLAUR/SPP1/PLAU | | 3 |
| BP | GO:0010758 | regulation of macrophage chemotaxis | 2/66 | 27/18862 | 0.004 | 0.045102 | 0.033123 | SLAMF8/C5AR1 | | 2 |
| BP | GO:0010882 | regulation of cardiac muscle contraction by calcium ion signaling | 2/66 | 27/18862 | 0.004 | 0.045102 | 0.033123 | ATP1A2/ATP1B1 | | 2 |
| BP | GO:0090025 | regulation of monocyte chemotaxis | 2/66 | 27/18862 | 0.004 | 0.045102 | 0.033123 | CXCL12/SLAMF8 | | 2 |
| BP | GO:0090200 | positive regulation of release of cytochrome c from mitochondria | 2/66 | 27/18862 | 0.004 | 0.045102 | 0.033123 | PLAUR/MMP9 | | 2 |
| BP | GO:0001819 | positive regulation of cytokine production | 6/66 | 437/18862 | 0.004204 | 0.046554 | 0.03419 | LUM/MMP12/CCL19/LAPTM5/C5AR1/FCER1G | | 6 |
| BP | GO:0022407 | regulation of cell-cell adhesion | 6/66 | 437/18862 | 0.004204 | 0.046554 | 0.03419 | CXCL12/PLAUR/CCL19/LAPTM5/THY1/VCAM1 | | 6 |
| BP | GO:0070588 | calcium ion transmembrane transport | 5/66 | 304/18862 | 0.004216 | 0.046554 | 0.03419 | GPM6A/ATP1A2/CCL19/THY1/ATP1B1 | | 5 |
| BP | GO:2001243 | negative regulation of intrinsic apoptotic signaling pathway | 3/66 | 95/18862 | 0.004503 | 0.049377 | 0.036263 | CXCL12/PLAUR/MMP9 | | 3 |
| BP | GO:0007204 | positive regulation of cytosolic calcium ion concentration | 5/66 | 310/18862 | 0.004578 | 0.049495 | 0.03635 | ATP1A2/CCL19/C5AR1/CD52/THY1 | | 5 |
| BP | GO:0036336 | dendritic cell migration | 2/66 | 29/18862 | 0.004606 | 0.049495 | 0.03635 | SLAMF8/CCL19 | | 2 |
| BP | GO:0055075 | potassium ion homeostasis | 2/66 | 29/18862 | 0.004606 | 0.049495 | 0.03635 | ATP1A2/ATP1B1 | | 2 |
| BP | GO:0098659 | inorganic cation import across plasma membrane | 3/66 | 97/18862 | 0.004773 | 0.050537 | 0.037115 | ATP1A2/WNK3/ATP1B1 | | 3 |
| BP | GO:0099587 | inorganic ion import across plasma membrane | 3/66 | 97/18862 | 0.004773 | 0.050537 | 0.037115 | ATP1A2/WNK3/ATP1B1 | | 3 |
| BP | GO:0045088 | regulation of innate immune response | 5/66 | 315/18862 | 0.004897 | 0.050537 | 0.037115 | SLAMF8/MMP12/HCK/FCER1G/COCH | | 5 |
| BP | GO:0034694 | response to prostaglandin | 2/66 | 30/18862 | 0.004924 | 0.050537 | 0.037115 | TNC/CCL19 | | 2 |
| BP | GO:0042402 | cellular biogenic amine catabolic process | 2/66 | 30/18862 | 0.004924 | 0.050537 | 0.037115 | MAOA/TDO2 | | 2 |
| BP | GO:0050858 | negative regulation of antigen receptor-mediated signaling pathway | 2/66 | 30/18862 | 0.004924 | 0.050537 | 0.037115 | LAPTM5/THY1 | | 2 |
| BP | GO:0050901 | leukocyte tethering or rolling | 2/66 | 30/18862 | 0.004924 | 0.050537 | 0.037115 | CXCL12/VCAM1 | | 2 |
| BP | GO:0034341 | response to interferon-gamma | 4/66 | 197/18862 | 0.005022 | 0.050887 | 0.037372 | CXCL16/CCL19/HCK/VCAM1 | | 4 |
| BP | GO:0071222 | cellular response to lipopolysaccharide | 4/66 | 197/18862 | 0.005022 | 0.050887 | 0.037372 | PPBP/HCK/CXCL5/CTSG | | 4 |
| BP | GO:0052547 | regulation of peptidase activity | 6/66 | 455/18862 | 0.005109 | 0.051154 | 0.037568 | FN1/PLAUR/S100A9/MMP9/LAPTM5/S100A8 | | 6 |
| BP | GO:0019722 | calcium-mediated signaling | 4/66 | 198/18862 | 0.005112 | 0.051154 | 0.037568 | FHL2/ATP1A2/ATP1B1/VCAM1 | | 4 |
| BP | GO:0051235 | maintenance of location | 5/66 | 319/18862 | 0.005162 | 0.051333 | 0.0377 | S100A9/ATP1A2/CCL19/S100A8/THY1 | | 5 |
| BP | GO:0002335 | mature B cell differentiation | 2/66 | 31/18862 | 0.005252 | 0.051586 | 0.037886 | ITM2A/SLAMF8 | | 2 |
| BP | GO:0010644 | cell communication by electrical coupling | 2/66 | 31/18862 | 0.005252 | 0.051586 | 0.037886 | ATP1A2/ATP1B1 | | 2 |
| BP | GO:0010952 | positive regulation of peptidase activity | 4/66 | 200/18862 | 0.005296 | 0.0517 | 0.037969 | FN1/S100A9/LAPTM5/S100A8 | | 4 |
| BP | GO:0050679 | positive regulation of epithelial cell proliferation | 4/66 | 203/18862 | 0.00558 | 0.052953 | 0.038889 | SCG2/CXCL12/MMP12/C5AR1 | | 4 |
| BP | GO:0009310 | amine catabolic process | 2/66 | 32/18862 | 0.00559 | 0.052953 | 0.038889 | MAOA/TDO2 | | 2 |
| BP | GO:0050974 | detection of mechanical stimulus involved in sensory perception | 2/66 | 32/18862 | 0.00559 | 0.052953 | 0.038889 | CXCL12/COL11A1 | | 2 |
| BP | GO:0070570 | regulation of neuron projection regeneration | 2/66 | 32/18862 | 0.00559 | 0.052953 | 0.038889 | SPP1/THY1 | | 2 |
| BP | GO:0090075 | relaxation of muscle | 2/66 | 32/18862 | 0.00559 | 0.052953 | 0.038889 | ATP1A2/ATP1B1 | | 2 |
| BP | GO:0042113 | B cell activation | 5/66 | 326/18862 | 0.005652 | 0.053224 | 0.039088 | ITM2A/SLAMF8/LAPTM5/VCAM1/IGLC1 | | 5 |
| BP | GO:0050863 | regulation of T cell activation | 5/66 | 327/18862 | 0.005724 | 0.053592 | 0.039358 | CCL19/RAC2/LAPTM5/THY1/VCAM1 | | 5 |
| BP | GO:0061387 | regulation of extent of cell growth | 3/66 | 104/18862 | 0.005793 | 0.053926 | 0.039604 | FN1/CXCL12/SPP1 | | 3 |
| BP | GO:1903037 | regulation of leukocyte cell-cell adhesion | 5/66 | 330/18862 | 0.005945 | 0.055021 | 0.040408 | CXCL12/CCL19/LAPTM5/THY1/VCAM1 | | 5 |
| BP | GO:0007229 | integrin-mediated signaling pathway | 3/66 | 106/18862 | 0.006107 | 0.055873 | 0.041033 | FN1/HCK/THY1 | | 3 |
| BP | GO:0120034 | positive regulation of plasma membrane bounded cell projection assembly | 3/66 | 106/18862 | 0.006107 | 0.055873 | 0.041033 | GPM6A/CCL19/RAC2 | | 3 |
| BP | GO:0071219 | cellular response to molecule of bacterial origin | 4/66 | 209/18862 | 0.006178 | 0.056182 | 0.04126 | PPBP/HCK/CXCL5/CTSG | | 4 |
| BP | GO:0042110 | T cell activation | 6/66 | 474/18862 | 0.006211 | 0.056182 | 0.04126 | CCL19/RAC2/LAPTM5/FCER1G/THY1/VCAM1 | | 6 |
| BP | GO:0002691 | regulation of cellular extravasation | 2/66 | 34/18862 | 0.006294 | 0.056292 | 0.041342 | CXCL12/THY1 | | 2 |
| BP | GO:0033280 | response to vitamin D | 2/66 | 34/18862 | 0.006294 | 0.056292 | 0.041342 | TNC/SPP1 | | 2 |
| BP | GO:0032963 | collagen metabolic process | 3/66 | 109/18862 | 0.006597 | 0.058674 | 0.043091 | MMP12/CTSB/MMP9 | | 3 |
| BP | GO:0010765 | positive regulation of sodium ion transport | 2/66 | 35/18862 | 0.00666 | 0.058912 | 0.043265 | WNK3/ATP1B1 | | 2 |
| BP | GO:1904062 | regulation of cation transmembrane transport | 5/66 | 340/18862 | 0.006727 | 0.059178 | 0.043461 | ATP1A2/MMP9/THY1/WNK3/ATP1B1 | | 5 |
| BP | GO:0071675 | regulation of mononuclear cell migration | 3/66 | 111/18862 | 0.006936 | 0.060678 | 0.044562 | CXCL12/SLAMF8/C5AR1 | | 3 |
| BP | GO:0086010 | membrane depolarization during action potential | 2/66 | 36/18862 | 0.007036 | 0.060863 | 0.044699 | ATP1A2/SCN7A | | 2 |
| BP | GO:0051480 | regulation of cytosolic calcium ion concentration | 5/66 | 344/18862 | 0.007059 | 0.060863 | 0.044699 | ATP1A2/CCL19/C5AR1/CD52/THY1 | | 5 |
| BP | GO:0021782 | glial cell development | 3/66 | 112/18862 | 0.007109 | 0.060863 | 0.044699 | S100A9/C5AR1/S100A8 | | 3 |
| BP | GO:0051209 | release of sequestered calcium ion into cytosol | 3/66 | 112/18862 | 0.007109 | 0.060863 | 0.044699 | ATP1A2/CCL19/THY1 | | 3 |
| BP | GO:0051283 | negative regulation of sequestering of calcium ion | 3/66 | 113/18862 | 0.007285 | 0.062037 | 0.045561 | ATP1A2/CCL19/THY1 | | 3 |
| BP | GO:0030004 | cellular monovalent inorganic cation homeostasis | 3/66 | 115/18862 | 0.007644 | 0.064413 | 0.047305 | SLAMF8/ATP1A2/ATP1B1 | | 3 |
| BP | GO:0051282 | regulation of sequestering of calcium ion | 3/66 | 115/18862 | 0.007644 | 0.064413 | 0.047305 | ATP1A2/CCL19/THY1 | | 3 |
| BP | GO:0045089 | positive regulation of innate immune response | 4/66 | 223/18862 | 0.007736 | 0.0645 | 0.04737 | MMP12/HCK/FCER1G/COCH | | 4 |
| BP | GO:0006882 | cellular zinc ion homeostasis | 2/66 | 38/18862 | 0.007816 | 0.0645 | 0.04737 | S100A9/S100A8 | | 2 |
| BP | GO:0048246 | macrophage chemotaxis | 2/66 | 38/18862 | 0.007816 | 0.0645 | 0.04737 | SLAMF8/C5AR1 | | 2 |
| BP | GO:2000249 | regulation of actin cytoskeleton reorganization | 2/66 | 38/18862 | 0.007816 | 0.0645 | 0.04737 | HCK/GMFG | | 2 |
| BP | GO:2001234 | negative regulation of apoptotic signaling pathway | 4/66 | 224/18862 | 0.007856 | 0.0645 | 0.04737 | SCG2/CXCL12/PLAUR/MMP9 | | 4 |
| BP | GO:0060560 | developmental growth involved in morphogenesis | 4/66 | 225/18862 | 0.007978 | 0.065152 | 0.047849 | FN1/CXCL12/TNC/SPP1 | | 4 |
| BP | GO:0046916 | cellular transition metal ion homeostasis | 3/66 | 118/18862 | 0.008203 | 0.065152 | 0.047849 | S100A9/SCARA5/S100A8 | | 3 |
| BP | GO:0072676 | lymphocyte migration | 3/66 | 118/18862 | 0.008203 | 0.065152 | 0.047849 | CXCL16/CXCL12/CCL19 | | 3 |
| BP | GO:0045742 | positive regulation of epidermal growth factor receptor signaling pathway | 2/66 | 39/18862 | 0.00822 | 0.065152 | 0.047849 | PLAUR/MMP9 | | 2 |
| BP | GO:0045773 | positive regulation of axon extension | 2/66 | 39/18862 | 0.00822 | 0.065152 | 0.047849 | FN1/CXCL12 | | 2 |
| BP | GO:1901381 | positive regulation of potassium ion transmembrane transport | 2/66 | 39/18862 | 0.00822 | 0.065152 | 0.047849 | WNK3/ATP1B1 | | 2 |
| BP | GO:1905521 | regulation of macrophage migration | 2/66 | 39/18862 | 0.00822 | 0.065152 | 0.047849 | SLAMF8/C5AR1 | | 2 |
| BP | GO:0030098 | lymphocyte differentiation | 5/66 | 358/18862 | 0.008312 | 0.065555 | 0.048144 | ITM2A/SLAMF8/CCL19/FCER1G/VCAM1 | | 5 |
| BP | GO:0051208 | sequestering of calcium ion | 3/66 | 119/18862 | 0.008394 | 0.065658 | 0.04822 | ATP1A2/CCL19/THY1 | | 3 |
| BP | GO:0001654 | eye development | 5/66 | 359/18862 | 0.008407 | 0.065658 | 0.04822 | GPM6A/MEIS2/COL5A2/THY1/ANGPTL7 | | 5 |
| BP | GO:0043687 | post-translational protein modification | 5/66 | 361/18862 | 0.008599 | 0.065821 | 0.04834 | SCG2/FN1/TNC/SPP1/NEURL2 | | 5 |
| BP | GO:0032735 | positive regulation of interleukin-12 production | 2/66 | 40/18862 | 0.008634 | 0.065821 | 0.04834 | CCL19/LAPTM5 | | 2 |
| BP | GO:0050856 | regulation of T cell receptor signaling pathway | 2/66 | 40/18862 | 0.008634 | 0.065821 | 0.04834 | LAPTM5/THY1 | | 2 |
| BP | GO:0055069 | zinc ion homeostasis | 2/66 | 40/18862 | 0.008634 | 0.065821 | 0.04834 | S100A9/S100A8 | | 2 |
| BP | GO:0071634 | regulation of transforming growth factor beta production | 2/66 | 40/18862 | 0.008634 | 0.065821 | 0.04834 | FN1/LUM | | 2 |
| BP | GO:0150063 | visual system development | 5/66 | 363/18862 | 0.008795 | 0.066732 | 0.049008 | GPM6A/MEIS2/COL5A2/THY1/ANGPTL7 | | 5 |
| BP | GO:0071216 | cellular response to biotic stimulus | 4/66 | 233/18862 | 0.008994 | 0.067402 | 0.0495 | PPBP/HCK/CXCL5/CTSG | | 4 |
| BP | GO:0097305 | response to alcohol | 4/66 | 233/18862 | 0.008994 | 0.067402 | 0.0495 | TNC/CCL19/S100A8/VCAM1 | | 4 |
| BP | GO:0090279 | regulation of calcium ion import | 2/66 | 41/18862 | 0.009056 | 0.067402 | 0.0495 | CXCL12/WNK3 | | 2 |
| BP | GO:1901186 | positive regulation of ERBB signaling pathway | 2/66 | 41/18862 | 0.009056 | 0.067402 | 0.0495 | PLAUR/MMP9 | | 2 |
| BP | GO:0050727 | regulation of inflammatory response | 5/66 | 366/18862 | 0.009094 | 0.067402 | 0.0495 | S100A9/SLAMF8/MMP9/HCK/S100A8 | | 5 |
| CC | GO:0062023 | collagen-containing extracellular matrix | 17/71 | 423/19520 | 1.32E-13 | 1.96E-11 | 1.45E-11 | FN1/LUM/CXCL12/S100A9/COL4A4/TNC/CTSB/VWF/MMP9/S100A8/THBS2/COL5A2/CTSG/CTHRC1/ANGPTL7/COL11A1/COCH | | 17 |
| CC | GO:0098644 | complex of collagen trimers | 4/71 | 21/19520 | 9.18E-07 | 6.79E-05 | 5.02E-05 | LUM/COL4A4/COL5A2/COL11A1 | | 4 |
| CC | GO:0005788 | endoplasmic reticulum lumen | 9/71 | 306/19520 | 1.61E-06 | 7.97E-05 | 5.89E-05 | SCG2/FN1/PLAUR/COL4A4/TNC/SPP1/F10/COL5A2/COL11A1 | | 9 |
| CC | GO:0005583 | fibrillar collagen trimer | 3/71 | 12/19520 | 9.91E-06 | 0.000293 | 0.000217 | LUM/COL5A2/COL11A1 | | 3 |
| CC | GO:0098643 | banded collagen fibril | 3/71 | 12/19520 | 9.91E-06 | 0.000293 | 0.000217 | LUM/COL5A2/COL11A1 | | 3 |
| CC | GO:0005581 | collagen trimer | 5/71 | 87/19520 | 1.62E-05 | 0.000399 | 0.000295 | LUM/COL4A4/COL5A2/CTHRC1/COL11A1 | | 5 |
| CC | GO:0034774 | secretory granule lumen | 7/71 | 322/19520 | 0.000168 | 0.003088 | 0.002284 | FN1/PPBP/S100A9/VWF/S100A8/CTSG/GMFG | | 7 |
| CC | GO:0060205 | cytoplasmic vesicle lumen | 7/71 | 326/19520 | 0.000181 | 0.003088 | 0.002284 | FN1/PPBP/S100A9/VWF/S100A8/CTSG/GMFG | | 7 |
| CC | GO:0031983 | vesicle lumen | 7/71 | 328/19520 | 0.000188 | 0.003088 | 0.002284 | FN1/PPBP/S100A9/VWF/S100A8/CTSG/GMFG | | 7 |
| CC | GO:0070820 | tertiary granule | 5/71 | 164/19520 | 0.000328 | 0.004552 | 0.003367 | PPBP/CD93/MMP9/FCER1G/PLAU | | 5 |
| CC | GO:0031091 | platelet alpha granule | 4/71 | 91/19520 | 0.000338 | 0.004552 | 0.003367 | FN1/PPBP/VWF/THBS2 | | 4 |
| CC | GO:0101002 | ficolin-1-rich granule | 5/71 | 185/19520 | 0.000569 | 0.007013 | 0.005187 | CD93/CTSB/MMP9/FCER1G/GMFG | | 5 |
| CC | GO:0009897 | external side of plasma membrane | 7/71 | 402/19520 | 0.000638 | 0.007268 | 0.005376 | CXCL12/SCARA5/F10/FCER1G/THY1/VCAM1/IGLC1 | | 7 |
| CC | GO:0005925 | focal adhesion | 7/71 | 416/19520 | 0.000781 | 0.007962 | 0.00589 | FHL2/PLAUR/TNC/RAC2/HCK/PLAU/THY1 | | 7 |
| CC | GO:0005890 | sodium:potassium-exchanging ATPase complex | 2/71 | 12/19520 | 0.000841 | 0.007962 | 0.00589 | ATP1A2/ATP1B1 | | 2 |
| CC | GO:0030055 | cell-substrate junction | 7/71 | 423/19520 | 0.000861 | 0.007962 | 0.00589 | FHL2/PLAUR/TNC/RAC2/HCK/PLAU/THY1 | | 7 |
| CC | GO:0031093 | platelet alpha granule lumen | 3/71 | 67/19520 | 0.00187 | 0.015803 | 0.011689 | FN1/PPBP/VWF | | 3 |
| CC | GO:0098533 | ATPase dependent transmembrane transport complex | 2/71 | 18/19520 | 0.001922 | 0.015803 | 0.011689 | ATP1A2/ATP1B1 | | 2 |
| CC | GO:0070821 | tertiary granule membrane | 3/71 | 73/19520 | 0.00239 | 0.018618 | 0.013772 | CD93/FCER1G/PLAU | | 3 |
| CC | GO:0005901 | caveola | 3/71 | 80/19520 | 0.003101 | 0.022137 | 0.016374 | ATP1A2/HCK/ATP1B1 | | 3 |
| CC | GO:0031233 | intrinsic component of external side of plasma membrane | 2/71 | 23/19520 | 0.003141 | 0.022137 | 0.016374 | F10/THY1 | | 2 |
| CC | GO:0045177 | apical part of cell | 6/71 | 414/19520 | 0.003937 | 0.026487 | 0.019592 | FN1/CTSB/C5AR1/THY1/ATP1B1/VCAM1 | | 6 |
| CC | GO:0035579 | specific granule membrane | 3/71 | 91/19520 | 0.004456 | 0.028674 | 0.02121 | PLAUR/CD93/PLAU | | 3 |
| CC | GO:0005604 | basement membrane | 3/71 | 94/19520 | 0.004879 | 0.029964 | 0.022164 | FN1/COL4A4/TNC | | 3 |
| CC | GO:0030667 | secretory granule membrane | 5/71 | 305/19520 | 0.005062 | 0.029964 | 0.022164 | PLAUR/CD93/C5AR1/FCER1G/PLAU | | 5 |
| CC | GO:0044853 | plasma membrane raft | 3/71 | 111/19520 | 0.007727 | 0.043984 | 0.032535 | ATP1A2/HCK/ATP1B1 | | 3 |
| CC | GO:1904813 | ficolin-1-rich granule lumen | 3/71 | 124/19520 | 0.010448 | 0.057269 | 0.042361 | CTSB/MMP9/GMFG | | 3 |
| CC | GO:0042383 | sarcolemma | 3/71 | 134/19520 | 0.012874 | 0.067411 | 0.049863 | ATP1A2/ATP1B1/VCAM1 | | 3 |
| CC | GO:0014704 | intercalated disc | 2/71 | 48/19520 | 0.013209 | 0.067411 | 0.049863 | ATP1A2/ATP1B1 | | 2 |
| MF | GO:0005518 | collagen binding | 7/67 | 68/18337 | 5.12E-09 | 1.13E-06 | 9.06E-07 | FN1/LUM/MMP12/CTSB/VWF/MMP9/COCH | | 7 |
| MF | GO:0005201 | extracellular matrix structural constituent | 9/67 | 170/18337 | 1.10E-08 | 1.22E-06 | 9.75E-07 | FN1/LUM/COL4A4/TNC/VWF/THBS2/COL5A2/CTHRC1/COL11A1 | | 9 |
| MF | GO:0005178 | integrin binding | 7/67 | 142/18337 | 8.49E-07 | 5.20E-05 | 4.16E-05 | FN1/CXCL12/SPP1/VWF/IBSP/THY1/VCAM1 | | 7 |
| MF | GO:0008009 | chemokine activity | 5/67 | 49/18337 | 9.42E-07 | 5.20E-05 | 4.16E-05 | CXCL16/CXCL12/PPBP/CCL19/CXCL5 | | 5 |
| MF | GO:0042379 | chemokine receptor binding | 5/67 | 69/18337 | 5.25E-06 | 0.000232 | 0.000186 | CXCL16/CXCL12/PPBP/CCL19/CXCL5 | | 5 |
| MF | GO:0005125 | cytokine activity | 7/67 | 235/18337 | 2.35E-05 | 0.000865 | 0.000692 | CXCL16/SCG2/CXCL12/PPBP/SPP1/CCL19/CXCL5 | | 7 |
| MF | GO:0045236 | CXCR chemokine receptor binding | 3/67 | 18/18337 | 3.66E-05 | 0.001155 | 0.000924 | CXCL12/PPBP/CXCL5 | | 3 |
| MF | GO:0043394 | proteoglycan binding | 3/67 | 36/18337 | 0.000305 | 0.008436 | 0.00675 | FN1/TNC/CTSB | | 3 |
| MF | GO:0048018 | receptor ligand activity | 8/67 | 486/18337 | 0.00038 | 0.009041 | 0.007235 | CXCL16/SCG2/CXCL12/PPBP/SPP1/CCL19/CXCL5/GMFG | | 8 |
| MF | GO:0030546 | signaling receptor activator activity | 8/67 | 492/18337 | 0.000413 | 0.009041 | 0.007235 | CXCL16/SCG2/CXCL12/PPBP/SPP1/CCL19/CXCL5/GMFG | | 8 |
| MF | GO:0030020 | extracellular matrix structural constituent conferring tensile strength | 3/67 | 41/18337 | 0.00045 | 0.009041 | 0.007235 | COL4A4/COL5A2/COL11A1 | | 3 |
| MF | GO:0050786 | RAGE receptor binding | 2/67 | 10/18337 | 0.000581 | 0.010695 | 0.008558 | S100A9/S100A8 | | 2 |
| MF | GO:0001664 | G protein-coupled receptor binding | 6/67 | 289/18337 | 0.000649 | 0.011025 | 0.008822 | CXCL16/CXCL12/PPBP/CCL19/CXCL5/CTHRC1 | | 6 |
| MF | GO:0035325 | Toll-like receptor binding | 2/67 | 12/18337 | 0.000848 | 0.013382 | 0.010708 | S100A9/S100A8 | | 2 |
| MF | GO:0036041 | long-chain fatty acid binding | 2/67 | 13/18337 | 0.001 | 0.014726 | 0.011784 | S100A9/S100A8 | | 2 |
| MF | GO:0016641 | oxidoreductase activity, acting on the CH-NH2 group of donors, oxygen as acceptor | 2/67 | 16/18337 | 0.001527 | 0.02109 | 0.016876 | MAOA/VCAM1 | | 2 |
| MF | GO:0016638 | oxidoreductase activity, acting on the CH-NH2 group of donors | 2/67 | 20/18337 | 0.002395 | 0.031133 | 0.024913 | MAOA/VCAM1 | | 2 |
| MF | GO:0001848 | complement binding | 2/67 | 21/18337 | 0.002641 | 0.032422 | 0.025944 | CD93/C5AR1 | | 2 |
| MF | GO:0008201 | heparin binding | 4/67 | 164/18337 | 0.003052 | 0.033834 | 0.027074 | FN1/THBS2/CTSG/COL11A1 | | 4 |
| MF | GO:0005126 | cytokine receptor binding | 5/67 | 270/18337 | 0.003062 | 0.033834 | 0.027074 | CXCL16/CXCL12/PPBP/CCL19/CXCL5 | | 5 |
| MF | GO:0004252 | serine-type endopeptidase activity | 4/67 | 168/18337 | 0.003327 | 0.034619 | 0.027702 | MMP9/F10/PLAU/CTSG | | 4 |
| MF | GO:0019865 | immunoglobulin binding | 2/67 | 24/18337 | 0.003446 | 0.034619 | 0.027702 | VWF/FCER1G | | 2 |
| MF | GO:0008236 | serine-type peptidase activity | 4/67 | 186/18337 | 0.004777 | 0.045522 | 0.036426 | MMP9/F10/PLAU/CTSG | | 4 |
| MF | GO:0017171 | serine hydrolase activity | 4/67 | 188/18337 | 0.004961 | 0.045522 | 0.036426 | MMP9/F10/PLAU/CTSG | | 4 |
| MF | GO:0004175 | endopeptidase activity | 6/67 | 438/18337 | 0.005244 | 0.045522 | 0.036426 | MMP12/CTSB/MMP9/F10/PLAU/CTSG | | 6 |
| MF | GO:0017147 | Wnt-protein binding | 2/67 | 30/18337 | 0.005356 | 0.045522 | 0.036426 | WIF1/CTHRC1 | | 2 |
| MF | GO:0051087 | chaperone binding | 3/67 | 101/18337 | 0.00602 | 0.049274 | 0.039428 | FN1/ATP1A2/VWF | | 3 |
