# Supplementary material for: Development and analysis of a comprehensive diagnostic model for aortic valve calcification using machine learning methods and artificial neural networks
Source: Front Cardiovasc Med. 2022 Dec 1;9:913776. doi: 10.3389/fcvm.2022.913776 (PMC9751025; doi:10.3389/fcvm.2022.913776)
Supplement: Supplementary file 5 [file Table_4.docx]

SUPPLEMENTARY TABLE 4: Kyoto encyclopedia of genes and genomes (KEGG) analysis results of differentially expressed genes (DEGs) of merged data sets of GSE12644 and GSE51472.

| pathway | Description | GeneRatio | BgRatio | pvalue | p.adjust | qvalue | geneID | Count |
| --- | --- | --- | --- | --- | --- | --- | --- | --- |
| hsa04512 | ECM-receptor interaction | 7/44 | 88/8113 | 3.84E-07 | 5.07E-05 | 4.16E-05 | FN1/COL4A4/TNC/SPP1/VWF/IBSP/THBS2 | 7 |
| hsa04510 | Focal adhesion | 8/44 | 201/8113 | 1.02E-05 | 0.000671 | 0.000551 | FN1/COL4A4/TNC/SPP1/VWF/RAC2/IBSP/THBS2 | 8 |
| hsa04062 | Chemokine signaling pathway | 7/44 | 192/8113 | 6.80E-05 | 0.002915 | 0.002394 | CXCL16/CXCL12/PPBP/CCL19/RAC2/HCK/CXCL5 | 7 |
| hsa04610 | Complement and coagulation cascades | 5/44 | 85/8113 | 8.83E-05 | 0.002915 | 0.002394 | PLAUR/VWF/F10/C5AR1/PLAU | 5 |
| hsa04974 | Protein digestion and absorption | 5/44 | 103/8113 | 0.000219 | 0.005789 | 0.004755 | COL4A4/ATP1A2/COL5A2/COL11A1/ATP1B1 | 5 |
| hsa04670 | Leukocyte transendothelial migration | 5/44 | 114/8113 | 0.000352 | 0.007741 | 0.006359 | CXCL12/RAC2/MMP9/THY1/VCAM1 | 5 |
| hsa00380 | Tryptophan metabolism | 3/44 | 42/8113 | 0.001474 | 0.026557 | 0.021813 | ALDH2/MAOA/TDO2 | 3 |
| hsa04657 | IL-17 signaling pathway | 4/44 | 94/8113 | 0.00161 | 0.026557 | 0.021813 | S100A9/MMP9/S100A8/CXCL5 | 4 |
| hsa05165 | Human papillomavirus infection | 7/44 | 331/8113 | 0.001839 | 0.026683 | 0.021917 | FN1/COL4A4/TNC/SPP1/VWF/IBSP/THBS2 | 7 |
| hsa04061 | Viral protein interaction with cytokine and cytokine receptor | 4/44 | 100/8113 | 0.002021 | 0.026683 | 0.021917 | CXCL12/PPBP/CCL19/CXCL5 | 4 |
| hsa04064 | NF-kappa B signaling pathway | 4/44 | 104/8113 | 0.002333 | 0.028001 | 0.023 | CXCL12/CCL19/PLAU/VCAM1 | 4 |
| hsa04151 | PI3K-Akt signaling pathway | 7/44 | 354/8113 | 0.002693 | 0.029626 | 0.024334 | FN1/COL4A4/TNC/SPP1/VWF/IBSP/THBS2 | 7 |
| hsa04978 | Mineral absorption | 3/44 | 60/8113 | 0.004106 | 0.041695 | 0.034247 | STEAP1/ATP1A2/ATP1B1 | 3 |
| hsa05205 | Proteoglycans in cancer | 5/44 | 205/8113 | 0.004783 | 0.045094 | 0.037039 | FN1/LUM/PLAUR/MMP9/PLAU | 5 |
| hsa00340 | Histidine metabolism | 2/44 | 22/8113 | 0.006198 | 0.054547 | 0.044803 | ALDH2/MAOA | 2 |
| hsa04964 | Proximal tubule bicarbonate reclamation | 2/44 | 23/8113 | 0.006766 | 0.055816 | 0.045845 | ATP1A2/ATP1B1 | 2 |
