# Supplementary material for: Development and analysis of a comprehensive diagnostic model for aortic valve calcification using machine learning methods and artificial neural networks
Source: Front Cardiovasc Med. 2022 Dec 1;9:913776. doi: 10.3389/fcvm.2022.913776 (PMC9751025; doi:10.3389/fcvm.2022.913776)
Supplement: Supplementary file 6 [file Table_5.docx]

SUPPLEMENTARY TABLE 5: Identification of key genes of differentially expressed genes (DEGs) of merged data sets of GSE12644 and GSE51472 by SVM-RFE technique.

| genes |
| --- |
| PLTP |
| FN1 |
| CXCL16 |
| GPM6A |
| LUM |
| FHL2 |
| BEX2 |
| ATP1A2 |
| SCG2 |
| S100A9 |
| CD93 |
| CCL19 |
| COL4A4 |
| ITM2A |
| OLFML2B |
| SCARA5 |
| PPAP2B |
| WIF1 |
| IGSF10 |
| PPBP |
| MMP12 |
| C2orf88 |
