# Supplementary material for: Development and analysis of a comprehensive diagnostic model for aortic valve calcification using machine learning methods and artificial neural networks
Source: Front Cardiovasc Med. 2022 Dec 1;9:913776. doi: 10.3389/fcvm.2022.913776 (PMC9751025; doi:10.3389/fcvm.2022.913776)
Supplement: Supplementary file 7 [file Table_6.docx]

SUPPLEMENTARY TABLE 6: Identification of key genes of differentially expressed genes (DEGs) of merged data sets of GSE12644 and GSE51472 by random

forest (RF) technique.

| genes |
| --- |
| BEX2 |
| S100A9 |
| CXCL16 |
| TRHDEAS1 |
| GPM6A |
| SLC16A9 |
| THBS2 |
| SCARA5 |
