# Supplementary material for: Development and analysis of a comprehensive diagnostic model for aortic valve calcification using machine learning methods and artificial neural networks
Source: Front Cardiovasc Med. 2022 Dec 1;9:913776. doi: 10.3389/fcvm.2022.913776 (PMC9751025; doi:10.3389/fcvm.2022.913776)
Supplement: Supplementary file 8 [file Table_7.docx]

SUPPLEMENTARY TABLE 7: Results of immune cell infiltration of merged data sets of GSE12644 and GSE51472 by CIBERSORT.

| id | B cells naive | B cells memory | Plasma cells | T cells CD8 | T cells CD4 naive | T cells CD4 memory resting | T cells CD4 memory activated | T cells follicular helper | T cells regulatory (Tregs) | T cells gamma delta | NK cells resting | NK cells activated | Monocytes | Macrophages M0 | Macrophages M1 | Macrophages M2 | Dendritic cells resting | Dendritic cells activated | Mast cells resting | Mast cells activated | Eosinophils | Neutrophils |
| --- | --- | --- | --- | --- | --- | --- | --- | --- | --- | --- | --- | --- | --- | --- | --- | --- | --- | --- | --- | --- | --- | --- |
| GSM317342_con | 0.008419 | 0 | 0.055619 | 0 | 0.037062 | 0.05828 | 0 | 0.022773 | 0 | 0.141343 | 0 | 0.049606 | 0.008436 | 0.002652 | 0.074616 | 0.333692 | 0.109096 | 0 | 0.015577 | 0.046213 | 0 | 0.036617 |
| GSM317343_con | 0 | 0 | 0.043739 | 0.007118 | 0.028135 | 0.007368 | 0 | 0.038129 | 0 | 0.052242 | 0 | 0.039368 | 0.044461 | 0 | 0.069382 | 0.493196 | 0 | 0.000156 | 0.119434 | 0 | 0 | 0.05727 |
| GSM317344_con | 0.059503 | 0 | 0.022763 | 0 | 0.013034 | 0.098515 | 0 | 0.009237 | 0 | 0.089012 | 0 | 0.050096 | 0.004742 | 0 | 0.100924 | 0.326385 | 0.044653 | 0 | 0.137489 | 0 | 0 | 0.043647 |
| GSM317345_con | 0.001484 | 0 | 0.05076 | 0.010328 | 0.049583 | 0.007805 | 0 | 0.044768 | 0 | 0.01496 | 0 | 0.040777 | 0.029919 | 0.030293 | 0.054802 | 0.534335 | 0.012951 | 0 | 0.099715 | 0 | 0.003654 | 0.013866 |
| GSM317346_con | 0.019471 | 0 | 0.038246 | 0.053163 | 0.013765 | 0.054176 | 0 | 0.036206 | 0 | 0 | 0 | 0.071097 | 0.032201 | 0 | 0.056905 | 0.486331 | 0.002362 | 0 | 0.112207 | 0 | 0 | 0.023871 |
| GSM377368_con | 0.016911 | 0 | 0.008384 | 0.164866 | 0 | 0.11644 | 0 | 0.02639 | 0.028013 | 0 | 0.068117 | 0 | 0.009058 | 0 | 0.056396 | 0.346331 | 0.06725 | 0 | 0.085907 | 0 | 0 | 0.005938 |
| GSM377369_con | 0.005875 | 0.006623 | 0.016097 | 0.187712 | 0 | 0.077838 | 0 | 0.034843 | 0.051848 | 0 | 0.041342 | 0 | 0.008966 | 0 | 0.051393 | 0.361551 | 0.059676 | 0 | 0.072093 | 0.016534 | 0 | 0.007608 |
| GSM377370_con | 0.008391 | 0.003789 | 0.025714 | 0.038531 | 0 | 0.134381 | 0 | 0.030599 | 0.066355 | 0 | 0.010376 | 0.020006 | 0.008625 | 0 | 0.061901 | 0.479994 | 0.0406 | 0 | 0.06312 | 0 | 0 | 0.007616 |
| GSM377371_con | 0.009729 | 0 | 0.019683 | 0.085645 | 0 | 0.143636 | 0 | 0.007273 | 0.045794 | 0 | 0.095512 | 0 | 0.01064 | 0 | 0.049431 | 0.448219 | 0.020238 | 0 | 0.04591 | 0.008343 | 0 | 0.009947 |
| GSM377372_con | 0.022575 | 0 | 0.018144 | 0.144782 | 0 | 0.14217 | 0 | 0.039745 | 0.047184 | 0 | 0.048198 | 0 | 0.012317 | 0.00026 | 0.049402 | 0.340654 | 0.028969 | 0 | 0 | 0.089732 | 0 | 0.015869 |
| GSM1246204_con | 0.061642 | 0 | 0.001477 | 0.054272 | 0 | 0.176451 | 0 | 0.013155 | 0.013219 | 0 | 0 | 0.059626 | 0.012441 | 0 | 0.093091 | 0.444002 | 0.020101 | 0 | 0.021535 | 0.014941 | 0 | 0.014046 |
| GSM1246205_con | 0.050864 | 0 | 0 | 0.041227 | 0 | 0.054156 | 0 | 0 | 0 | 0.038483 | 0 | 0.009043 | 0 | 0 | 0.037484 | 0.586936 | 0.025458 | 0 | 0.130476 | 0 | 0 | 0.025873 |
| GSM1246206_con | 0.041998 | 0 | 0.010177 | 0.070128 | 0 | 0.115983 | 0 | 0 | 0.053454 | 0 | 0 | 0.046293 | 0 | 0 | 0.087825 | 0.481207 | 0.019947 | 0 | 0.054451 | 0 | 0 | 0.018537 |
| GSM1246207_con | 0.029614 | 0 | 0.004555 | 0.072354 | 0 | 0.113266 | 0 | 0.01734 | 0.042041 | 0 | 0.00437 | 0.021684 | 0.016271 | 0 | 0.061778 | 0.499487 | 0.003627 | 0 | 0.100885 | 0 | 0 | 0.012729 |
| GSM1246208_con | 0.067753 | 0 | 0 | 0.01746 | 0 | 0.088739 | 0 | 0.027997 | 0.020964 | 0 | 0.020571 | 0 | 0.00409 | 0 | 0.185445 | 0.492842 | 0.033505 | 0 | 0 | 0.038312 | 0 | 0.002322 |
| GSM317347_treat | 0 | 0 | 0.046564 | 0.017484 | 0.042552 | 0.020443 | 0 | 0.018446 | 0 | 0.054327 | 0 | 0.004446 | 0 | 0 | 0.082875 | 0.510407 | 0.054643 | 0 | 0.071075 | 0.020745 | 0 | 0.055993 |
| GSM317348_treat | 0.00617 | 0 | 0.030828 | 0 | 0.102599 | 0.017529 | 0.03448 | 0 | 0 | 0.139977 | 0 | 0.009886 | 0.016768 | 0.017952 | 0.088336 | 0.394388 | 0.018126 | 0 | 0.096909 | 0 | 0 | 0.026052 |
| GSM317349_treat | 0 | 0 | 0.055785 | 0.002747 | 0.052396 | 0.010697 | 0.009163 | 0.018615 | 0 | 0.136135 | 0 | 0.01633 | 0.015039 | 0 | 0.110049 | 0.383212 | 0.056338 | 0 | 0.102338 | 0 | 0 | 0.031156 |
| GSM317350_treat | 0.016788 | 0 | 0.053174 | 0.004136 | 0.022101 | 0.037409 | 0.02642 | 0.019277 | 0 | 0.145693 | 0 | 0 | 0 | 0.223022 | 0.064937 | 0.291934 | 0.009191 | 0 | 0.073071 | 0 | 0 | 0.012847 |
| GSM317351_treat | 0 | 0 | 0.046261 | 0 | 0.039954 | 0.050989 | 0.00107 | 0.007031 | 0 | 0.155116 | 0 | 0 | 0.001137 | 0.306224 | 0.056243 | 0.269432 | 0 | 0 | 0.016509 | 0.014956 | 0 | 0.035076 |
| GSM377373_treat | 0 | 0.062997 | 0.027012 | 0.104905 | 0 | 0.009922 | 0 | 0.026511 | 0.051459 | 0 | 0.093641 | 0 | 0 | 0.153269 | 0.035524 | 0.314311 | 0.029008 | 0 | 0 | 0.081064 | 0 | 0.010376 |
| GSM377374_treat | 0.010451 | 0 | 0.010581 | 0.1538 | 0 | 0.087509 | 0 | 0.022617 | 0.054255 | 0 | 0.068415 | 0 | 0.005311 | 0 | 0.061191 | 0.3834 | 0.084722 | 0 | 0.031774 | 0.010081 | 0 | 0.015893 |
| GSM377375_treat | 0.006551 | 0.004967 | 0.019079 | 0.10858 | 0 | 0.110812 | 0 | 0.034938 | 0.051644 | 0 | 0.044223 | 0 | 0.00897 | 0 | 0.061861 | 0.427558 | 0.045428 | 0 | 0.053056 | 0.013759 | 0 | 0.008574 |
| GSM377376_treat | 0.009743 | 0.01027 | 0.022179 | 0.069513 | 0 | 0.024105 | 0 | 0.025057 | 0.061504 | 0 | 0.066882 | 0 | 0 | 0.215183 | 0.022944 | 0.330757 | 0.055234 | 0 | 0.041666 | 0.020236 | 0 | 0.024726 |
| GSM377377_treat | 0.002046 | 0.021948 | 0.017332 | 0.118067 | 0 | 0.116324 | 0 | 0.02967 | 0.031873 | 0 | 0.063079 | 0 | 0.003058 | 0 | 0.053421 | 0.457051 | 0.019885 | 0 | 0.044926 | 0.009576 | 0 | 0.011745 |
| GSM1246209_treat | 0.023704 | 0 | 0.026866 | 0.094278 | 0 | 0.135929 | 0 | 0 | 0.039275 | 0 | 0.000467 | 0.01385 | 0.002661 | 0 | 0.068251 | 0.489351 | 0.013222 | 0 | 0.074791 | 0 | 0 | 0.017355 |
| GSM1246210_treat | 0 | 0 | 0.019644 | 0.070701 | 0 | 0.064207 | 0 | 0.025177 | 0.023511 | 0 | 0 | 0.006258 | 0.040204 | 0 | 0.099929 | 0.479316 | 0.029724 | 0 | 0.106672 | 0 | 0 | 0.034658 |
| GSM1246211_treat | 0.063336 | 0 | 0 | 0.01461 | 0 | 0.083562 | 0 | 0 | 0.029619 | 0.007413 | 0 | 0.013343 | 0.001184 | 0 | 0.052625 | 0.541289 | 0.002626 | 0 | 0.170739 | 0 | 0 | 0.019654 |
| GSM1246212_treat | 0 | 0.007655 | 0.244603 | 0.048546 | 0.045256 | 0 | 0 | 0.025227 | 0.037907 | 0.019953 | 0.019196 | 0 | 0 | 0 | 0.080591 | 0.371468 | 0.057111 | 0 | 0.017935 | 0.003955 | 0 | 0.020596 |
| GSM1246213_treat | 0 | 0.00416 | 0.019892 | 0.091823 | 0 | 0.105052 | 0 | 0.006756 | 0.012343 | 0 | 0.025831 | 0 | 0 | 0.27262 | 0.031821 | 0.374326 | 0.013766 | 0 | 0.014881 | 0.016258 | 0 | 0.010471 |
| GSM1246214_treat | 0 | 0.031806 | 0.039063 | 0.049692 | 0 | 0.045598 | 0 | 0.056485 | 0 | 0.085308 | 0 | 0 | 0 | 0.155384 | 0.057752 | 0.372244 | 0.008901 | 0 | 0.030989 | 0.032577 | 0 | 0.034201 |
| GSM1246215_treat | 0.011443 | 0 | 0.077268 | 0.117809 | 0 | 0.061919 | 0 | 0.03349 | 0.009023 | 0.012573 | 0.018044 | 0 | 0.029671 | 0.041915 | 0.06978 | 0.383533 | 0.013034 | 0 | 0.084496 | 0.009261 | 0 | 0.02674 |
| GSM1246216_treat | 0.013138 | 0 | 0.052376 | 0.068058 | 0 | 0.174312 | 0 | 0.023927 | 0.035225 | 0.001921 | 0.016491 | 0 | 0.00124 | 0.121565 | 0.047087 | 0.36854 | 0.008457 | 0 | 0.014416 | 0.030402 | 0 | 0.022846 |
| GSM1246217_treat | 0 | 0.000943 | 0.059535 | 0.045987 | 0 | 0.085517 | 0 | 0.012763 | 0.004624 | 0.080651 | 0 | 0.01857 | 0 | 0.083628 | 0.076569 | 0.402423 | 0.007081 | 0 | 0.052718 | 0.020406 | 0 | 0.048586 |
| GSM1246218_treat | 0.032266 | 0.016007 | 0.092508 | 0.020255 | 0 | 0.025335 | 0 | 0.064473 | 0.029848 | 0.02218 | 0.00921 | 0 | 0.010682 | 0.046801 | 0.070148 | 0.373805 | 0.059832 | 0 | 0.071577 | 0.020506 | 0 | 0.034568 |
