# Supplementary material for: Development and analysis of a comprehensive diagnostic model for aortic valve calcification using machine learning methods and artificial neural networks
Source: Front Cardiovasc Med. 2022 Dec 1;9:913776. doi: 10.3389/fcvm.2022.913776 (PMC9751025; doi:10.3389/fcvm.2022.913776)
Supplement: Supplementary file 9 [file Table_8.docx]

SUPPLEMENTARY TABLE 8: Principal component analysis (PCA) analysis results of immune cell infiltration of merged data sets of GSE12644 and GSE51472 between normal and aortic valve calcification (AVC) samples.

| Samples | PC1 | PC2 | PC3 | PC4 | PC5 | PC6 | PC7 | PC8 | PC9 | PC10 | PC11 | PC12 | PC13 | PC14 | PC15 | PC16 | PC17 | PC18 | PC19 | PC20 | PC21 | PC22 |
| --- | --- | --- | --- | --- | --- | --- | --- | --- | --- | --- | --- | --- | --- | --- | --- | --- | --- | --- | --- | --- | --- | --- |
| GSM317342 | 1.7302661 | -2.0519406 | 0.3798112 | 3.0979718 | 0.8664427 | 0.0219541 | -0.1430228 | 1.3416367 | -2.3870057 | -0.6077361 | -0.000848 | -0.0804708 | -0.204814 | -0.4302436 | 0.0596164 | 0.6044878 | 0.3684035 | -0.6577716 | 0.2408083 | -0.317134 | -0.0643744 | 4.36E-09 |
| GSM317343 | 4.51085 | 0.7474196 | -4.4220802 | -1.144276 | 2.0820928 | -2.846226 | -1.408968 | -0.1450251 | 0.6552105 | -1.3041582 | -0.266043 | 1.3860925 | -0.3392667 | 0.0157686 | 0.4916219 | 0.092483 | -0.222753 | -0.0699057 | -0.0063962 | -0.051742 | 0.0242161 | 8.88E-16 |
| GSM317344 | 2.5845346 | 1.1756435 | 1.7255243 | 1.2521119 | 1.3197438 | -0.3395523 | 0.3793068 | -0.135228 | -1.6147996 | 0.8284281 | -0.9347822 | 0.3882781 | 0.3311981 | 0.8184895 | 0.1982218 | -1.3042271 | -0.1935793 | 0.19011 | -0.2354533 | -0.1191194 | 0.2236733 | 3.21E-09 |
| GSM317345 | 3.0133579 | 0.7825372 | -3.586605 | -1.2086303 | -1.9997755 | 4.5860701 | 0.1876092 | 0.8978885 | -0.8684664 | -0.2689094 | 0.6848612 | 0.7291352 | -0.1618965 | 0.2170186 | 0.1631429 | -0.4047509 | -0.2728598 | 0.0454791 | 0.0703347 | -0.0994643 | -0.0214138 | -8.26E-16 |
| GSM317346 | 2.0270982 | 1.9973999 | -1.6582317 | -0.5557248 | 0.5010507 | 0.4845228 | -0.2109325 | 0.5287947 | -0.5439496 | 0.5020496 | -1.5059878 | -1.2394752 | 0.5899155 | 0.1780642 | -0.4097592 | 0.8678371 | 0.6791898 | 0.3259853 | 0.0973622 | 0.708214 | 0.297248 | 3.64E-09 |
| GSM377368 | -2.4034127 | 0.7328687 | -0.1662414 | 0.3427095 | -1.6358823 | -1.1174494 | -0.375824 | 0.4573306 | -0.8773335 | 1.1224905 | 0.0635861 | 0.516367 | 0.3942033 | 0.7225438 | 0.8670216 | 0.0514202 | -0.0565086 | -0.0128145 | -0.3219321 | -0.0467614 | 0.2520004 | -6.38E-09 |
| GSM377369 | -2.7652667 | 0.1415791 | -0.9290707 | 0.3680936 | -1.3854149 | -0.8494884 | -0.2796689 | 0.123073 | -0.5569436 | 0.9003327 | -0.08021 | -0.0550884 | -0.0710064 | -0.0619404 | 0.15756 | 0.3170246 | -1.0618205 | -0.2077344 | -0.1081735 | 0.3230454 | 0.0506946 | 1.30E-08 |
| GSM377370 | -1.1859225 | 1.5196923 | -0.1834661 | 0.2119322 | -0.6492483 | 0.0979667 | 0.4127453 | 0.2380926 | 0.3480991 | -0.2671078 | -0.63625 | 0.1544845 | -0.2637138 | -1.9672792 | -0.1530783 | -0.233901 | 0.3406206 | -0.4644678 | -0.0523277 | -0.09013 | 0.1893432 | -6.23E-09 |
| GSM377371 | -2.4362361 | 1.0997825 | 0.2838746 | -0.7774272 | -1.1706963 | -0.7361643 | -0.3344721 | 0.3491818 | 0.1135015 | -1.0605534 | 0.3836026 | 0.1551774 | 0.2676768 | 0.4965429 | 0.2826193 | -0.8116968 | 1.3782786 | -0.0429687 | 0.3663573 | 0.1282546 | -0.1713453 | 8.20E-09 |
| GSM377372 | -3.5324622 | -0.4073355 | -0.4741572 | 0.8418836 | 0.9412258 | 0.2354989 | -1.8350676 | 1.1500263 | -0.0118094 | -0.079894 | 0.5239842 | -0.8081301 | -2.0581271 | 0.7528547 | -0.5141833 | -0.0901138 | -0.1811941 | -0.152993 | 0.1367828 | 0.2961399 | 0.0580166 | -7.21E-09 |
| GSM1246204 | -0.0104589 | 2.8198099 | 1.5110364 | 0.7844852 | 1.5419545 | 1.0874748 | -1.0033673 | 1.1080298 | -0.1537761 | -0.4524672 | -1.1286105 | -0.1850997 | 0.318131 | 0.1557426 | 0.3588286 | 0.3869225 | 0.1365347 | 0.4154999 | -0.3024781 | -0.3707526 | -0.3435235 | -2.02E-09 |
| GSM1246205 | 1.2063763 | 2.2264834 | 1.1764821 | -1.100407 | 0.1749992 | -0.1507477 | 1.4107137 | -1.652533 | -0.9187384 | 0.0477968 | 0.9314935 | -0.0886957 | -0.656142 | 0.5538196 | 0.4337389 | 1.0181275 | 0.2302443 | -0.2805348 | 0.200756 | -0.1572655 | -0.3809009 | -9.26E-10 |
| GSM1246206 | -0.288133 | 2.577597 | 1.5109162 | 0.2641346 | 0.3358019 | 0.2410904 | 0.4021186 | 0.0919966 | -0.0481653 | -1.1546153 | -0.8287074 | 0.1040545 | 0.38915 | -0.5778632 | -0.3586141 | -0.0556431 | -0.8863243 | 0.3698122 | 0.2674182 | 0.2641215 | -0.2120926 | 1.19E-09 |
| GSM1246207 | -0.1991805 | 2.52379 | 0.1464247 | -0.9199262 | -0.2108212 | 0.0152233 | 0.0128391 | -0.1428501 | 0.3408608 | 0.1223341 | -0.3594924 | -0.3656422 | -0.1335932 | -0.4159608 | -0.3858392 | -0.0394263 | -0.0707208 | -0.3886751 | -0.1013327 | 0.2220201 | -0.1239531 | -8.15E-09 |
| GSM1246208 | -0.5851786 | 1.738313 | 1.68271 | 2.8869341 | 1.1762008 | 1.8761111 | -1.7889203 | -1.211515 | 2.2222053 | 0.0547034 | 1.137802 | 1.3776514 | 0.6074123 | 0.2642026 | -0.0606974 | 0.3277321 | 0.0960101 | -0.0312602 | 0.0595946 | 0.2556398 | 0.1611407 | 1.57E-09 |
| GSM317347 | 2.1891376 | -0.8820842 | -0.1335065 | 1.165825 | 0.2854943 | -0.5309154 | 1.2593188 | -0.6702703 | -0.4291506 | -0.7378061 | 2.1048127 | -0.6717635 | -0.2030692 | -0.2741757 | -0.0955788 | 0.2348131 | 0.0687757 | 0.7370777 | -0.2205837 | 0.1282222 | 0.2861086 | 4.67E-09 |
| GSM317348 | 4.563137 | -2.1102255 | 2.0810382 | -0.4825068 | -3.0034919 | -0.4064355 | -2.3742517 | -0.9194523 | 0.1669488 | -0.7919088 | -0.210797 | -0.9377104 | -0.5360217 | -0.1285533 | -0.2430664 | 0.0052093 | -0.0270742 | 0.5095408 | -0.4587753 | -0.0964889 | -0.117836 | -3.55E-10 |
| GSM317349 | 3.3837456 | -1.4402017 | 0.4286163 | 1.3824593 | -1.1734953 | -0.5049941 | -0.346683 | -0.359942 | -0.2910731 | 0.3098989 | 0.3233662 | -0.1492707 | 0.6889309 | -0.2299312 | -0.0039562 | -0.2499427 | 0.032341 | -0.5971858 | 0.0249303 | 0.2184261 | 0.2012589 | -1.25E-08 |
| GSM317350 | 2.1105186 | -2.8774588 | 2.5590502 | -1.6421983 | -0.9338725 | 0.2033447 | -0.8984358 | -0.0922671 | 0.751556 | 1.3486116 | -1.0057623 | 0.7632655 | -0.4734172 | -0.2082991 | 0.4048784 | 0.2621604 | -0.0226646 | 0.0540393 | 0.8769159 | -0.2036782 | 0.1144972 | 4.72E-09 |
| GSM317351 | 1.6281707 | -3.5914045 | 1.9874098 | -1.7439247 | 1.1691355 | 0.0726551 | 0.4453177 | 1.447779 | 0.2068526 | -0.0623609 | 0.5351022 | 0.6747436 | 0.5286226 | 0.16489 | -0.4334826 | -0.2927558 | 0.0162539 | -0.5707156 | -0.5424556 | 0.5828626 | -0.4109149 | 1.96E-09 |
| GSM377373 | -4.5995897 | -3.6555255 | -1.1907766 | -0.7690625 | 1.218589 | 1.0774034 | -1.0635361 | -2.5977932 | -0.9979162 | -1.3632155 | -0.8013682 | -0.580849 | 0.7272415 | 0.4706321 | -0.3868754 | -0.1961604 | -0.2156674 | -0.2908637 | 0.0662111 | -0.3178354 | 0.0897497 | 7.71E-10 |
| GSM377374 | -2.8457286 | 0.1714384 | -0.2389821 | 1.1539817 | -1.478911 | -1.1730361 | -0.0815628 | 0.37055 | -0.882687 | 0.0204647 | 0.5177558 | 0.7738281 | 0.2674205 | -0.0894072 | 0.0744344 | 0.1906857 | -0.2721432 | 0.4535043 | 0.177626 | -0.0057071 | -0.3408306 | -3.19E-09 |
| GSM377375 | -2.2421709 | 0.5759946 | -0.5810114 | 0.3140848 | -0.9185242 | -0.3244355 | -0.2434501 | 0.1029592 | 0.1860664 | 0.2640015 | 0.1611628 | 0.0922191 | -0.142298 | -0.6996395 | 0.2191253 | -0.0895737 | 0.1203423 | -0.1833902 | -0.0340552 | 0.1698871 | 0.0732568 | 1.04E-08 |
| GSM377376 | -2.5012417 | -1.866359 | -0.2166872 | -0.8899616 | -0.1655631 | -0.5147741 | 0.8487455 | -0.3426477 | -1.0125466 | -0.0073774 | -0.2619546 | 1.5626412 | -0.0610419 | -0.2446344 | -1.2361094 | 0.0547597 | 0.2558269 | 0.6802308 | 0.0695943 | 0.2187842 | -0.021972 | -4.23E-09 |
| GSM377377 | -2.4447237 | 0.2513068 | -0.5712445 | -0.6761288 | -0.4686418 | -0.0736216 | -0.2042547 | -0.6509081 | 0.1698477 | -0.2813372 | 0.2309199 | -0.6942117 | 0.5597074 | -0.3124076 | 1.2544765 | -0.269744 | 0.3567068 | 0.1748078 | -0.1192213 | 0.1038253 | -0.0807762 | -6.67E-09 |
| GSM1246209 | -0.5825321 | 2.082644 | 1.1202948 | -0.5966205 | -0.2765167 | -0.2987994 | 0.72363 | 0.2008448 | 0.5207593 | -0.667526 | 0.1087213 | -0.5013542 | 0.0341989 | -0.2017719 | 0.1394242 | -0.0859337 | -0.7033959 | -0.1752836 | 0.012974 | -0.2702487 | -0.0217457 | -5.17E-10 |
| GSM1246210 | 1.0912999 | 1.4704851 | -1.5664054 | 0.156916 | -0.2939856 | -0.8536456 | -0.3645924 | 0.0228825 | 0.7351044 | 1.0288093 | 0.9179081 | -0.73306 | 1.0541396 | -0.2781687 | -1.3701325 | -0.2213363 | 0.0442974 | -0.17094 | 0.0991847 | -0.6767492 | -0.1023002 | 5.81E-09 |
| GSM1246211 | 0.9163641 | 3.113118 | 1.4759653 | -1.3092523 | 0.1654393 | 0.046537 | 1.1745171 | -1.9169959 | -0.3287844 | 0.1158598 | -0.1060258 | 0.2366668 | -0.9579446 | 0.4758415 | -0.3355079 | -0.2211479 | 0.0650677 | -0.4813343 | -0.1546849 | 0.0795133 | 0.2335838 | 2.79E-09 |
| GSM1246212 | 0.3230322 | -2.660273 | -0.9887823 | 2.4033229 | -1.6628051 | 0.0271835 | 2.7519466 | 0.1244294 | 2.204101 | -1.3575037 | -1.3873588 | -0.1353935 | -0.0735233 | 0.88248 | 0.1924821 | 0.2306794 | -0.0647736 | -0.1592972 | -0.0420113 | 0.0270327 | 0.005151 | 1.25E-09 |
| GSM1246213 | -1.7717792 | -1.1303914 | 1.1452784 | -2.3485276 | 0.4300885 | 0.1954168 | 0.4490338 | 1.2364821 | 0.2490292 | -0.1901151 | 0.4048309 | 0.4886083 | 0.6485544 | 0.2084669 | -0.0687744 | 1.0881104 | 0.0148197 | 0.0425378 | -0.2844928 | -0.4324142 | 0.4588458 | -4.88E-09 |
| GSM1246214 | -0.2603828 | -2.7460272 | -0.8022761 | -0.7193437 | 2.1538951 | 0.9175753 | 0.180844 | -0.7670448 | 0.3177293 | 1.2551906 | 0.276384 | -0.7846848 | 0.1478712 | -0.9049993 | 0.9667545 | 0.0231873 | -0.1616567 | 0.0624643 | -0.1457086 | 0.216916 | -0.1925037 | 3.85E-09 |
| GSM1246215 | 0.105023 | 0.0763653 | -1.3888661 | -0.2758811 | -0.1681924 | -0.5746195 | 0.0391701 | 0.5897311 | 1.0721091 | 1.4123649 | 0.026665 | -0.7400364 | 0.3053995 | 1.1955735 | -0.3557906 | 0.0612292 | -0.0362555 | -0.0920569 | 0.0826455 | -0.1241714 | -0.2487712 | -2.62E-09 |
| GSM1246216 | -1.7781705 | -0.2998044 | 0.7902215 | -0.7565266 | 0.91811 | 0.1357631 | 0.3246189 | 1.5518758 | 0.9839788 | -0.2078786 | 0.083263 | -0.2381451 | -0.9236145 | -0.2086966 | 0.1070714 | -0.4652893 | 0.1815713 | 0.2233344 | -0.3350905 | -0.4101026 | 0.2157008 | 6.86E-09 |
| GSM1246217 | 1.1149629 | -0.8309858 | 0.7010645 | -0.341001 | 1.492584 | -0.3794063 | 0.9597463 | 0.7762108 | 0.2212662 | -0.2087176 | 0.7674682 | -0.8647128 | 0.2731158 | 0.0549722 | 0.2072599 | -0.6305681 | -0.3459718 | 0.3459453 | 0.8367926 | 0.0942973 | 0.145249 | -7.52E-09 |
| GSM1246218 | -0.065304 | -1.2742515 | -1.607328 | 1.6304805 | 0.8229905 | 0.3525199 | 0.9947888 | -1.1053228 | 0.4579194 | 1.7378518 | -0.6694919 | 0.450581 | -0.8733992 | -0.3939315 | -0.1668323 | -0.1546585 | 0.4100788 | 0.3998241 | -0.2211157 | -0.2474371 | -0.2244804 | -4.84E-09 |
