# Supplementary material for: Development and analysis of a comprehensive diagnostic model for aortic valve calcification using machine learning methods and artificial neural networks
Source: Front Cardiovasc Med. 2022 Dec 1;9:913776. doi: 10.3389/fcvm.2022.913776 (PMC9751025; doi:10.3389/fcvm.2022.913776)
Supplement: Supplementary file 10 [file Table_9.docx]

SUPPLEMENTARY TABLE 9: Results of correlation analysis of five markers and

infiltration immune cells.

| Genes | Cell | Correlation | Pvalue |
| --- | --- | --- | --- |
| BEX2 | B cells naive | 0.304944676 | 0.074871 |
| BEX2 | B cells memory | -0.252102073 | 0.144013 |
| BEX2 | Plasma cells | -0.250490343 | 0.146681 |
| BEX2 | T cells CD8 | -0.104835345 | 0.548943 |
| BEX2 | T cells CD4 naive | -0.006634265 | 0.969828 |
| BEX2 | T cells CD4 memory resting | 0.093837535 | 0.590554 |
| BEX2 | T cells CD4 memory activated | -0.266141099 | 0.122261 |
| BEX2 | T cells follicular helper | 0.158204924 | 0.364039 |
| BEX2 | T cells regulatory (Tregs) | -0.155813593 | 0.371426 |
| BEX2 | T cells gamma delta | 0.007532569 | 0.965745 |
| BEX2 | NK cells resting | -0.094052868 | 0.590984 |
| BEX2 | NK cells activated | 0.282019388 | 0.100721 |
| BEX2 | Monocytes | 0.309620872 | 0.070298 |
| BEX2 | Macrophages M0 | -0.294466949 | 0.085956 |
| BEX2 | Macrophages M1 | -0.09719888 | 0.577253 |
| BEX2 | Macrophages M2 | 0.200280112 | 0.247653 |
| BEX2 | Dendritic cells resting | -0.014426781 | 0.934444 |
| BEX2 | Dendritic cells activated | 0.067923561 | 0.698241 |
| BEX2 | Mast cells resting | 0.220229765 | 0.203638 |
| BEX2 | Mast cells activated | -0.124010047 | 0.477855 |
| BEX2 | Eosinophils | 0.254713354 | 0.139767 |
| BEX2 | Neutrophils | -0.138655462 | 0.425486 |
| CXCL16 | B cells naive | -0.504320679 | 0.002007 |
| CXCL16 | B cells memory | 0.573948984 | 0.000312 |
| CXCL16 | Plasma cells | 0.478565443 | 0.003636 |
| CXCL16 | T cells CD8 | 0.120112153 | 0.491906 |
| CXCL16 | T cells CD4 naive | -0.083013368 | 0.63543 |
| CXCL16 | T cells CD4 memory resting | -0.340616246 | 0.045852 |
| CXCL16 | T cells CD4 memory activated | 0.206322719 | 0.234385 |
| CXCL16 | T cells follicular helper | 0.141655118 | 0.416956 |
| CXCL16 | T cells regulatory (Tregs) | 0.06375492 | 0.715969 |
| CXCL16 | T cells gamma delta | 0.188314228 | 0.278653 |
| CXCL16 | NK cells resting | 0.227096006 | 0.189539 |
| CXCL16 | NK cells activated | -0.556656858 | 0.000515 |
| CXCL16 | Monocytes | -0.321240511 | 0.059879 |
| CXCL16 | Macrophages M0 | 0.636377553 | 3.97E-05 |
| CXCL16 | Macrophages M1 | -0.256302521 | 0.137001 |
| CXCL16 | Macrophages M2 | -0.482352941 | 0.003699 |
| CXCL16 | Dendritic cells resting | 0.043700539 | 0.803155 |
| CXCL16 | Dendritic cells activated | -0.118866232 | 0.496441 |
| CXCL16 | Mast cells resting | -0.33678903 | 0.047891 |
| CXCL16 | Mast cells activated | 0.482326137 | 0.003344 |
| CXCL16 | Eosinophils | -0.271694244 | 0.11437 |
| CXCL16 | Neutrophils | 0.214845938 | 0.214365 |
| GPM6A | B cells naive | 0.338386564 | 0.046777 |
| GPM6A | B cells memory | -0.245808027 | 0.154634 |
| GPM6A | Plasma cells | -0.505743926 | 0.00194 |
| GPM6A | T cells CD8 | 0.169586587 | 0.330091 |
| GPM6A | T cells CD4 naive | -0.160583237 | 0.356779 |
| GPM6A | T cells CD4 memory resting | 0.208963585 | 0.227412 |
| GPM6A | T cells CD4 memory activated | -0.515046394 | 0.001546 |
| GPM6A | T cells follicular helper | 0.01514728 | 0.931178 |
| GPM6A | T cells regulatory (Tregs) | 0.190835914 | 0.272148 |
| GPM6A | T cells gamma delta | -0.358851593 | 0.034262 |
| GPM6A | NK cells resting | 0.008631434 | 0.960751 |
| GPM6A | NK cells activated | 0.41082632 | 0.014225 |
| GPM6A | Monocytes | 0.156298317 | 0.369922 |
| GPM6A | Macrophages M0 | -0.571854183 | 0.000332 |
| GPM6A | Macrophages M1 | -0.231652661 | 0.179987 |
| GPM6A | Macrophages M2 | 0.435294118 | 0.009512 |
| GPM6A | Dendritic cells resting | 0.025071784 | 0.88632 |
| GPM6A | Dendritic cells activated | 0.152828012 | 0.380774 |
| GPM6A | Mast cells resting | 0.391706376 | 0.019962 |
| GPM6A | Mast cells activated | -0.37232193 | 0.027626 |
| GPM6A | Eosinophils | 0.186789793 | 0.282634 |
| GPM6A | Neutrophils | -0.212044818 | 0.220511 |
| S100A9 | B cells naive | -0.527276551 | 0.001135 |
| S100A9 | B cells memory | 0.406221154 | 0.015461 |
| S100A9 | Plasma cells | 0.501821258 | 0.002131 |
| S100A9 | T cells CD8 | 0.014155575 | 0.935674 |
| S100A9 | T cells CD4 naive | 0.031130013 | 0.859098 |
| S100A9 | T cells CD4 memory resting | -0.318487395 | 0.062693 |
| S100A9 | T cells CD4 memory activated | 0.290474 | 0.090498 |
| S100A9 | T cells follicular helper | 0.065357708 | 0.709135 |
| S100A9 | T cells regulatory (Tregs) | 0.039882562 | 0.820058 |
| S100A9 | T cells gamma delta | 0.289551957 | 0.091573 |
| S100A9 | NK cells resting | 0.174414495 | 0.316303 |
| S100A9 | NK cells activated | -0.492178066 | 0.002671 |
| S100A9 | Monocytes | -0.341362325 | 0.044758 |
| S100A9 | Macrophages M0 | 0.661997127 | 1.48E-05 |
| S100A9 | Macrophages M1 | -0.061064426 | 0.726704 |
| S100A9 | Macrophages M2 | -0.498319328 | 0.00261 |
| S100A9 | Dendritic cells resting | 0.074094825 | 0.672285 |
| S100A9 | Dendritic cells activated | -0.169808903 | 0.329449 |
| S100A9 | Mast cells resting | -0.48725135 | 0.002991 |
| S100A9 | Mast cells activated | 0.532221944 | 0.000999 |
| S100A9 | Eosinophils | -0.220751574 | 0.202542 |
| S100A9 | Neutrophils | 0.284033613 | 0.098341 |
| SCARA5 | B cells naive | 0.445939077 | 0.007254 |
| SCARA5 | B cells memory | -0.410133669 | 0.014405 |
| SCARA5 | Plasma cells | -0.431213242 | 0.009703 |
| SCARA5 | T cells CD8 | -0.042606878 | 0.807989 |
| SCARA5 | T cells CD4 naive | -0.01054678 | 0.952051 |
| SCARA5 | T cells CD4 memory resting | 0.238095238 | 0.167919 |
| SCARA5 | T cells CD4 memory activated | -0.288446258 | 0.092874 |
| SCARA5 | T cells follicular helper | -0.168864121 | 0.332186 |
| SCARA5 | T cells regulatory (Tregs) | 0.093202259 | 0.59436 |
| SCARA5 | T cells gamma delta | -0.220553624 | 0.202957 |
| SCARA5 | NK cells resting | -0.218762208 | 0.206743 |
| SCARA5 | NK cells activated | 0.59507296 | 0.000163 |
| SCARA5 | Monocytes | 0.200934735 | 0.247099 |
| SCARA5 | Macrophages M0 | -0.737906974 | 4.23E-07 |
| SCARA5 | Macrophages M1 | 0.223809524 | 0.195502 |
| SCARA5 | Macrophages M2 | 0.535294118 | 0.001093 |
| SCARA5 | Dendritic cells resting | 0.052244555 | 0.765656 |
| SCARA5 | Dendritic cells activated | 0.186789793 | 0.282634 |
| SCARA5 | Mast cells resting | 0.472121061 | 0.00419 |
| SCARA5 | Mast cells activated | -0.541850959 | 0.000774 |
| SCARA5 | Eosinophils | 0.101885342 | 0.560304 |
| SCARA5 | Neutrophils | -0.19719888 | 0.255119 |
